# *In Vitro* and *In Vivo* Biological Activities of Dipicolinate Oxovanadium(IV) Complexes

**DOI:** 10.1021/acs.jmedchem.3c00255

**Published:** 2023-06-13

**Authors:** Katarzyna Choroba, Beatriz Filipe, Anna Świtlicka, Mateusz Penkala, Barbara Machura, Alina Bieńko, Sandra Cordeiro, Pedro V. Baptista, Alexandra R. Fernandes

**Affiliations:** † University of Silesia, Institute of Chemistry, Szkolna 9, 40-006 Katowice, Poland; ‡ Associate Laboratory i4HB - Institute for Health and Bioeconomy, NOVA School of Science and Technology, NOVA University Lisbon, 2819-516 Caparica, Portugal; § UCIBIO − Applied Molecular Biosciences Unit, Department of Life Sciences, NOVA School of Science and Technology, NOVA University Lisbon, 2819-516 Caparica, Portugal; ∥ Faculty of Chemistry, University of Wroclaw, F. Joliot-Curie 14, 50-383 Wroclaw, Poland

## Abstract

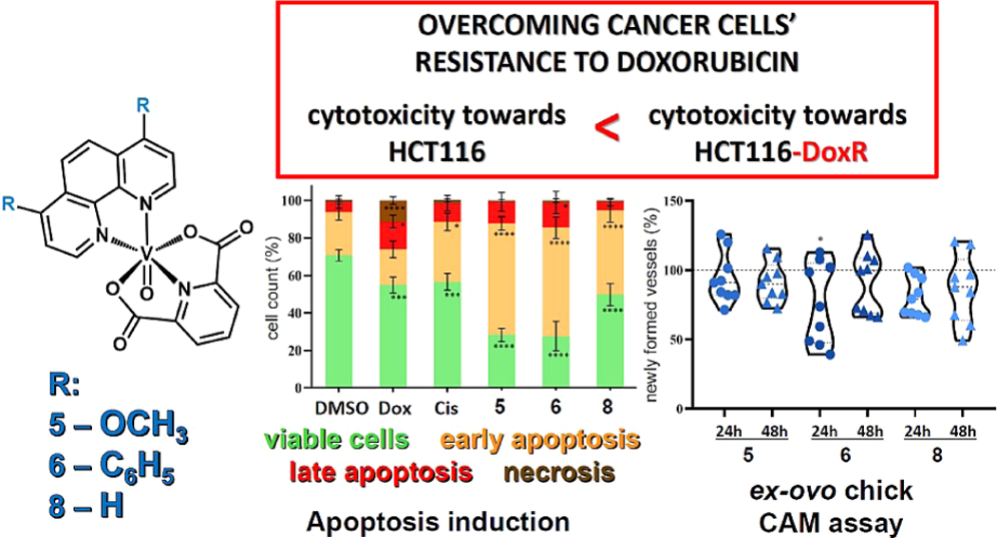

The work is focused on anticancer properties of dipicolinate (dipic)-based vanadium(IV) complexes [VO(dipic)(N^∩^N)] bearing different diimines (2-(1*H*-imidazol-2-yl)pyridine, 2-(2-pyridyl)benzimidazole, 1,10-phenanthroline-5,6-dione, 1,10-phenanthroline, and 2,2′-bipyridine), as well as differently 4,7-substituted 1,10-phenanthrolines. The antiproliferative effect of V(IV) systems was analyzed in different tumors (A2780, HCT116, and HCT116-DoxR) and normal (primary human dermal fibroblasts) cell lines, revealing a high cytotoxic effect of [VO(dipic)(N^∩^N)] with 4,7-dimethoxy-phen (**5**), 4,7-diphenyl-phen (**6**), and 1,10-phenanthroline (**8**) against HCT116-DoxR cells. The cytotoxicity differences between these complexes can be correlated with their different internalization by HCT116-DoxR cells. Worthy of note, these three complexes were found to (i) induce cell death through apoptosis and autophagy pathways, namely, through ROS production; (ii) not to be cytostatic; (iii) to interact with the BSA protein; (iv) do not promote tumor cell migration or a pro-angiogenic capability; (v) show a slight *in vivo* anti-angiogenic capability, and (vi) do not show *in vivo* toxicity in a chicken embryo.

## Introduction

Vanadium compounds have been widely investigated due to a broad application in catalysis^1−9^ and a wide range of pharmacological properties.^10−19^ In the area of medicinal applications, the exploration of vanadium-based compounds has been focused on their insulin-mimetic properties,^11−15,20−25^ anticancer activities,^11−19,26−40^ antibacterial action,^12,14,15,41−43^ and effects on enzymes.^12,14,15,44^ In particular, bis(ethylmaltolato)oxidovanadium(IV) (BEOV) entered the phase IIa clinical trials as an antidiabetic agent and its improved pharmacokinetics relative to vanadyl sulfate was evidenced.^45−47^ Vanadocene dichloride, (η^5^-C_5_H_5_)_2_VCl_2_ (C_5_H_5_ = cyclopentadienyl), was demonstrated to have antitumor effects on a wide spectrum of cancer cells, including testicular cancer, leukemia, breast cancer, glioblastoma, and colon cancer.^11−19,48−50^ Among oxidovanadium(IV) derivatives, special attention has been devoted to bis(4,7-dimethyl-1,10-phenanthroline)-sulfatooxidovanadium(IV) (Metvan), which was found to induce apoptosis in leukemia, multiple myeloma, and solid tumors such as breast, prostate, testis, and glioblastoma. Most remarkably, Metvan is known to show high activity against cisplatin-resistant ovarian and testis tumor cell lines.^51−55^ As reported, the anticancer activity of vanadium coordination compounds can be assigned to different mechanisms, including DNA binding, generating reactive oxygen species leading to oxidative stress, cell cycle arrest, and programmed cell death,^19^ and it is widely modulated by the ligand nature and geometry of the complex.^11−19^

Regarding prospective applications of vanadium compounds as therapeutic agents, there is a strong need to develop new biologically active systems and establish reliable structure–property relationships essential for their rational design.

Scientific attention of the current work is focused on anticancer properties of a series of dipicolinate (dipic)-based vanadium(IV) complexes [VO(dipic)(N^∩^N)]. Previously, Pramanik and Chakrabarti et al. demonstrated the impact of the number of methylene groups in the alkoxo chain (R) on the anticancer activity of [VO(OR)(dipic)(H_2_O)] (R = R = Et, n-Pr, and n-Bu) against human breast adenocarcinoma (MCF-7) cell lines,^56^ as well as they showed the possibility of the enhancement of the antiproliferative behavior of [VO(OR)(dipic)(L)_2_] (L = imidazole-based monodentate ligand) against human hepatic carcinoma (Hep3B) cell lines by the introduction of methyl and allyl substituents into the imidazole core.^39^

Our studies are focused on the investigations of the diimine core effect and the role of substituents introduced into 4,7-positions of 1,10-phenanthroline (phen). The following diimine ligands 2-(1*H*-imidazol-2-yl)pyridine (pyim), 2-(2-pyridyl)benzimidazole (pybim), 1,10-phenanthroline-5,6-dione (phendione), 1,10-phenanthroline (phen), and 2,2′-bipyridine (bipy) have been coordinated to the unit {VO(dipic)}, and the resulting V(IV) complexes were compared with [VO(dipic)(im)_2_]. In the next step, antiproliferative properties of the most active system [VO(dipic)(phen)] have been further modified by the introduction of chloro, methoxy, phenyl, and phenothiazine groups into 4,7-positions of 1,10-phenanthroline. All complexes that are first obtained—[VO(dipic)(pyim)] (**1**), [VO(dip)(pybim)] (**2**), [VO(dipic)(phendione)] (**3**), [VO(dipic)(4,7-Cl_2_-phen)] (**4**), [VO(dipic)(4,7-(CH_3_O)_2_-phen)] (**5**), [VO(dipic)(4,7-Ph_2_-phen)] (**6**), [VO(dipic)(4,7-(phtiaz)_2_-phen)] (**7**)—and those previously synthesized—[VO(dipic)(phen)] (**8**),^57−59^ [VO(dipic)(bipy)] (**9**, bipy = 2,2′-bipyridine),^59,60^ and [VO(dipic)(im)_2_] (**10**, im = imidazole)^39^—were analyzed in different tumors (A2780, HCT116, and HCT116-DoxR) and normal (primary human dermal fibroblasts) cell lines. Concerning cell lines choice, CRC (HCT116 cell line) has a high incidence all over the world with a high percentage of patients developing resistance to common therapeutic regimens, meaning that studying new complexes in resistance models is of high importance. HCR116-DoxR cell line is a CRC cell line derived from parental HCT116 with doxorubicin resistance. Fibroblasts are part of an epithelial tissue and highly important for the tumor microenvironment and were chosen as the comparative normal human cell line in a way to guarantee that the complexes would not damage those types of cells. The cytotoxic potential of the complexes was also studied in the A2780 cell line to understand if the complexes are specific anticancer agents for CRC or if they can also be used as therapeutic agents for other types of epithelial cancers.

Interestingly, vanadium complexes revealed a higher selectivity for colorectal carcinoma resistant to doxorubicin. To understand their biological activity, several *in vitro* and *in vivo* studies were performed. As *in vivo* models for toxicity assessment and anti-angiogenic potential, an *ex ovo* chick chorioallantoic membrane (CAM) assay was used.

## Results and Discussion

### Synthesis, Molecular Structure, and Spectroscopic Characterization

To synthesize dipicolinate vanadium(IV) complexes [VO(dipic)(N^∩^N)], the procedure based on the reaction of [VO(dipic)(H_2_O)_2_] with an appropriate N-donor ligand was employed. The vanadium(IV) precursor [VO(dipic)(H_2_O)_2_] was obtained according to the literature method based on the reaction of vanadyl sulfate with dipicolinic acid.^39,56^ Identity and purity of [VO(dipic)(N^∩^N)] were confirmed by elemental analysis, FT-IR technique, X-ray analysis, UV–vis, and EPR spectroscopies.

**Figure 1:**
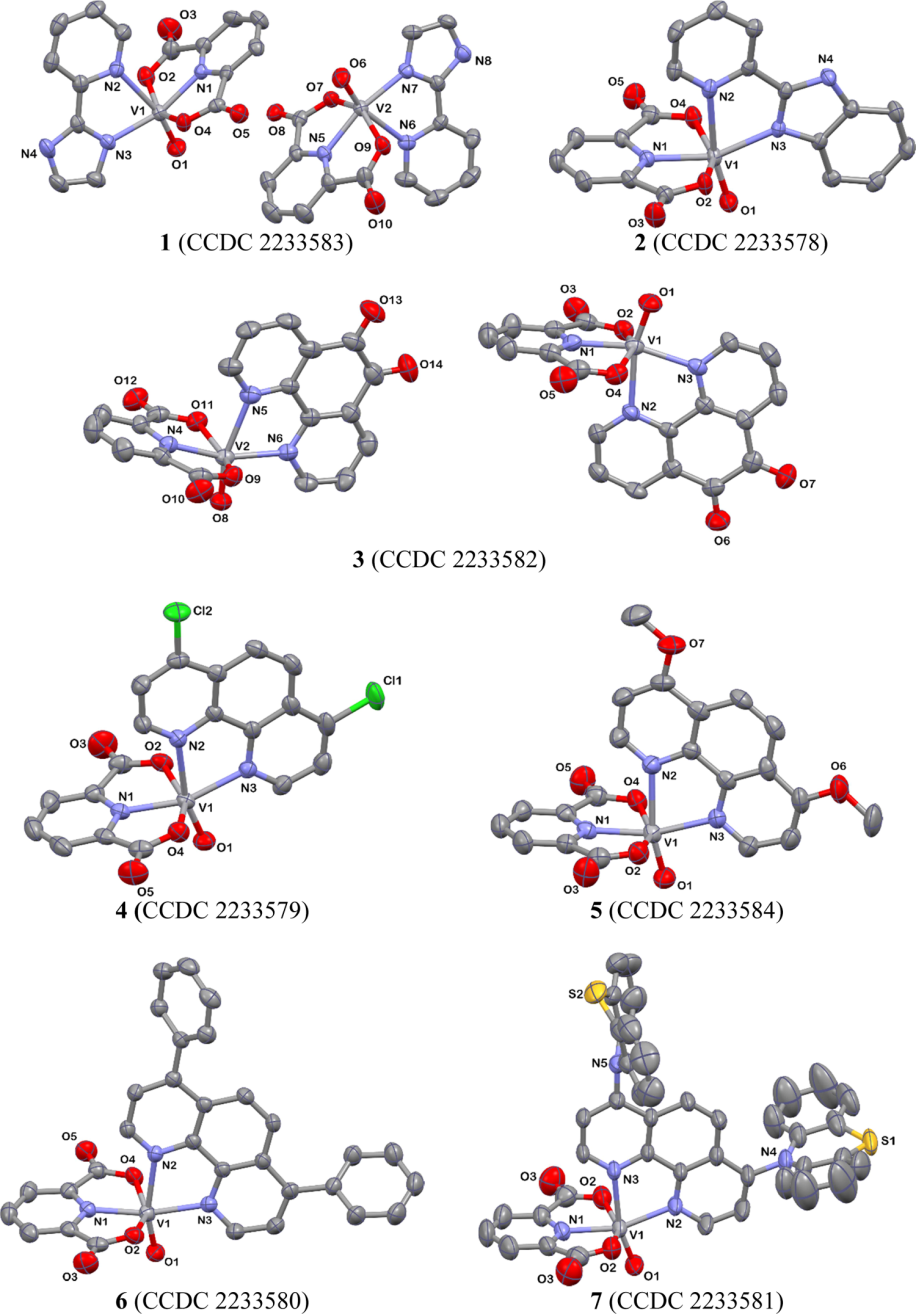
Perspective views showing the asymmetric units of **1**–**7** along with the numbering of the noncarbon atoms. Displacement ellipsoids are drawn at the 50% probability level. Hydrogen atoms and solvent molecules are omitted for clarity.

The perspective views showing the asymmetric units of **1**–**7** along with the atom numbering are presented in Figure 1. The vanadium(IV) ion of [VO(dipic)(N^∩^N)] adopts a highly distorted octahedral coordination geometry defined by pyridine nitrogen and carboxylate oxygen atoms of the deprotonated dipicolinic acid, two nitrogen atoms of the diamine ligand, and multiple bonded oxo ligand. Tridentate and bidentate chelating coordination modes of dipic and N^∩^N ligands result in the formation of five-membered metallocycles with bite angles significantly lower than 90°, falling in the ranges of 76.39(8)–77.31(6) and 71.91(19)–73.36(14)° for N_dipic_–V–O_dipic_ and N_L_–V–N_L_, respectively. The repulsion exerted by the V=O unit leads to an enlargement of the angles O_oxo_–V–N_cis_ [90.9(2)–108.65(15)°] and O_oxo_–V–O_cis_ [97.3(2)–103.42(10)°]. The structural manifestation of the *trans* effect of the multiply oxo bonded ligand is also seen in a noticeable elongation of the V–N_trans_ bond length [2.2979(16)–2.393(2)Å] relative to other V–N ones [2.016(5)–2.1343(18)Å] (Tables S2–S8). Structural parameters concerning the dipicolinate ion chelated to the metal center are nearly identical in all reported structures (Tables S2–S8), likewise, as the V=O distance, which falls in a narrow range from 1.579(2)Å (**2**) to 1.595(4) (**3**). In complexes [VO(dipic)(4,7-R_2_-phen)] (**4**–**6**), the V–N bond length in the *trans* position to V=O unit is impacted by the electronic changes in the substituent group introduced into the phen backbone. The attachment of the electron-withdrawing chloride ion results in the elongation of this bond. Additional structural data of **1**–**7** are available in Tables S1–S10 and Figures S1–S7.

FT-IR spectra of phenanthroline-based vanadium(IV) complexes exhibit a high-intensity band around 1675 cm^–1^ due to the antisymmetric stretching vibrational mode ν_as_(−COO). Compared to the free dipicolinic acid (1704 cm^–1^), it is shifted for 25–30 cm^–1^ to lower frequency values due to the coordination of the dipicolinate ion via its carboxylate groups (Figure S8). The band associated with the symmetric stretching vibrational mode ν_s_(−COO) appears at ∼1325 cm^–1^. The difference between ν_as_(−COO) and ν_s_(−COO) is ∼350 cm^–1^, supporting monodentate coordination of the carboxylate groups,^61−64^ which is in accordance with the X-ray analysis. The presence of two antisymmetric and two symmetric stretching vibrational modes ν_as_(−COO) and ν_s_(−COO) in FT-IR spectra of **1** and **2**, in similarity to **9** and **10** reported previously (Figure S8b,c,j,k), is attributed to the involvement of carboxylate groups in the formation of hydrogen bonding with solvent molecules, which results in the unsymmetrical coordination of carboxylate oxygen atoms to the metal center. The higher-frequency bands of pairs ν_as_(−COO) and ν_s_(−COO) correspond to the nonhydrogen carboxylate group and fall in the range observed for phenanthroline-based vanadium(IV) complexes. The band associated with the vanadyl group of the investigated V(IV) complexes appears in the range of 960–986 cm^–1^.

The oxidation state of vanadium in system [VO(dipic)(N^∩^N)] was confirmed by magnetic measurements (Table S11) and EPR spectra (Figures S9 and S10). The values of magnetic moments at room temperature 1.76 μ_B_ for **1**, 1.78 μ_B_ for **2**, 1.77 μ_B_ for **3**, 1.75 μ_B_ for **4**, 1.79 μ_B_ for **5**, 1.80 μ_B_ for **6**, and 1.76 μ_B_ for **7** are similar to those calculated for V(IV) ions (*S* = 1/2) without any exchange interactions and *g* values determined from EPR spectra (Table S11). The X polycrystalline EPR spectra of the magnetically concentrated samples were recorded at room and liquid-nitrogen temperature. There is no change in the line shape, line width, and resolution spin Hamiltonian parameters as a function of temperature. These spectra, typical for the monomeric vanadium(IV) center with *S* = 1/2, exhibit broad anisotropic lines with weak resolution of the signals due to resonance transitions at magnetic fields corresponding to the *g_z_* tensor component. The best fits of the experimental and simulated spectra for all complexes were obtained using average *g* parameters for 1.993 (**1**), 1.979 (**2**), 1.986 (**3**), 1.990 (**4**), 1.983 (**5**), 1.987 (**6**), and 1.984 (**7**) (Figure S9).

EPR frozen solution spectra of oxovanadium(IV) complexes in DMSO (Figure S10) show the eight lines of hyperfine splitting of parallel and perpendicular orientation, proving the interaction of *S* = 1/2 with the nucleus spin of one vanadium and hence the formation of mononuclear compounds. The splitting observed at low fields signals and anisotropy of *g* parameter, observed along the *z-*axis can indicate an additional interaction of oxo ligands with the solvent. The spectra of these mononuclear compounds may be simulated using the spin Hamiltonian parameters *g_x_* = *g_y_* =1.997, *g_z_* = 1.962, *A_x_* = *A_y_* = 65.4 G, *A_z_* = 165 G for **1**; *g_x_* = *g_y_* =1.979, *g_z_* = 1.958, *A_x_* = *A_y_* = 75.3 G, *A_z_* = 186 G for **2**; *g_x_* = *g_y_* =1.981, *g_z_* = 1.942, *A_x_* = *A_y_* = 65.4 G, *A_z_* = 180 G for **3**; *g_x_* = *g_y_* =1.984, *g_z_* = 1.946, *A_x_* = *A_y_* = 68.4 G, *A_z_* = 169 G for **4**; *g_x_* = *g_y_* =1.977, *g_z_* = 1.958, *A_x_* = *A_y_* = 62 G, *A_z_* = 1688.5 G for **5**; *g_x_* = *g_y_* =1.984, *g_z_* = 1.956, *A_x_* = *A_y_* = 71.5 G, *A_z_* = 179 G for **6**; and *g_x_* = *g_y_* =1.979, *g_z_* = 1.950, *A_x_* = *A_y_* = 75, 2 G, *A_z_* = 165 G for **7**, respectively, which are typical for oxidovanadium(IV) compounds with an analogous N_2_O_2_ donor set of the ligands in the vanadium plane^65−67^ and in agreement with the molecular structure determined by X-ray studies.

UV–vis (ultraviolet–visible) spectra of dipicolinate oxovanadium(IV) systems in DMSO exhibit intense high-energy absorption bands corresponding to π → π* transitions localized on the dipic and diamine ligands, moderate charge-transfer (LMCT) transitions between vanadium(IV) and ligands with maximum in the range of 340–430 nm, and weak absorptions in the visible region of 430–1000 nm due to ligand–field transitions (Figures S11, S12 and Table S12). The V^IV^ has a single d electron that gives rise to the ground-state term ^2^T_2g_ in an octahedral crystal field. In the *C*_4*V*_ site symmetry, ^2^T_2g_ splits into ^2^B_2_ and ^2^E, while ^2^E splits into ^2^B_1_ and ^2^A_1_.^68^ For complexes **1** and **2** in DMSO, in similarity to **9** and **10** reported previously, a very broad and asymmetrical band in the range of 520–1000 nm engulfs all three d–d transitions. Phenanthroline-based vanadium(IV) complexes (**3**–**8**) show two separate bands assigned to d–d transitions.

No noticeable changes in the absorbance profiles of oxovanadium(IV) complexes in UV–vis spectra recorded at regular time intervals for 24 h confirm stability of the V(IV) complexes in the DMSO solution (Figure S13).

### Biological Studies

#### Lipophilicity and Cytotoxicity Assays in Tumor and Normal Human 2D Cell Cultures

The lipophilicity of V(IV) complexes was investigated using the classical shake-flash method at room temperature in the two-phase system of immiscible solvents *n*-octanol/PBS buffer.^69,70^ The transfer of V(IV) compounds from the aqueous environment to the organic phase was monitored by UV–vis spectroscopy. The partition coefficients (log *P*), summarized in Table 1, indicate that V(IV) complexes with 1,10-phenanthroline-5,6-dione and imidazole-based ligands are hydrophilic, while others show higher affinity for the organic phase. The highest lipophilic character was found for V(IV) complexes with phen-based ligands. Regarding the substituent introduced into the 4,7-position of the phen core, the value of log *P* increases in the order **8** (phen) < **4** (4,7-Cl_2_-phen) < **5** (4,7-(CH_3_O)_2_-phen) < **6** (4,7-Ph_2_-phen) < **7** (4,7-(phtiaz)_2_-phen).

**Table 1:** Partition Coefficients (log *P*) and Relative IC_50_ Values Obtained for Each of the Vanadium Complexes in HCT116, HCT116-DoxR, A2780, and Fibroblast Cell Lines and Respective SI after 48 h of Exposure^a^

complex	log* P*	cell line	IC_50_ (μM)	SI
**1**	–0.04	HCT116	>50	>0.4
		HCT116-DoxR	>50	>0.4
		A2780	15.0 ± 0.02	1.3
		fibroblasts	20.2 ± 0.04	
**2**	–0.10	HCT116	>50	>0.3
		HCT116-DoxR	31.4 ± 0.03	0.5
		A2780	6.7 ± 0.02	2.3
		fibroblasts	15.7 ± 0.04	
**3**	–0.16	HCT116		
		HCT116-DoxR	1.3 ± 0.07	0.5
		A2780	0.2 ± 0.02	3.5
		fibroblasts	0.7 ± 0.02	
**4**	0.44	HCT116	8.8 ± 0.02	1.0
		HCT116-DoxR	9.0 ± 0.08	1.0
		A2780	2.2 ± 0.02	4.2
		fibroblasts	9.2 ± 0.01	
**5**	0.55	HCT116	1.8 ± 0.04	1.7
		HCT116-DoxR	0.2 ± 0.10	15.5
		A2780	1.3 ± 0.01	2.4
		fibroblasts	3.1 ± 0.07	
**6**	0.57	HCT116	0.9 ± 0.03	4.4
		HCT116-DoxR	0.2 ± 0.05	20.0
		A2780	0.2 ± 0.09	20.0
		fibroblasts	4.0 ± 0.05	
**7**	0.62	HCT116	>50	>0.4
		HCT116-DoxR	>50	>0.4
		A2780	3.1 ± 0.03	7.0
		fibroblasts	21.7 ± 0.08	
**8**	0.40	HCT116	3.7 ± 0.01	2.3
		HCT116-DoxR	1.3 ± 0.04	6.6
		A2780	2.1 ± 0.03	4.1
		fibroblasts	8.6 ± 0.02	
**9**	0.13	HCT116	44.0 ± 0.04	0.4
		HCT116-DoxR	39.2 ± 0.09	0.4
		A2780	4.8 ± 0.02	3.4
		fibroblasts	16.2 ± 0.04	
**10**	–1.28	HCT116	>50	>0.4
		HCT116-DoxR	>50	>0.4
		A2780	14.7 ± 0.02	1.4
		fibroblasts	20.1 ± 0.05	
doxorubicin		HCT116	0.5 ± 0.10	24.2
		HCT116-DoxR	>6^72^	>3.4
		A2780	0.1 ± 0.04	121
		fibroblasts	12.1 ± 0.20	
cisplatin		HCT116	15.6 ± 5.3	0.6
		HCT116-DoxR		
		A2780	1.9 ± 0.20	4.6
		fibroblasts	8.8 ± 2.90	

a IC_50_ values are expressed as the mean ± SEM of at least three biological independent assays.

To assess the cytotoxicity of vanadium complexes in tumor and healthy cells, the MTS assay, a colorimetric method that allows the quantification of viable cells present in solution,^71^ was performed.

**Figure 2:**
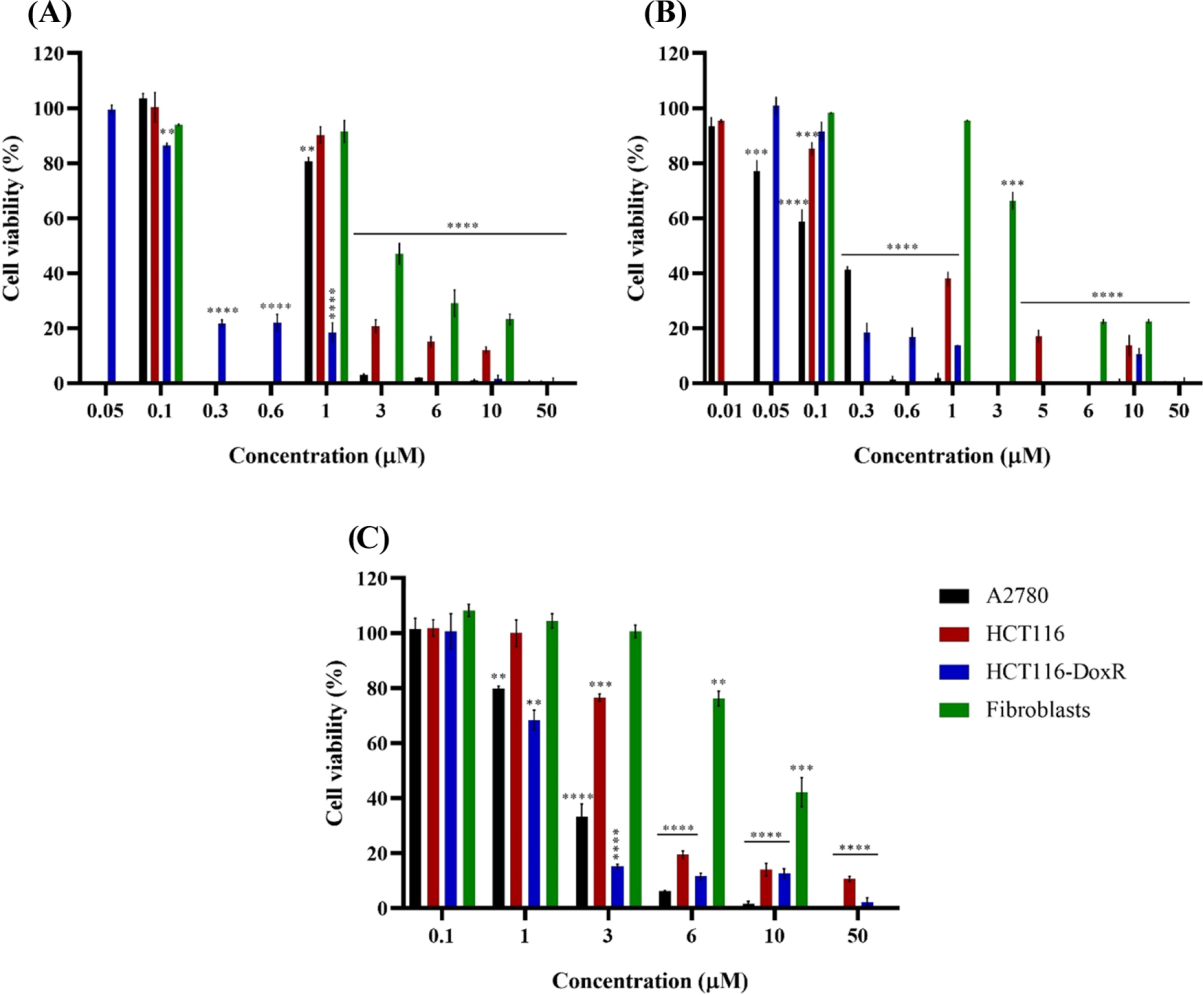
Cell viability of A2780, HCT116, and HCT116-DoxR tumor cell lines and primary normal fibroblasts after exposure to different concentrations of vanadium complexes **5** (A), **6** (B), and **8** (C) for 48 h. 0.1% (v/v) DMSO was used as the vehicle control. Data are expressed as the mean ± SEM of at least two independent assays (**p < 0.01; ***p < 0.001; ****p < 0.0001).

Cell viability was determined after incubation of complexes for 48 h in three tumor cell lines, namely, HCT116 (colorectal carcinoma cell line), HCT116-DoxR (Doxorubicin (Dox)-resistant colorectal carcinoma cell line),^72^ and A2780 (ovarian carcinoma cell line), and in a healthy cell line (normal human primary dermal fibroblasts). A decrease of cell viability with the increase of complexes concentrations might be observed (Figure S14 for complexes **1–4**, **7**, **9**, and **10** and Figure 2 for complexes **5**, **6**, and **8**). For a better comparison among complexes, we have calculated the relative IC_50_ values (the complex concentration that induces a 50% reduction in cell viability) for each complex in the respective cell line (Table 1). Interestingly, when comparing the viability data in colorectal carcinoma cells—sensitive (HCT116) or resistant to Dox (HCT116-DoxR)—after exposure to vanadium complexes, **3**, **5**, **6**, and **8** stand-out as the most promising ones (high cytotoxicity, with an IC_50_ in the order **5** ≅ **6** < **3** ≅ **8**; Table 1 and Figures 2 and S14). Moreover, the lowest relative IC_50_ values (<2 μM; Table 1) were obtained for the HCT116-DoxR cell line. This is highly relevant as a high number of colorectal patients develop resistance to chemotherapeutic agents due to activation of drugs efflux through P-glycoprotein (P-gp) (mechanisms that underline resistance in our HCT116-DoxR cell line).^72^ Complex **4** has a moderate cytotoxicity in both cell lines (IC_50_ ≅ 9 μM; Figure S14 and Table 1). The remaining complexes show higher IC_50_ values, presenting low cytotoxic potential in both cell lines (>30 μM; Table 1).

When analyzing the ovarian carcinoma cell line, A2780, the cytotoxicity order is **3** = **6** > **5** > **8** > 4 > **7** > **9** > **2** > **10** > **1**, and once again the complexes **3**, **5**, **6**, and **8** are the most promising complexes in this tumor cell line (Figure 2, Table 1, and Figure S14). When comparing the results obtained in HCT116-DoxR and A2780 cell lines, Dox-resistant colorectal cancer line is the most sensitive cell line to the action of these complexes, with the exception of complex **3**, which shows a lower IC_50_ value in A2780 (Figure 2, Table 1, and Figure S14). Interestingly, complexes **5** and **6** are more cytotoxic than cisplatin in A2780 and all three complexes (**5**, **6**, and **8**) are more cytotoxic than doxorubicin in HCT116-DoxR (Table 1 and Figure S15).

Since one of the main goals of investigating new complexes is to reduce their side effects in normal tissues, it is important that these vanadium(IV) complexes have significantly higher IC_50_ values in a normal human cell line compared to the respective values in tumor cell lines. Considering this, the relative IC_50_ values of the complexes were determined in healthy cells, namely, primary fibroblasts due to their importance in the tumor microenvironment.^73^ The selectivity index (SI) values (ratio between the IC_50_ in fibroblasts and the IC_50_ in the respective tumor cell line, being a measure of specificity for the tumor line under analysis)^74^ are also reported in Table 1.

When analyzing the SI values in Table 1 for the most interesting complexes, we may observe that complex **3** has a very low SI (0.6 for HCT116-DoxR), meaning that it has a high cytotoxic potential in fibroblasts, and should not be used as a chemotherapeutic agent, particularly in colorectal cancer cells. Complexes **5** and **6**, on the contrary, show the highest SI values (15.5 and 20, respectively) in HCT116-DoxR, meaning that their IC_50_ in fibroblasts is at least 15.5x higher, compared to the respective value in HCT116-DoxR cells (Table 1 and Figure 2). Complex **8** is 6.6 times more cytotoxic in HCT116-DoxR cells compared to fibroblasts.

According to Table 1, when analyzing the SI values for A2780, we may conclude that complexes **4**, **6**, **7**, and **8**, also show a moderate SI (≥4.1).

Thus, from all of the tested complexes, **5**, **6**, and **8** show the greatest therapeutic window and potential in HCT116-DoxR cells. One important consideration is to assess whether the cytotoxicity of the complexes is related to their respective ligands, and in this regard, cell viability assays were also performed for the ligands of each complex in HCT116-DoxR cells and in fibroblasts. The IC_50_ values of the ligands obtained for the HCT116-DoxR tumor cell line and for fibroblasts are shown in Table 2, while the corresponding cell viability data is presented in Figures S16 and S17.

**Table 2:** Relative IC_50_ Values and SI Obtained for the Ligands of Vanadium Complexes in HCT116-DoxR and Fibroblasts Cell Lines after 48 h of Exposure^a^

ligand	IC_50_ HCT116-DoxR (μM)	IC_50_ fibroblasts (μM)	SI
Dipic	>50	>50	
Pyim	>50	>50	
Bipy	>50	>50	
4,7-(phtiaz)_2_-phen	>50	>50	
Pybim	27.1 ± 0.04	≅50	1.8
4,7-Cl_2_-phen	16.4 ± 0.01	17.5 ± 0.07	1.1
Phen	1.6 ± 0.03	>50	>31.3
phendione	0.7 ± 0.01	1.8 ± 0.05	2.6
4,7-Ph_2_-phen	0.3 ± 0.02	4.4 ± 0.13	14.7
4,7-(CH_3_O)_2_-phen	0.3 ± 0.05	10.0 ± 0.01	33.3

a IC_50_ values are expressed as the mean ± SEM of at least three biological independent assays.

Based on Table 2, it is possible to verify that the common ligand, dipic, does not show any cytotoxicity in the HCT116-DoxR cell line or in fibroblasts, indicating that the cytotoxicity of vanadium(IV) complexes [VO(dipic)(N^∩^N)] might come from the diamine ligand.

Considering now the modifications introduced on the diamine core, we can see the following.

(1) The substitution in complex **10** [VO(dipic)(im)_2_]^39^ of the imidazole (im) to a pyim (2-(1*H*-imidazol-2-yl)pyridine)—complex **1**—maintains the absence of cytotoxicity of the original complex **10** in HCT116-DoxR and the moderate cytotoxicity in fibroblasts, while the substitution to a pybim (2-(2-pyridyl)benzimidazole)—complex **2**—leads to a moderate cytotoxicity of complex **2** in HCT116-DoxR cells compared to **10** and **1** due to the intrinsic higher cytotoxicity of the pybim ligand in this cell line (Tables 1 and 2). However, the low SI compared to fibroblasts of **10**, **1**, and **2** indicates that other modifications in the diamine core, namely, of substituents introduced into the 4,7-positions of 1,10-phenanthroline (phen) might be more important for the structure–activity relationships. The phen ligand alone exhibits an IC_50_ > 50 μM (Table 2) in fibroblasts, but when conjugated in complex **8**, its IC_50_ in fibroblasts is 8.6 μM (Table 1).
(2) The introduction of phtiaz groups (with low cytotoxicity) into the 4,7-positions of 1,10-phen of complex **8** ([VO(dipic)(phen)]^57−59^)—which originates complex **7**—reduces the cytotoxicity in HCT116-DoxR (Tables 1 and 2); on the other hand, introduction of 4,7-Cl_2_-phen—originating complex **4**—potentiated cytotoxicity but with no selective index compared to fibroblasts. Finally, the introduction of methoxy (CH_3_O) and phenyl (Ph_2_) groups (with high cytotoxicity in HCT116-DoxR cells—relative IC_50_ of 0.3 μM) led to an increased cytotoxicity of complexes **5** and **6**, respectively, in HCT116-DoxR cells (relative IC_50_ of 0.2 μM) but maintaining SI in high values (Tables 1 and 2). Lastly, ligand phendione, which showed an IC_50_ of 1.8 alone, but when conjugated in complex **3** presented an IC_50_ of 0.7 (Table 1).

Based on these results, the high antiproliferative potential of complexes **3**, **5**, **6**, and **8** is probably mostly due to the structural modifications in the 1,10-phenanthroline core and conjugation of these ligands with V(IV), meaning that the conjugation of vanadium(IV) with the ligands contributes to their greater cytotoxicity. However, SI values for complexes **3** and **8** are much lower than the ones presented by complexes **5** and **6**. As previously mentioned, the results obtained through cell viability assays showed that the HCT116-DoxR cell line was the most sensitive to the action of complexes **5**, **6**, and also **8**. These results are very promising since, as previously stated, this cell line may mimic the resistance acquired by many CRC (colorectal cancer) patients with increased efflux of chemotherapeutic agents via P-gp.^72^ Therefore, these metal complexes may overcome the limitations of other complexes that induce resistance by this mechanism.

The remaining stability and biological studies were performed with these three vanadium complexes (**5**, **6**, and **8**) in the HCT116-DoxR cell line.

#### Complex Stability in Biological Media

Before going more deeply into the biological data regarding these complexes, **5**, **6**, and **8**, their stability in the RPMI culture medium was analyzed at three different time points: 0, 24, and 48 h (Figure S18). The electronic spectra (Figure S18) show an intense band at 230 nm, which may correspond to transitions between π→π* orbitals in the aromatic rings of the 1,10-phenanthroline ligand, present in all three complexes.^75−78^ In the spectrum corresponding to complex **5**, peaks are observed at 260, 300, 310, and 340 nm, with an overall conservation of the maximum peak absorbance over 48 h, meaning that no structural changes of the complex occurred over time (Figure S18). The spectrum of complex **6** shows peaks at 270 and 280, and a small peak at 310 nm, while the spectrum of complex **8** presents peaks at 265 and 330 nm (Figure S18). According to the literature,^75−78^ π → π* transitions of the aromatic rings of the 1,10-phenanthroline ligand can also originate peaks at 260–280 nm. Both complexes (**6** and **8**) showed no spectral changes over time, meaning that no structural changes occurred. However, complex **6** showed a decrease in the maximum absorbance throughout the spectrum (between 0 and 24 h and between 24 and 48 h), revealing a loss of solubility over time (Figure S18). Therefore, to ensure solubility of complexes throughout the assays performed, all complex solutions were freshly prepared prior to performing each assay.

#### Cytotoxicity Assays in HCT116-DoxR Spheroids

To compare the antiproliferative potential of vanadium complexes **5**, **6**, and **8** in 2D and 3D cultures (which better mimic the *in vivo* tumor microenvironment), the MTS assay was also performed on 6- to 8-day-old HCT116-DoxR spheroids after 48 h of exposure.

**Figure 3:**
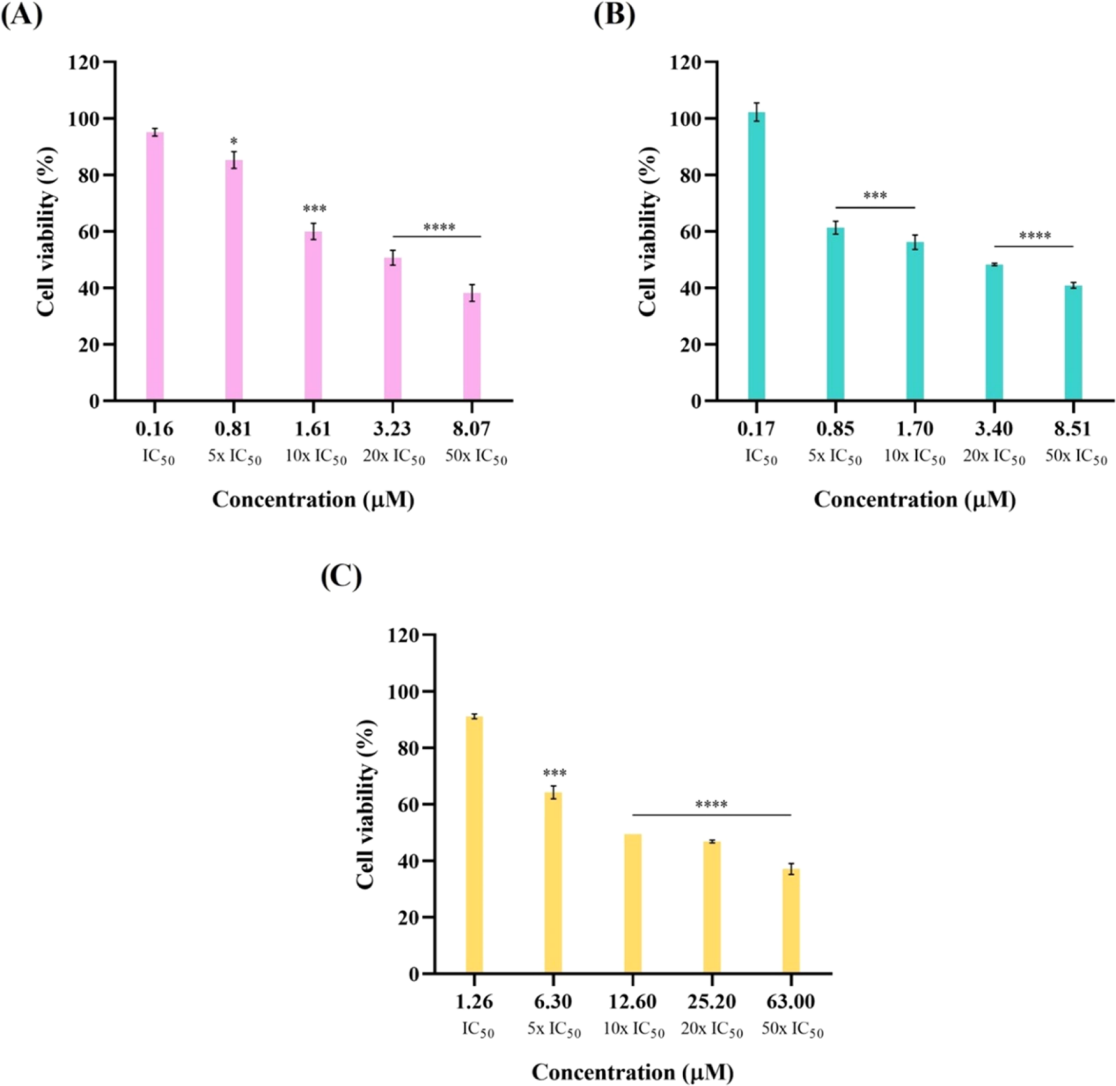
Viability of 8-day-old HCT116-DoxR spheroids after exposure to different concentrations of vanadium complexes **5** (A), **6** (B), and **8** (C) for 48 h. 0.1% (v/v) DMSO was used as the vehicle control. Data are expressed as the mean ± SEM of at least two independent assays (ns = statistically nonsignificant; *p < 0.05; ***p < 0.001; ****p < 0.0001).

Figure 3 shows the cell viability vs. concentration graphics for complexes **5**, **6**, and **8** obtained in 8-day-old HCT116-DoxR spheroids, while Table 3 shows the comparison of IC_50_ values for complexes **5**, **6**, and **8**, obtained for 2D and 3D cultures of HCT116-DoxR cells.

**Table 3:** Comparison of IC_50_ Values Obtained for Complexes **5**, **6**, and **8** in 2D and 3D Cultures of HCT116-DoxR cells^a^

	HCT116-DoxR	
complex	IC_50_ (μM) 2D	IC_50_ (μM) 3D
**5**	0.2 ± 0.10	3.5 ± 0.20
**6**	0.2 ± 0.05	3.1 ± 0.42
**8**	1.3 ± 0.04	11.7 ± 0.62

a IC_50_ values are expressed as the mean ± SEM of at least three biological independent assays.

The results obtained from MTS assays in 3D cultures of HC116-DoxR cells denote a clear increase in the IC_50_ values of vanadium complexes relative to the values obtained for 2D cultures of these same cells. The IC_50_ values in 3D models are approximately 22×, 18×, and 9× higher for complexes **5**, **6**, and **8**, respectively, relative to the values obtained for 2D cultures (Table 3). In 3D structures, cell microenvironment is much more complex, meaning that complexes face several constraints before reaching each individual cell, like diffusion within the 3D structure before cell penetration; this constraint is higher as the spheroid size increases, the reason why spheroids size needs to be controlled (6–8 days).^79,80^ In this regard, by analyzing cytotoxicity at 48 h (as a comparison to 2D), we would expect that much more complex is needed to provide the same biological effect. This has been observed by others.^81,82^ The higher IC_50_ values in 3D models relative to a monolayer will have a greater similarity to the values that these complexes will exhibit *in vivo*. However, the remaining biological assays for determining the mechanisms of action of complexes **5**, **6**, and **8** were performed in 2D models due to their simplicity of methodology.

#### Cell Internalization of the Complexes by Inductively Coupled Plasma-Atomic Emission Spectrometry (ICP-AES)

To evaluate the internalization of vanadium complexes **5**, **6**, and **8** by HCT116-DoxR cells, the ICP-AES technique was performed. This technique allows a quantitative analysis of the metal present in each sample and, therefore, shows if the complexes under study are indeed internalized by the cells.^83^

After 3h of exposure of HCT116-DoxR cells to 10x the IC_50_ values of the vanadium complexes, complexes **5** and **6** show an internalization of ∼100%, while complex **8** shows an internalization of 53% (Table S13). Such results indicate that after 3 h, all three complexes (**5**, **6**, and **8**) were internalized by the HCT116-DoxR cells, with a higher internalization of complexes **5** and **6** compared to complex **8**. These results can be correlated with the viability results, where a lower IC_50_ value (higher cytotoxicity) was observed for complexes **5** and **6** compared to complex **8** (Figure 2 and Table 1). Moreover, due to their fast internalization in cells, their solubility issues for longer incubation periods do not impact their cytotoxicity.

#### Evaluation of Cell Death Mechanisms

After determining that complexes **5**, **6**, and **8** had the greatest therapeutic potential in HCT116-DoxR cells, it was necessary to understand which cell death mechanism was associated with the loss of cell viability observed by exposure to them (Table 2 and Figure S14).

#### Apoptosis

Apoptosis is a regulated cell death process that can be activated if the cell is under adverse conditions.^84−86^ Annexin V is a protein with a high affinity for phosphatidylserine, a phospholipid presented on the inner surface of the lipid bilayer in viable cells. However, when cells undergo apoptosis, this phospholipid is translocated to the outer layer of the lipid bilayer. Thus, the interaction of annexin V with phosphatidylserine is favored and the use of a fluorophore conjugated to annexin V enables the identification of apoptotic cells by flow cytometry.^84,85^ Furthermore, the use of PI (propidium iodide) allows the identification of cells in necrosis, since only cells with a compromised membrane will internalize PI.^84,86^ The Alexa Fluor 488-annexin V/PI assay allows to distinguish cells present in solution into four different stages: viable cells (Alexa Fluor 488^–^; PI^–^), cells in early apoptosis (Alexa Fluor 488^+^; PI^–^), cells in late apoptosis (Alexa Fluor 488^+^; PI^+^), and cells in necrosis (Alexa Fluor 488^–^; PI^+^).^84,86^

**Figure 4:**
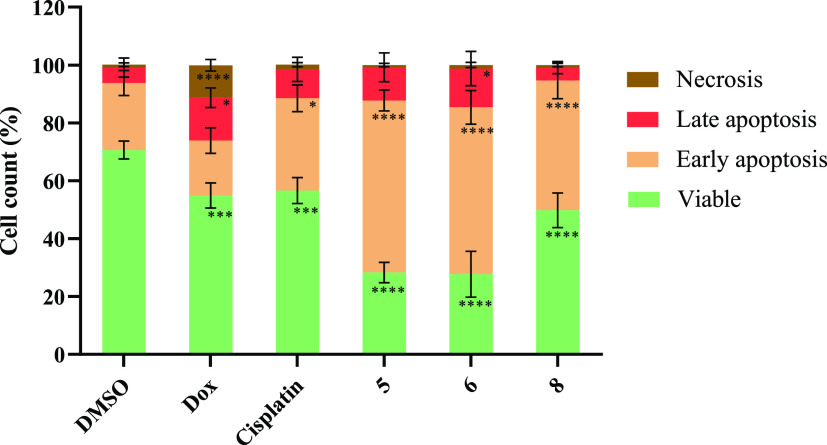
Percentage of viable, apoptotic, and necrotic HCT116-DoxR cells after exposure to the IC_50_ of complexes **5**, **6**, and **8** for 48 h. 0.1% (v/v) DMSO was used as the vehicle control, while 5 μM cisplatin and 6 μM Dox were used as positive controls. Data are expressed as the mean ± SEM of at least two independent biological assays (*p < 0.05; ***p < 0.001; ****p < 0.0001).

Figure 4 and Table 4 show the results obtained in HCT116-DoxR cells exposed to complexes **5**, **6**, and **8** for 48 h. 0.1% DMSO (v/v) was used as the vehicle control and two antitumor compounds, cisplatin (5 μM) and Dox (6 μM), were used as positive controls. The cisplatin concentration was based on assays previously performed in the lab, while the doxorubicin concentration was used according to data observed by Pedrosa et al.^72^

**Table 4:** Percentage of Viable, Apoptotic, and Necrotic HCT116-DoxR Cells after 48 h of Exposure to IC_50_ of Complexes **5**, **6**, and **8** and the Controls Cisplatin, Dox, and DMSO

compound	viable	early apoptosis	late apoptosis	necrosis
DMSO	70.6 ± 3.1	23.2 ± 4.3	5.3 ± 3.3	1.0 ± 0.6
doxorubicin	54.9 ± 4.3	19.0 ± 4.4	14.8 ± 3.4	11.2 ± 2.0
cisplatin	56.6 ± 4.5	31.9 ± 4.6	10.0 ± 4.2	1.6 ± 0.7
**5**	28.3 ± 3.5	59.4 ± 3.6	11.5 ± 5.0	0.8 ± 0.6
**6**	27.7 ± 7.9	57.7 ± 5.8	13.4 ± 5.9	1.2 ± 0.9
**8**	49.8 ± 6.0	44.8 ± 6.2	4.5 ± 2.1	0.9 ± 0.6

The results show that cells exposed to complexes **5** and **6** present a high percentage of apoptosis (∼70%), while cells exposed to complex **8** and cisplatin present about 50 and 40% of apoptosis, respectively. In turn, about 70% of the cells exposed to DMSO alone are viable, as expected, while 28% show cell death by apoptosis and 1% by necrosis (Figure 4 and Table 4). For Dox, about 55% of the cells are viable, while 34% are in apoptosis and 11% in necrosis (Figure 4 and Table 4).

After normalizing the results obtained for the complexes with those obtained for DMSO, complexes **5** and **6** show a 2.5-fold increase percentage of apoptotic cells, while complex **8** shows a 1.7-fold increase of apoptosis. Therefore, these vanadium(IV) complexes are capable of inducing cell death by apoptosis, showing higher apoptosis levels than the two positive controls (Table 4). The activation of the apoptotic cell death by vanadium complexes had already been previously described.^87^ Based on Figure 4 and Table 4, it is also possible to notice that the amount of cells in necrosis was relatively low in all studied conditions, except for cells exposed to doxorubicin (∼11%), a result that correlates with the literature.^88,89^

**Figure 5:**
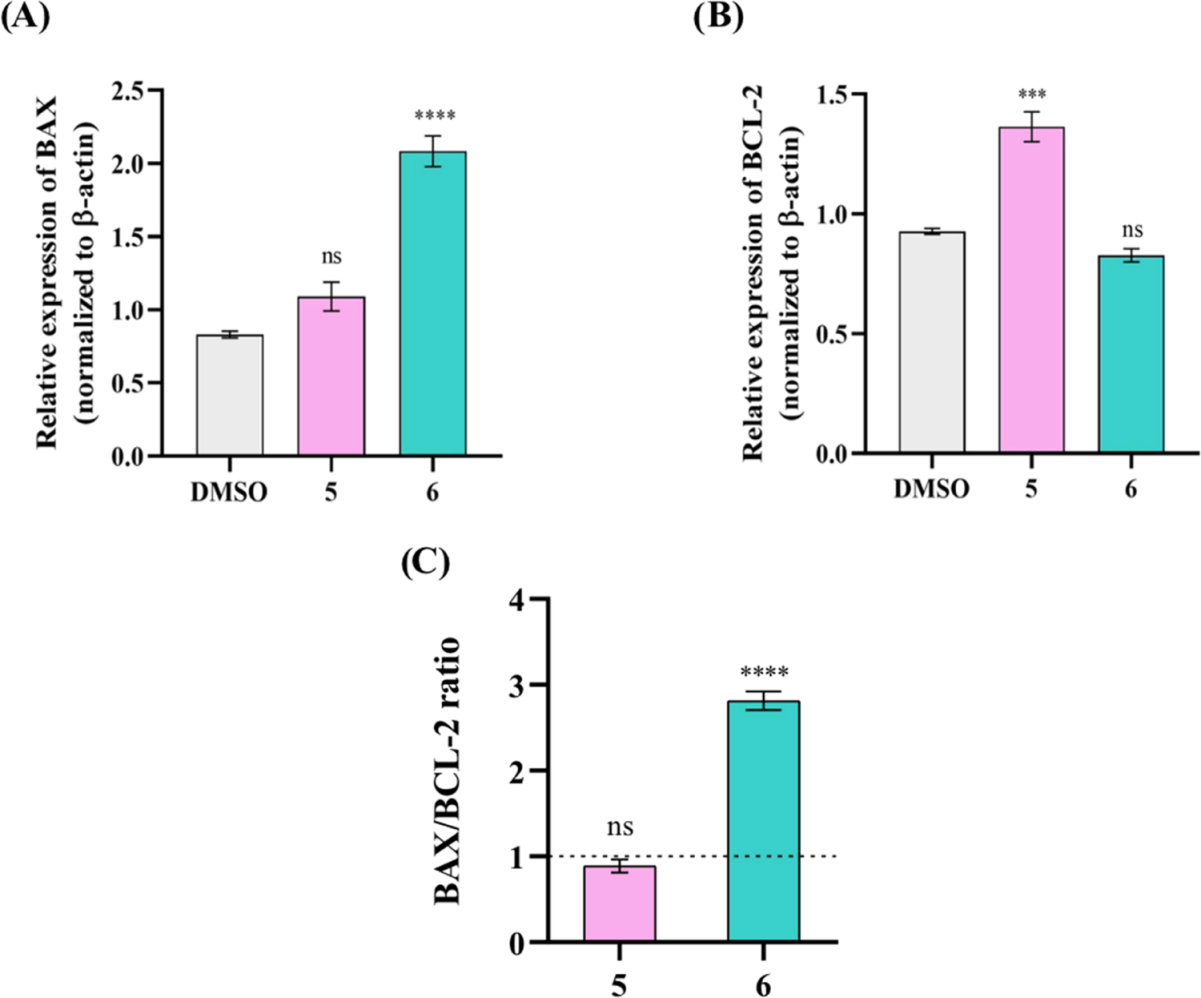
Expression of BAX and BCL-2 proteins in HCT116-DoxR cells after exposure to IC_50_ of complexes **5** and **6** or 0.1% DMSO for 48 h. (A) Relative expression levels of BCL-2. The expression levels of the BCL-2 protein were normalized to a control with β-actin. (B) Relative expression levels of BAX. The expression levels of BAX protein were normalized to a control with β-actin. (C) BAX/BCL-2 ratio. Results were normalized to the control of DMSO after an initial normalization with β-actin. The DMSO value of 1 is represented with a dotted line. Data are expressed as the mean ± SEM (ns = statistically nonsignificant; ***p < 0.001; ****p < 0.0001).

To evaluate if complexes **5** and **6** (complexes with the higher SI in HCT116-DoxR cells) could induce apoptosis by the intrinsic pathway, BAX and BCL-2 protein expression levels were analyzed by Western Blot in HCT116-DoxR cells after 48 h of exposure (Figure 5). When an increase in BAX protein expression occurs compared to BCL-2 protein expression, cell death by apoptosis is promoted. Conversely, an increased expression of BCL-2 compared to BAX protein levels results in cell survival. Thus, the balance of these two proteins is an important factor to determine cell fate.^84,90,91^ The Western Blot bands used for the quantification of proteins BAX, BCL-2, and cleaved PARP1 in HCT116-DoxR cells after their exposure to complexes **5** and **6** are present in Figure S19.

As observed in Figure 5, an increased expression of BAX and BCL-2 proteins is observed upon exposure of HCT116-DoxR cells to complex **5**, compared to the levels of these proteins shown by the control. However, although there was an increase in the expression of both proteins in the cells exposed to complex **5**, it should be noted that the expression level of the BCL-2 protein is higher than that of the BAX protein. On the other hand, cells exposed to complex **6** present an increase in the BAX protein expression and a decrease in the BCL-2 protein expression compared to the expression levels of these proteins observed in the DMSO control (Figure 5). Observing the BAX/BCL-2 ratios present in Figure 5C, complex **6** exhibits a ratio greater than 1, with an ∼3-fold increase over the control, in contrary to complex **5**, which exhibits a BAX/BCL-2 ratio close to 1. These results suggest that complex **6** appears to induce activation of the intrinsic apoptotic pathway in HCT116-DoxR cells, whereas complex **5** does not appear to induce apoptosis via the intrinsic pathway, leading to the possibility that apoptosis might be induced via the extrinsic apoptotic pathway.

**Figure 6:**
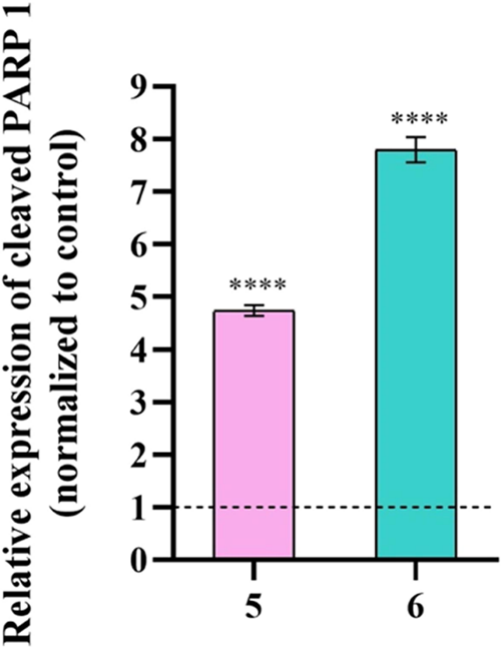
Expression of cleaved PARP1 in HCT116-DoxR cells after exposure to IC_50_ of complexes **5** and **6** or 0.1% DMSO for 48 h. Relative expression levels of cleaved PARP1. The expression levels of cleaved PARP1 were normalized to the control (0.1% DMSO) after an initial normalization with β-actin. The DMSO value of 1 is represented with a dotted line. Data are expressed as the mean ± SEM (****p < 0.0001).

To further confirm that caspases were the effectors of apoptosis, the expression level of the cleaved PARP1, a protein that participates in DNA repair and is cleaved by caspases during the process of cell death by apoptosis,^92,93^ was also analyzed in HCT116-DoxR cells exposed to complexes **5** and **6** and to the 0.1% DMSO control (Figure 6).

The results presented in Figure 6 revealed a 5-fold and an 8-fold increase in the expression levels of cleaved PARP1 in HCT116-DoxR cells exposed to complexes **5** and **6**, respectively, compared to the 0.1% DMSO control. These data confirm the results observed in Figure 4 and the induction of apoptosis with the activation of the effector caspases.

Furthermore, to further confirm that complex **6** acts through the intrinsic apoptosis pathway, contrary to complex **5**, the effect of these complexes in destabilizing the mitochondrial membrane potential (ΔΨ_m_) was analyzed.

#### Alteration of the Mitochondrial Membrane Potential (Δ**Ψ**_m_)

The ΔΨ_m_ is often used as an indicator of the functional state of cells^94^ since mitochondria plays an important intermediary role in cell death by apoptosis.^95^

In some cases of cell exposure to cytotoxic complexes, an increase of the mitochondrial permeability may occur through the disruption of macromolecules located in the mitochondria, and factors such as Cytochrome C may be released into the cytosol, resulting in the induction of apoptosis. In these cases, there is a loss of ΔΨ_m_. Thus, the study of cells’ ΔΨ_m_ is important to understand the functional state of mitochondria and determine the possibility of cell death by the intrinsic apoptosis pathway.^94,96^

**Figure 7:**
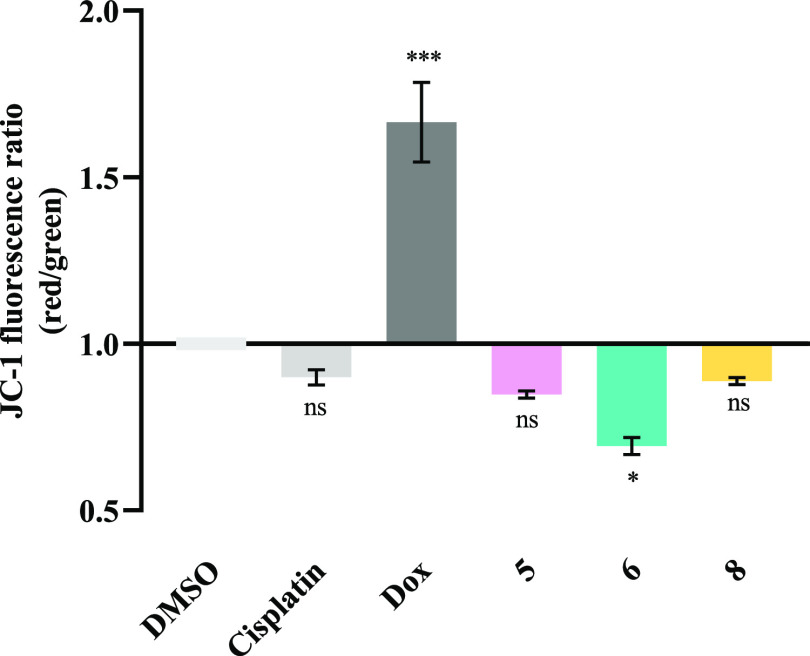
Mitochondrial membrane potential of HCT116-DoxR cells exposed to complexes **5**, **6**, and **8**. Fluorescence ratio of HCT116-DoxR cells labeled with JC-1 after 48 h of exposure to IC_50_ of vanadium complexes. 0.1% (v/v) DMSO was used as the vehicle control, while 5 μM cisplatin and 6 μM Dox were used as positive controls. The results were normalized to the control of DMSO. Data are expressed as the mean ± SEM of at least two independent biological assays (ns = statistically nonsignificant; *p < 0.05; ***p < 0.001).

The use of the fluorescent probe JC-1 (5,5,6,6′-tetrachloro-1,1′,3,3′ tetraethylbenzimidazolcarbocyanine iodide) allows the evaluation of ΔΨ_m_, as the aggregation of the probe changes whether the mitochondrial membrane is hyperpolarized or depolarized.^95,96^ In healthy cells with high mitochondrial potential, JC-1 enters and accumulates inside mitochondria, where it forms complexes known as J-aggregates, emitting red fluorescence. However, in cells with a loss of ΔΨ_m_, JC-1 is unable to remain in the mitochondria and exits to the cytoplasm, accumulating in its monomeric form and emitting green fluorescence. Thus, the ratio of the red/green fluorescence intensities can be used to indicate polarization (ratio > 1) or depolarization (ratio < 1) of the mitochondrial membrane (Figure 7).^94,95^

The results in Figure 7 show an increase in the red/green ratio for Dox compared to control that may indicate an hyperpolarization of the mitochondrial membrane. On the other hand, for cisplatin, no statistically significant decrease was observed (Figure 7). The results show that only the mitochondrial membrane depolarization caused by complex **6** is statistically significant compared to the control, with this complex showing a red/green fluorescence intensity ratio of ∼0.69 (Figure 7). Thus, as expected, after cell exposure to complex **6**, a loss of mitochondrial membrane potential and permeability occurs, resulting in the release of proteins, such as Cytochrome C, into the cytosol, and activation of the intrinsic apoptosis pathway. These pro-apoptotic proteins will participate in the activation of caspase cascades with subsequent cleavage of target molecules, such as PARP1, which are associated with the process of cell death by apoptosis (Figure 6).^95,97^

On the other hand, the data for complexes **5** and **8** suggest that these compounds do not promote cell death through BAX, as observed in Figure 5 (for complex **5**). As both complexes have shown to induce apoptosis (Figure 4), an alternative extrinsic pathway may be involved in the trigger of apoptosis.^98^ The extrinsic pathway of apoptosis is activated through signals received by death receptors present in the cell membrane that then activate caspase-8 and −10.^97^

#### Caspase-8 Activity

**Figure 8:**
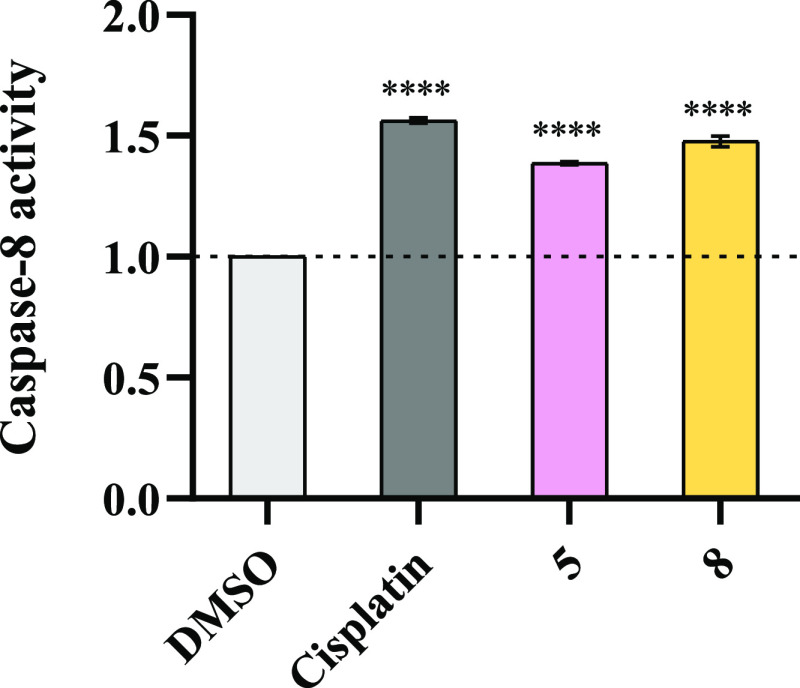
Caspase-8 activity in HCT116-DoxR cells exposed to IC_50_ of complexes **5** and **8** for 48 h. 0.1% (v/v) DMSO was used as the vehicle control and 5 μM cisplatin as the positive control. The results were normalized to the control of DMSO and its value of 1 is represented with a dotted line. Data are expressed as the mean ± SEM of at least two independent biological assays (****p < 0.0001).

Since caspase-8 is an initiator caspase of the extrinsic apoptotic pathway, its activity was evaluated after HCT116-DoxR cells were exposed to complexes **5** and **8** (Figure 8).

The chromogenic substrate IETD-pNA, which results from the conjugation between the peptide IETD (Ile-Glu-Thr-Asp) and the chromophore p-nitroanilide (pNA), is cleaved by caspase-8 and releases pNA, which can be detected by measuring absorbance at 400 nm.^99^ Therefore, if complexes **5** and **8** induce cell death through the extrinsic apoptotic pathway, there is an increase in absorbance at 400 nm compared to the control sample, corresponding to an increase in caspase-8 activity that is translated into the cleavage of the substrate IETD-pNA (Figure 8).

According to the results obtained in Figure 8, when HCT116-DoxR cells are exposed to complexes **5**, **8**, or cisplatin, a higher level of caspase-8 is observed when compared to vehicle control—0.1% DMSO. In agreement with the previous results, these data confirm that both complexes **5** and **8** induce cell death via the extrinsic apoptosis pathway. For complex **6**, caspase-8 activity was not detected.

#### Autophagy

In addition to the analysis of cell death by apoptosis and necrosis already mentioned above, the induction of autophagy by complexes **5**, **6**, and **8** in HCT116-DoxR cells was also analyzed. Autophagy is characterized by the formation of autophagosomes, vesicles with a double membrane, that are able to fuse with lysosomes resulting in cellular material degradation.^98^

**Figure 9:**
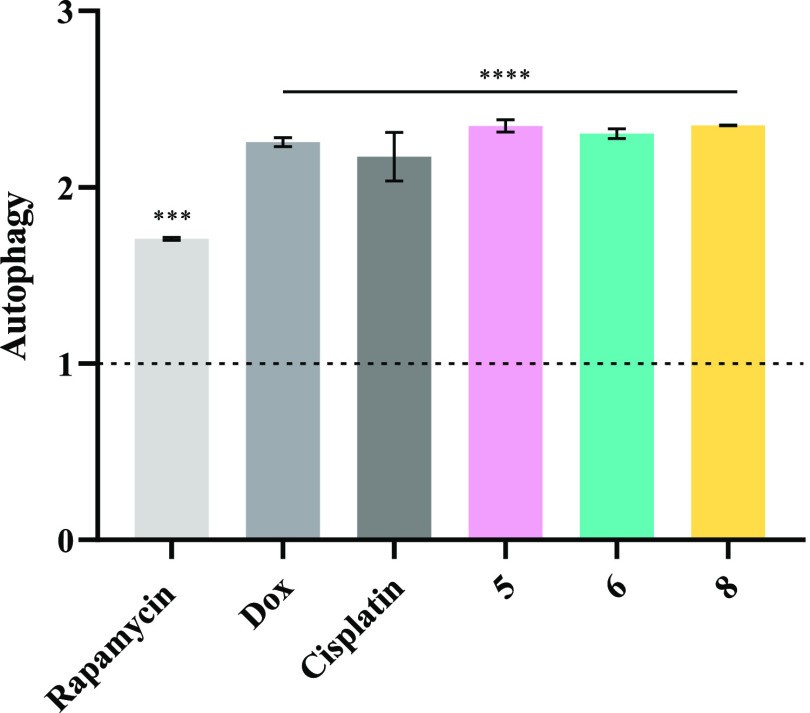
Induction of autophagy in HCT116-DoxR cells after exposure to IC_50_ of complexes **5**, **6**, and **8** for 48 h. 0.1% (v/v) DMSO was used as the vehicle control, while 5 μM cisplatin, 6 μM Dox, and 1500 nM rapamycin were used as positive controls. The results were normalized to the control of DMSO and its value of 1 is represented with a dotted line. Data are expressed as the mean ± SEM of at least two independent biological assays (***p < 0.001; ****p < 0.0001).

To confirm the induction of autophagy in HCT116-DoxR cells after exposure to vanadium complexes, a fluorescent probe (Green Detection Reagent) with the ability to stain autophagic vacuoles was used (Figure 9).^100^ Additionally, a 1500 nM rapamycin solution, an autophagy inducer, was used as a positive control. The rapamycin concentration was optimized for the HCT116-DoxR cell line.

By analyzing the results depicted in Figure 9, complexes **5**, **6**, **8**, doxorubicin, and cisplatin were able to induce the autophagic process in HCT116-DoxR cells, as they revealed an ∼2.3-fold increase of autophagic vesicles over the control. These results indicate that these three vanadium complexes can activate cell death mechanisms both through the activation of apoptosis and autophagy in HCT116-DoxR cells, as already described for other vanadium complexes.^101^

#### Production of Reactive Oxygen Species (ROS)

When cells are exposed to metal complexes, oxidative stress might be induced (with the increase in ROS levels), that consequently can lead to the disruption of cell function and the activation of programmed cell death processes, such as autophagy and apoptosis.^97,102^

**Figure 10:**
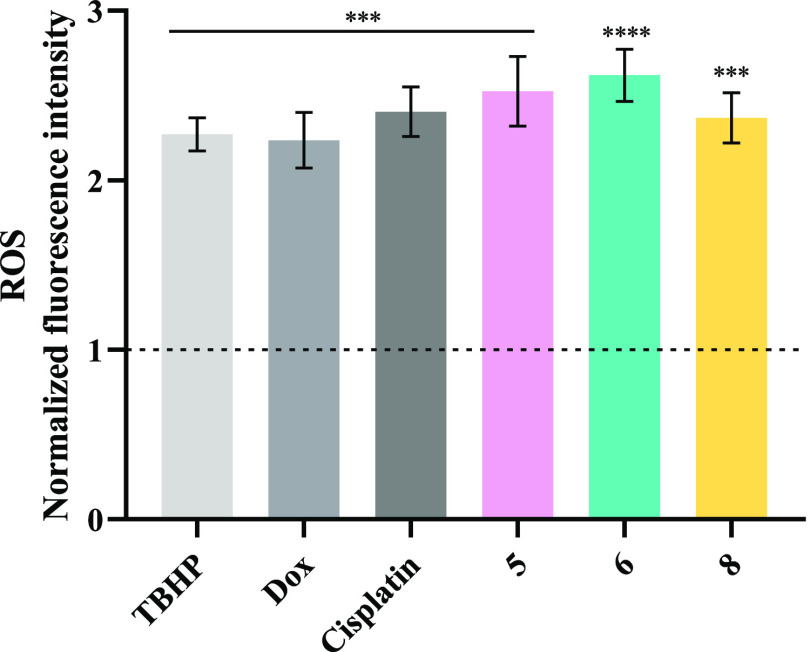
ROS produced by HCT116-DoxR cells after 48 h of exposure to IC_50_ of complexes **5**, **6**, and **8**. 0.1% (v/v). DMSO was used as the vehicle control and 42 μM TBHP (*tert*-butyl hydroperoxide), 5 μM cisplatin, and 6 μM Dox as positive controls. The results were normalized to the control of DMSO and its value of 1 is represented with a dotted line. Data are expressed as the mean ± SEM of at least two independent biological assays (***p < 0.001; ****p < 0.0001).

Thus, the level of ROS present in HCT116-DoxR cells after their exposure to vanadium complexes was quantified (Figure 10). The probe 2′,7′-dichlorohydrofluorescein diacetate (H_2_DCF-DA), which diffuses into cells and is deacetylated by cellular esterases resulting in a nonfluorescent compound, was used. Inside the cell, this molecule is oxidized by ROS and then originates the fluorescent molecule 2′,7′-dichlorofluorescein (DCF) that can be detected by flow cytometry.^103^

A solution of 42 μM TBHP (*tert*-butyl hydroperoxide) was used as a positive control, which corresponds to its IC_50_ value for the HCT116-DoxR tumor cell line, already previously determined (Figure S20).

As observed in Figure 10, the three vanadium complexes **5**, **6**, and **8** induced ROS production in HCT116-DoxR cells, respectively, showing a 2.5-, 2.6-, and 2.4-fold increase over the DMSO control. Interestingly, cancer cells exposed to the three vanadium complexes presented a higher production of ROS relative to the positive control, TBHP, or relative to the antitumor drugs doxorubicin and cisplatin. The high production of ROS by the HCT116-DoxR cells after their exposure to complexes **5**, **6**, and **8** may lead to the activation of cell death by apoptosis and/or autophagy.^98,102,104^ The induction of oxidative stress had already been observed for other vanadium complexes.^87,101^

#### Cell Cycle Progression Analysis

The cytotoxicity of metal complexes can be associated with DNA damage, which can trigger cell cycle checkpoints and result in its arrest. Eventually, if the damage is not repaired, cell death can occur.^105,106^

**Figure 11:**
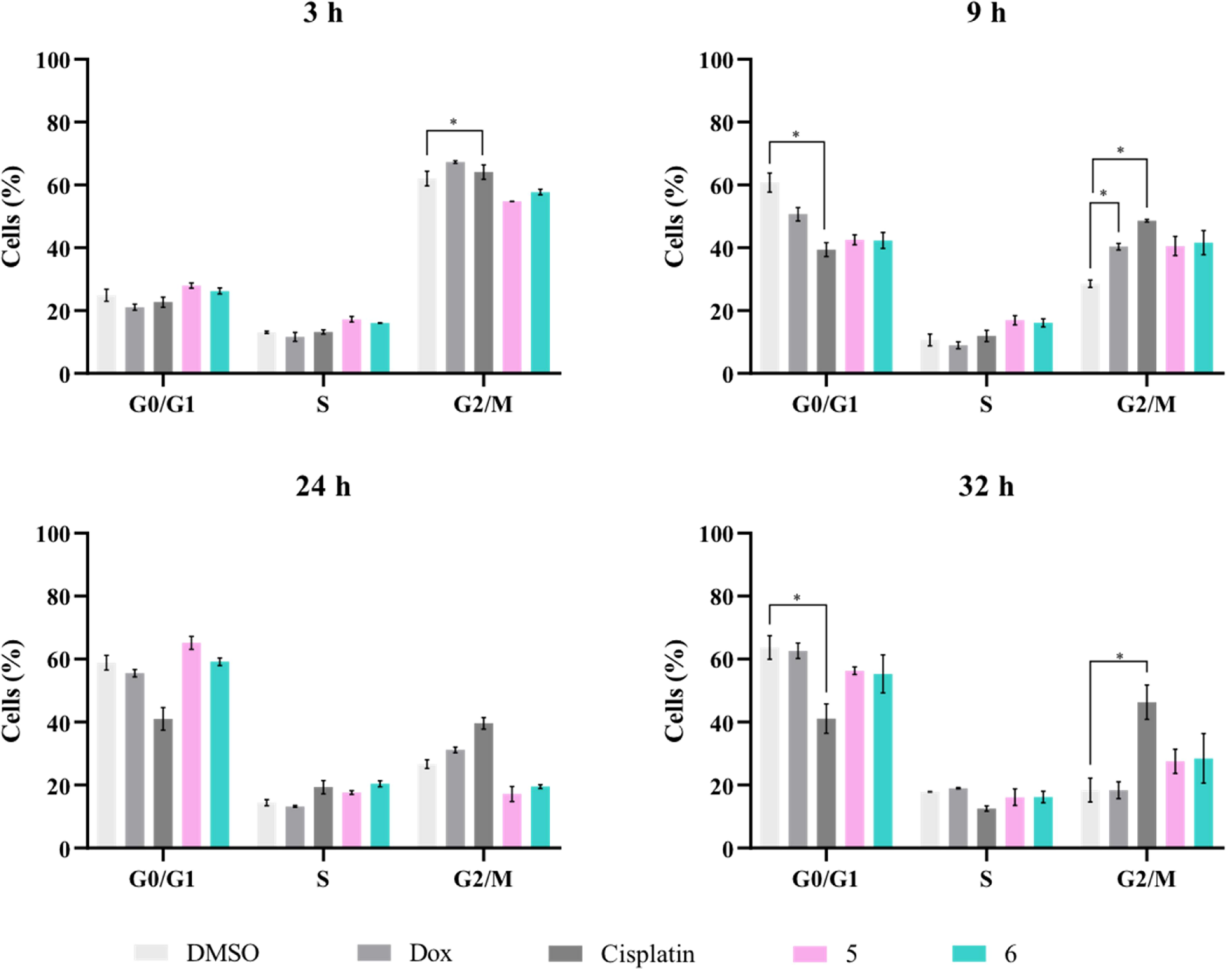
Cell cycle progression in HCT116-DoxR cells after 3, 9, 24, and 32 h of exposure to IC_50_ of complexes **5** and **6**, 5 μM cisplatin, and 6 μM Dox. 0.1% (v/v) DMSO was used as the control vehicle. Data are expressed as the mean ± SEM of at least two independent biological assays (*p < 0.05).

Thus, to verify the cytostatic potential of vanadium complexes, their interference with cell cycle progression was evaluated. The DNA content in each phase of the cell cycle (G0/G1, S, and G2/M) was evaluated by flow cytometry, by using propidium iodide (PI), a fluorescent marker that can intercalate with DNA. Thus, as the amount of DNA duplicates between the G1 and G2 phases, the intensity of fluorescence emitted by the PI will also duplicate.^107,108^ A thymidine solution was used to block the cells in the S phase of the cell cycle. Therefore, a thymidine double block was performed to ensure that all cells were in the same cell cycle phase prior to their exposure to the metal complexes.^107,108^ The effects of complexes on the cell cycle progression were studied 3, 9, 24, and 32 h after cell exposure to IC_50_ of complexes **5** and **6** (Figure 11).

The results show that only cisplatin had a cytostatic effect on HCT116-DoxR cells. This is confirmed by the % of cells present in G0/G1 and G2/M phases in the control sample (DMSO) versus the sample containing cisplatin at 9, 24, and 32 h of the assay.

Although it was expected that Dox would cause a cell cycle arrest in the G2/M phase,^109^ this did not occur after the exposure of HCT116-DoxR cells to 6 μM Dox. These results may be a consequence of Dox resistance in the cell line used, so the concentration of Dox used may not be sufficient to interfere with cell cycle progression.

On the other hand, no cell cycle arrest seems to have occurred in cells exposed to complexes **5** and **6** at their IC_50_ concentration, meaning that these complexes are not cytostatic at these concentrations. This result is not in line with other reports in the literature, where^18,110^ a DNA interaction ability has been determined for phenanthroline vanadium complexes and an induction of a G2/M cell cycle arrest; nevertheless, the substituted ligands were different compared to the present study.

#### BSA (Bovine Serum Albumin) Binding Studies

The high abundance of the HSA (human serum albumin) protein in blood plasma as well as its affinity for various ligands, drugs, and metabolites make it a protein that is often used as a model protein to study drug–protein interactions.^111,112^ This interaction between proteins and drugs influences their pharmacological activity, affecting their absorption, transport, and distribution in the body.^112,113^

In this study, we used the BSA protein that has a 76% similar structure to HSA and is more easily accessible.^112^ BSA is a 66.4 kDa protein composed of 583 amino acid residues divided within three homologous helical domains, namely, I (1–195), II (196–383), and III (384–583), in which each domain is subdivided into two subdomains (A and B).^112,114^ This protein presents two tryptophan residues that possess intrinsic fluorescence.^112,114^

Therefore, we characterized the interaction between the vanadium complexes **5**, **6**, and **8** and BSA *in vitro*, via UV–vis and fluorescence spectroscopies.

**Figure 12:**
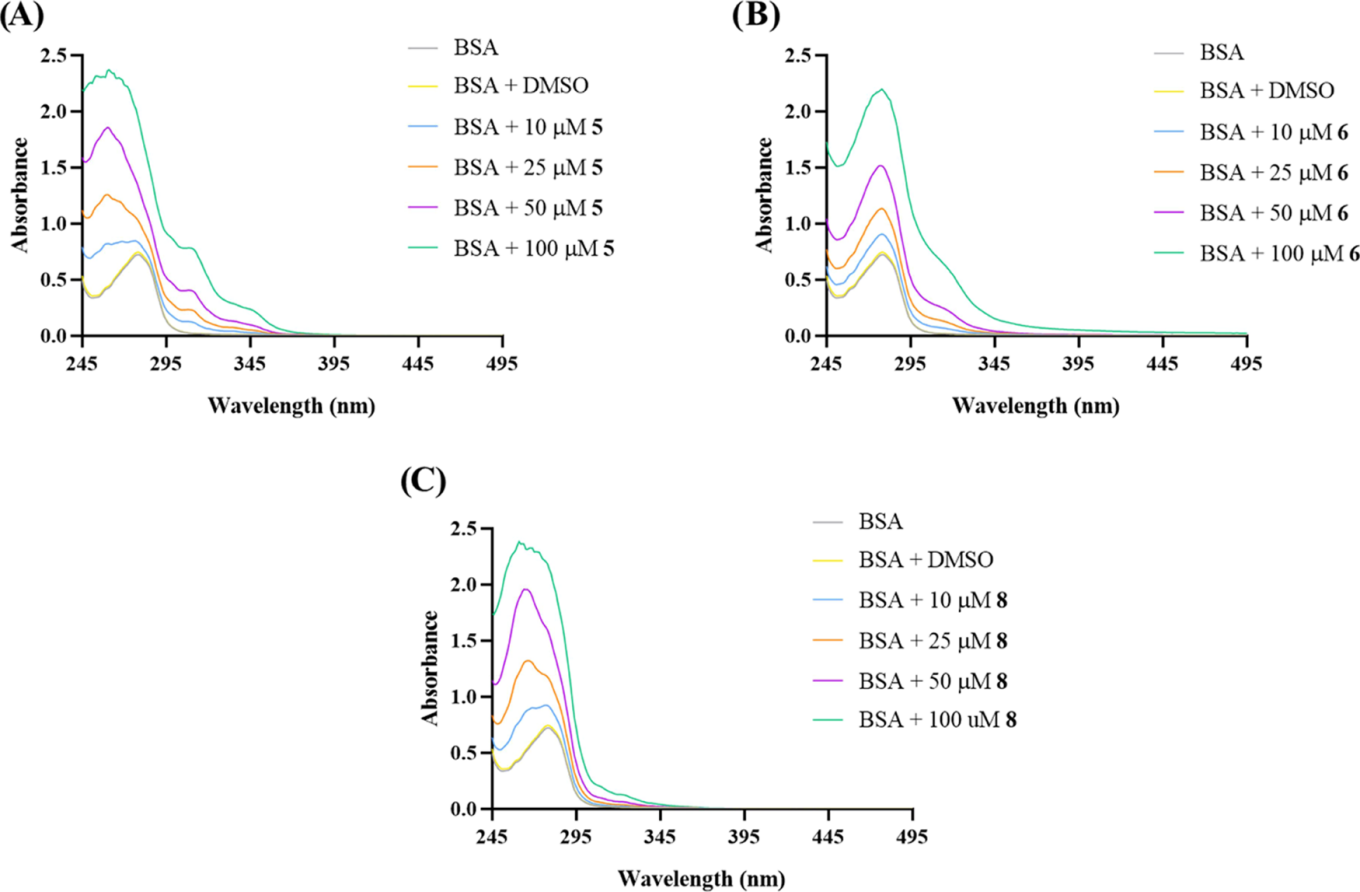
UV–vis spectra of BSA in the absence and presence of DMSO or increasing concentrations of complexes **5** (A), **6** (B), and **8** (C) after exposure for 24 h.

Figure 12 presents the UV–vis spectra obtained for BSA and for BSA in the presence of increasing concentrations of complexes **5 (A)**, **6 (B)**, and **8 (C)** or DMSO, while Figure S21 presents the UV–vis spectra for complexes **5**, **6**, and **8** in increasing concentrations.

As shown in Figure 12, BSA presents an absorption peak at 280 nm that reflects the absorbance from its aromatic amino acids (tryptophan, tyrosine, and phenylalanine).^112,114^ Complex **5** exhibited peaks at 265, 300, 315, and 350 nm, while complex **6** exhibited a peak at 275 nm and complex **8** presented a peak at 265 nm. It is possible to observe that the addition of increasing concentrations of complex **5** to BSA resulted in the appearance of new peaks with a higher maximum absorbance in the spectrum at about 263 and 315 nm, which probably indicates that some changes have occurred in the microenvironment around the aromatic amino acids of the BSA protein. The interaction of complex **6** and BSA resulted in the appearance of a small peak at 320 nm, but the peak at 280 nm did not shift. Lastly, adding increasing concentrations of complex **8** to BSA resulted in the gradual disappearance of the peak at 280 nm and the appearance of a peak at 270 nm, meaning that the microenvironment around the aromatic amino acids of the BSA protein has been affected.

These results seem to indicate that there is an interaction between the BSA protein and the vanadium complexes **5**, **6**, and **8**. However, an assay of fluorescence spectroscopy was also performed to evaluate if these interactions are indeed present between the complexes and the BSA protein.

**Figure 13:**
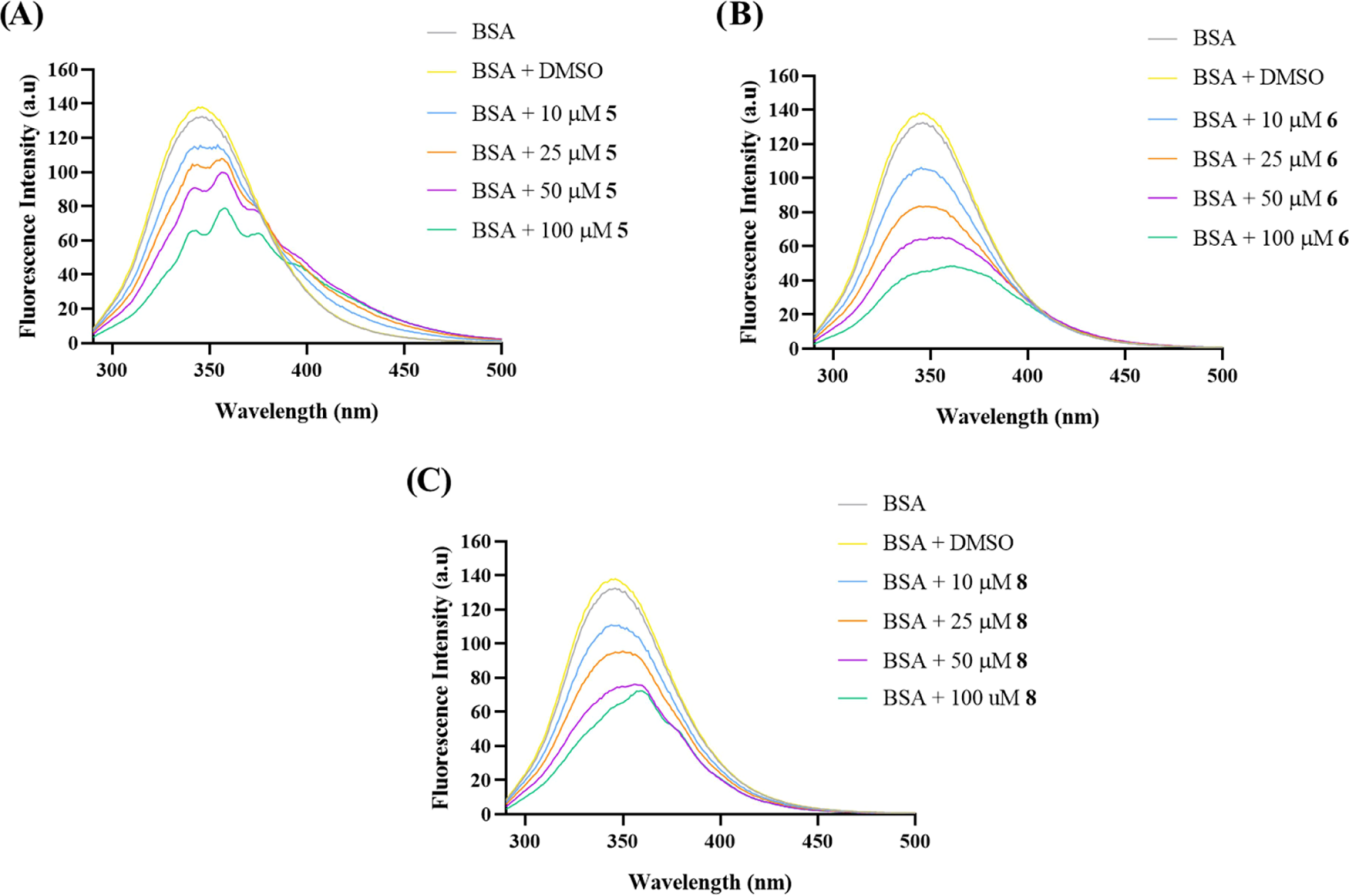
Fluorescence spectra of BSA in the absence and presence of DMSO or increasing concentrations of complexes **5** (A), **6** (B), and **8** (C).

Figure 13 presents the fluorescence spectra obtained for BSA and for BSA in the presence of increasing concentrations of complexes **5** (A), **6** (B), and **8** (C) or DMSO, while Figure S22 presents the fluorescence spectra for complexes **5**, **6**, and **8** alone with increasing concentrations.

According to Figure 13, it is possible to notice that all three complexes **5** (A), **6** (B), and **8** (C) caused a high decrease in BSA fluorescence with its increasing concentrations. Also, spectra (A) and (C) also present some disturbances in the BSA fluorescence when adding higher concentrations of complexes **5** and **8**.

These data suggest that complexes **5**, **6**, and **8** are capable of interacting with the BSA protein. Therefore, the BSA protein might be an example of the capability of these complexes to interact with proteins and, since BSA is an important carrier of ligands and drugs, this can indicate that BSA will be able to carry these compounds throughout the blood plasma until they reach their target.

#### Cell Migration Assay

There is a great need to discover new drugs that have good anti-metastatic potential, since metastasis is the leading cause of cancer death.^115^ Therefore, appropriate *in vitro* assays are needed to identify drugs that do not potentiate cell migration and ideally can inhibit it, as cell migration correlates with the metastatic potential of cancer cells.^116^ One of the most used *in vitro* migration assays is the wound healing assay, which allows the study of *in vitro* cell movement.^116,117^

**Figure 14:**
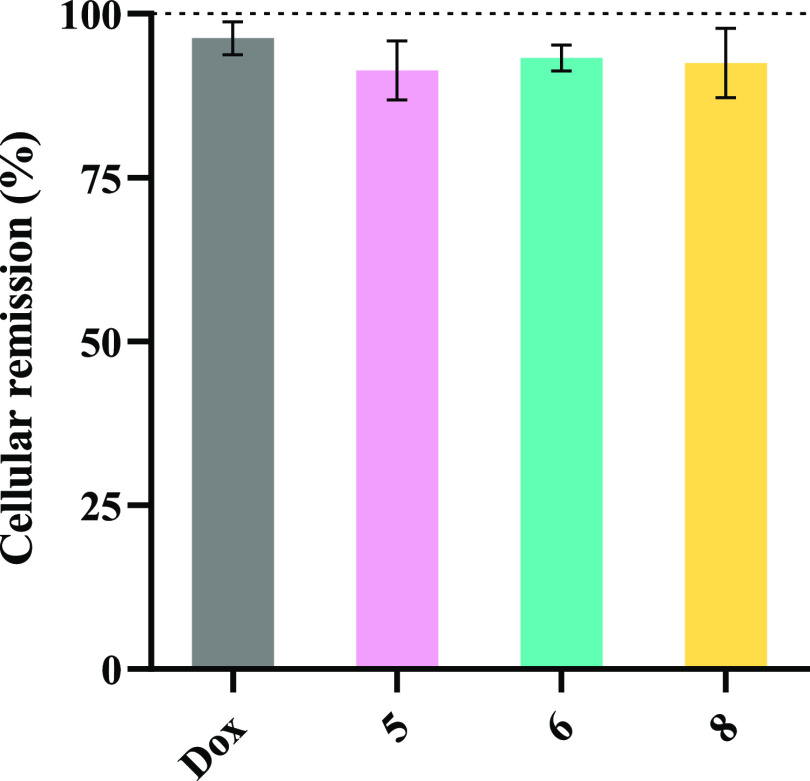
Cell migration of fibroblasts after 24 h of exposure to IC_50_ of complexes **5**, **6**, and **8**, or to 0.4 μM Dox. 0.1% (v/v) DMSO was used as the vehicle control. The results were normalized to the control of DMSO and its value of 100% is represented with a dotted line. Data are expressed as the mean ± SEM of two or more independent trials.

In the wound healing assay, a region without cells is generated, being then exposed to the compounds, and the cell migration is analyzed after 24 h, to determine the cells’ ability to proliferate and refill the gap previously formed (Figure 14).^116^

The results (Figure 14) show that all complexes induced a slightly lower percentage of wound remission than the control, but those values were not statistically significant. Therefore, complexes **5**, **6**, and **8** do not promote cell migration at their IC_50_ concentrations.

#### Ex Ovo Chick Chorioallantoic Membrane (CAM) Assay

The formation of new blood vessels (angiogenesis) around the tumor is crucial for its development, since an increase in angiogenesis enables the nutrition of cancer cells and the invasion of adjacent tissues by the tumor.^117−119^ Therefore, the development of new drugs with anti-angiogenic potential represents a key approach to improve cancer treatment.^116^

**Figure 15:**
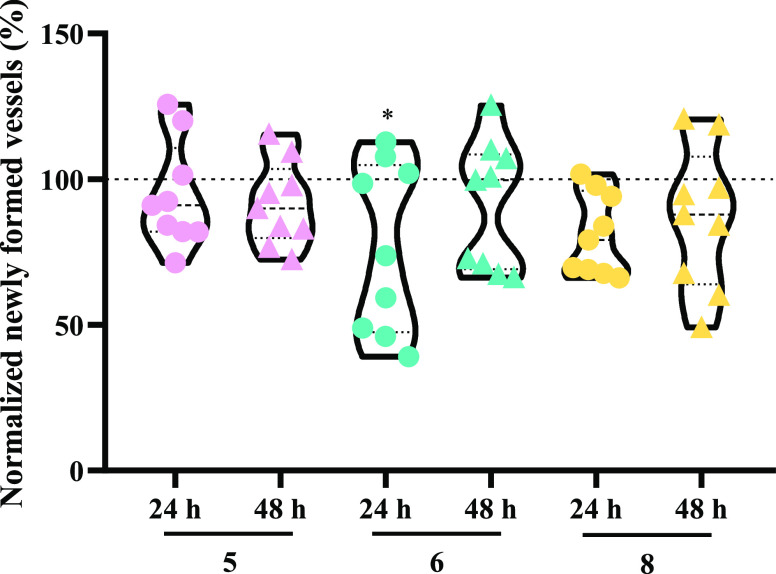
Newly formed blood vessels after exposure of chick embryos to IC_50_ of complexes **5**, **6**, and **8** throughout 48 h. The values were normalized to the number of tertiary veins obtained after exposure to the control (0.1% DMSO) and the number of tertiary veins obtained in the corresponding o-ring at 0 h of incubation in the same embryo. The 100% value refers to the 0.1% (v/v) DMSO sample in PBS 1×. Symbols: dots—24 h, triangles—48 h. *p < 0.05.

The *ex ovo* CAM assay was used to evaluate the role of vanadium complexes in the process of angiogenesis. CAM is a chicken extraembryonic membrane and is often used to study the process of angiogenesis *in vivo* as it has a high density of blood and lymphatic vessels.^120^ This assay consists of analyzing certain vascularized areas of the chorioallantoic membrane of a chicken embryo. Initially, o-rings are placed covering those membrane vascularized areas being then exposed to the complexes. Pictures are then taken at 0, 24, and 48 h after exposure to the complexes and the number of blood vessels present at each hour are counted and compared. The results can be observed in Figure 15.

Based on Figure 15, it is observed that only complex **6** appears to present some anti-angiogenic potential after the first 24 h of exposure. However, at 48 h of exposure of the chicken embryo to complex **6**, the percentage of newly formed blood vessels increases, and the anti-angiogenic potential is no longer present.

The results obtained at 48 h indicate that complexes **5**, **6**, and **8** do not interfere with the formation of new blood vessels, showing no anti-/pro-angiogenic potential at their IC_50_ concentration. Nevertheless, incubation for 24 h with complex **6** appears to be promising in terms of anti-angiogenic effects.

Importantly, a 48 h exposure to the IC_50_ concentration of these three vanadium complexes was found to show no *in vivo* toxicity to embryos, considering their survival throughout the assay period.

## Conclusions

The impact of a series of bidentate N-donor ligands, that is, 2-(1*H*-imidazol-2-yl)pyridine, 2-(2-pyridyl)benzimidazole, 1,10-phenanthroline-5,6-dione, 4,7-dichloro-1,10-phenanthroline, 4,7-dimethoxy-1,10-phenanthroline, 4,7-diphenyl-1,10-phenanthroline, 4,7-phenothiazine-1,10-phenanthroline, 1,10-phenanthroline, and 2,2′-bipyridine, on the anticancer properties of dipicolinate (dipic) vanadium(IV) complexes [VO(dipic)(N^∩^N)] has been investigated.

The antiproliferative effect of vanadium complexes was analyzed in different tumor (A2780, HCT116, and HCT116-DoxR) and normal (primary human dermal fibroblasts) cell lines, revealing a high cytotoxic effect of compounds **5**, **6**, and **8** against the HCT116-DoxR cancer cell line, with their IC_50_ values being 0.2, 0.2, and 1.3 μM, respectively. These complexes showed a higher selectivity against the HCT116-DoxR cell line than against normal primary fibroblasts, as complexes **5**, **6**, and **8** were about 16×, 24×, and 7× more selective for HCT116-DoxR cells than for nontumor cells. Also, the cytotoxicity of complexes **5**, **6**, and **8** seems to be due to the conjugation of vanadium with the diimine core and particularly the substituents introduced into 4,7-positions of 1,10-phenanthroline (phen). This is the first report of vanadium complexes with an impact on multidrug-resistant cells. To compare the antiproliferative potential of complexes **5**, **6**, and **8** in 2D and 3D cell cultures, cytotoxicity assays were also performed on HCT116-DoxR spheroids. These revealed an increase of approximately 10–30× the value of IC_50_ of complexes **5**, **6**, and **8** relatively to 2D monolayers due to the increased spheroid complexity (3D structure) and probable diffusion constrains. This concentration might be important when translating for studies using mice xenografts prompting a more effective response *in vivo*.

The analysis of the internalization of these vanadium complexes by HCT116-DoxR cells showed that complexes **5** and **6** internalize in almost 100% of cells, while complex **8** showed about 53% of internalization. These results were then correlated with cell viability results, where lower internalization would be associated with a higher IC_50_ value (complex **8**) and higher internalization would be associated with a lower IC_50_ value (complexes **5** and **6**).

The cell cycle assay reveals that DNA might not be the main target of vanadium complexes as no complete block of cells’ cycle is observed. On the other hand, BSA studies demonstrated that vanadium complexes can interact with this protein. Albumin is a highly abundant protein in blood, meaning that once in circulation, complexes might bind to HSA and be delivered to tumor cells. Once in tumor cells, these three complexes might trigger ROS production that leads to cell death by apoptosis and autophagy. While complexes **5** and **8** act through the extrinsic apoptosis pathway, complex **6** activates the intrinsic pathway of apoptosis. Furthermore, the three complexes (**5**, **6**, and **8**) do not interfere with the cell migration process and, the *ex ovo* CAM assay, an *in vivo* assay performed in chicken embryos, showed that after 24 h of exposure, only complex **6** might induce a decrease in the percentage of newly formed blood vessels. However, this anti-angiogenic effect was not observed after 48 h of exposure to this complex. Lastly, it was also observed that 48 h of exposure to IC_50_ concentrations of these vanadium complexes does not interfere with the survival of chicken embryos.

Taken this data together, the three vanadium complexes presented a good antiproliferative potential in HCT116-DoxR cells with no *in vivo* toxicity that are of relevance for further preclinical studies using other *in vivo* models.

## Experimental Section

All investigated complexes were obtained in a form of X-ray quality monocrystals, and their molecular structures were confirmed by X-ray. The purity of all compounds is >95% that was confirmed by elemental analysis.

### Materials

2-(1*H*-imidazol-2-yl)pyridine, 2-(2-pyridyl)benzimidazole, 4,7-dichloro-1,10-phenanthroline, 4,7-diphenyl-1,10-phenanthroline, 4,7-dimethoxy-1,10-phenanthroline, 1,10-phenanthroline, 2,2′-bipyridine, and imidazole were commercially available (Sigma-Aldrich), and they were used without further purification. 4,7-Phenothiazine-1,10-phenanthroline,^121^ [VO(dipic)(H_2_O)_2_],^39,56^ [VO(dipic)(phen)],^57−59^ [VO(dipic)(bipy)],^59,60^ and [VO(dipic)(im)_2_]^39^ were prepared according to a procedure described in the literature, and their analytical data provided a good agreement with those reported in refs (39, 56−60, 121).^39,56−60,121^

### Synthesis of [VO(dipic)(N^∩^N)]

The complex [VO(dipic)(H_2_O)_2_] (1 mmol) and appropriate diamine ligand (1 mmol) were dissolved in methanol (30 mL), and the resulting solution was refluxed under argon for 6 h. After cooling to room temperature, the precipitate of [VO(dipic)(N^∩^N)] was collected by filtration and dried in air. Crystals suitable for X-ray analysis were obtained by recrystallization from methanol/chloroform or acetonitrile/chloroform mixtures (1/1 v/v).

#### [VO(dipic)(pyim)] (**1**)

Yield 65%. Anal. calcd for C_15_H_10_N_4_O_5_V (377.21 g/mol): C 47.76, H 2.67, N 14.85%. Found: C 47.48, H 2.92, N 14.48%. IR (KBr, cm^–1^): 3122(m) and 3059(m) [ν(N–H)], 1676(vs) and 1637(vs) [ν_as_(COO)], 1601(m) and 1575(w) [ν(C=N) and ν(C=C)]; 1366(s) and 1340(s) [ν_s_(COO)], 986(s) [ν(V=O)] and 439(m) [ν(V–N)]. UV–vis (DMSO, λ_max_, nm (ε, dm^3^·mol^–1^·cm^–1^)): 873 (27); 634 (15); 298 (14 200); 274 (13 600).

#### [VO(dipic)(pybim)] (**2**)

Yield 60%. Anal. calcd for C_19_H_12_N_4_O_5_V (427.27 g/mol): C 53.41, H 2.83, N 13.11%. Found: C 53.09, H 2.89, N 12.75%. IR (KBr, cm^–1^): 3182(m) and 3076(m) [ν(N–H)], 1670(vs) and 1629(vs) [ν_as_(COO)], 1596(s) [ν(C=N) and ν(C=C)]; 1367(s) and 1345(s) [ν_s_(COO)], 981(s) [ν(V=O)] and 437(m) [ν(V–N)]. UV–vis (DMSO, λ_max_, nm (ε, dm^3^·mol^–1^·cm^–1^)): 892 (32); 639 (18); 323 (13 200); 312 (16 000); 271 (6400).

#### [VO(dipic)(phendione)] (**3**)

Yield 70%. Anal. calcd for C_19_H_9_N_3_O_7_V (442.23 g/mol): C 51.60, H 2.05, N 9.50%. Found: C 51.52, H 2.45, N 9.26%. IR (KBr, cm^–1^): 1688(vs) [νC=O], 1674(vs) [ν_as_(COO)], 1668(sh), and 1575(m) [ν(C=N) and ν(C=C)]; 1327(s) [ν_s_(COO)], 980(s) [ν(V=O)], and 443(m) [ν(V–N)]. UV–vis (DMSO, λ_max_, nm (ε, dm^3^·mol^–1^·cm^–1^)): 828 (29); 619 (18); 447 (170); 338 (1600); 292 (7800); 263 (18 300).

#### [VO(dipic)(4,7-Cl_2_-phen)] (**4**)

Yield 65%. Anal. calcd for C_19_H_9_Cl_2_N_3_O_5_V (481.13 g/mol): C 47.43, H 1.89, N 8.73%. Found: C 47.12, H 2.04, N 8.37%. IR (KBr, cm^–1^): 1678(vs) [ν_as_(COO)], 1616(w), 1566(m), and 1511(m) [ν(C=N) and ν(C=C)]; 1323(s) [ν_s_(COO)], 978(s) [ν(V=O)], and 439(m) [ν(V–N)]. UV–vis (DMSO, λ_max_, nm (ε, dm^3^·mol^–1^·cm^–1^)): 891 (31); 471 (140); 388 (540); 347 (880); 329 (2000); 300 (20 700); 281 (30 500); 271 (27 500).

#### [VO(dipic)(4,7-(CH_3_O)_2_-phen)] (**5**)

Yield 60%. Anal. calcd for C_21_H_15_N_3_O_7_V (472.31 g/mol): C 53.40 H 3.20, N 8.90%. Found: C 53.04, H 2.92, N 8.58%. IR (KBr, cm^–1^): 1674(vs) [ν_as_(COO)], 1580(s), 1528(s), and 1515(sh) [ν(C=N) and ν(C=C)]; 1328(s) and 1305(sh) [ν_s_(COO)], 967(s) [ν(V=O)] and 436(m) [ν(V–N)]. UV–vis (DMSO, λ_max_, nm (ε, dm^3^·mol^–1^·cm^–1^)): 779 (33); 580 (23); 443 (590); 340 (4480); 311 (13 600); 299 (13 800); 271 (28 800).

#### [VO(dipic)(4,7-Ph_2_-phen)] (**6**)

Yield 70%. Anal. calcd for C_31_H_19_N_3_O_5_V (564.43 g/mol): C 65.97, H 3.39, N 7.44%. Found: C 65.66, H 3.02, N 7.41%. IR (KBr, cm^–1^): 1673(vs) [ν_as_(COO)], 1597(w), 1557(m), 1520(m), and 1492(w) [ν(C=N) and ν(C=C)]; 1323(s) [ν_s_(COO)], 967(s) [ν(V=O)] and 440(m) [ν(V–N)]. UV–vis (DMSO, λ_max_, nm (ε, dm^3^·mol^–1^·cm^–1^)): 743 (47); 478 (740); 382 (1600); 289 (29 500); 279 (33 000).

#### [VO(dipic)(4,7-(phtiaz)_2_-phen)] (**7**)

Yield 55%. Anal. calcd for C_43_H_25_N_5_O_5_S_2_V (806.76 g/mol): C 64.02 H 3.12, N 8.68%. Found: C 64.32, H 2.78, N 8.42%. IR (KBr, cm^–1^): 1676(vs) [ν_as_(COO)], 1575(m) and 1517(w) [ν(C=N) and ν(C=C)]; 1325(s) [ν_s_(COO)], 973(s) [ν(V=O)], and 439(m) [ν(V–N)]. UV–vis (DMSO, λ_max_, nm (ε, dm^3^·mol^–1^·cm^–1^)): 880 (22); 737 (19); 467 (450); 325 (9100); 302 (18 100); 284 (26 300); 274 (25 000).

## Supporting Information Available

Experimental data for V(IV) complexes prepared according to literature procedures, experimental methods, HPLC traces, X-ray data, IR spectra of compounds and ligands, EPR spectra, electronic absorption spectra, stability and solubility in DMSO and RPMI, cell viability of A2780, HCT116, HCT116-DoxR, and fibroblast cell lines after exposure to different concentrations of vanadium complexes, ligands, doxorubicin, and cisplatin, ICP-AES data, Western blot bands, viability of HCT116-DoxR cells after exposure to different concentrations of TBHP for 48 h, and UV–vis and fluorescence spectra of complexes **5**, **6**, and **8** (PDF)
Molecular formula strings (CSV)
Crystallographic Information Files of complexes **1**–**7** (CIF)

## Supplementary Material

jm3c00255_si_002.csv

jm3c00255_si_003.cif

## Accession Codes

Crystallographic data for **1–7** were deposited with the Cambridge Crystallographic Data Center. CCDC Numbers: 2233583 (**1**), 2233578 (**2**), 2233582 (**3**), 2233579 (**4**), 2233584 (**5**), 2233580 (**6**), and 2233581 (**7**). Authors will release the atomic coordinates upon article publication. Copies of this information may be obtained free of charge from the Director, CCDC, 12 Union Road, Cambridge CB2 1EZ, U.K. (Fax: +44 1223 336033; Email: deposit@ccdc.cam.ac.uk or www.ccdc.cam.ac.uk).

## Abbreviations Used

ΔΨ_m_: mitochondrial membrane potential
A2780: ovarian carcinoma cell line
bipy: 2,2′-bipyridine
BSA: bovine serum albumin
CAM: chick chorioallantoic membrane
CRC: colorectal cancer
DCF: 2′,7′-dichlorofluorescein
dipic: dipicolinate
DMEM: Dulbecco’s modified Eagle’s medium
Dox: doxorubicin
H_2_DCF-DA: 2′,7′-dichlorohydrofluorescein diacetate
HCT116: colorectal carcinoma cell line
HCT116-DoxR: doxorubicin-resistant colorectal carcinoma cell line
HSA: human serum albumin
ICP-AES: inductively coupled plasma-atomic emission spectrometry
IETD: Ile-Glu-Thr-Asp
im: imidazole
JC-1: 5,5,6,6′-tetrachloro-1,1′,3,3′ tetraethylbenzimidazolcarbocyanine iodide
LMCT: ligand-to-metal charge transfer
P-gp: P-glycoprotein
phen: 1,10-phenanthroline
phendione: 1,10-phenanthroline-5,6-dione
phtiaz: phenothiazine
PI: propidium iodide
pNA: p-nitroanilide
pybim: 2-(2-pyridyl)benzimidazole
pyim: 2-(1*H*-imidazol-2-yl)pyridine
ROS: reactive oxygen species
RPMI: Roswell Park Memorial Institute
SI: selectivity index
TBHP: *tert*-butyl hydroperoxide
UV–vis: ultraviolet–visible
