# Supplementary material for: In Vitro and In Vivo Biological Activities of Dipicolinate Oxovanadium(IV) Complexes
Source: J Med Chem. 2023 Jun 13;66(13):8580–99. doi: 10.1021/acs.jmedchem.3c00255 (PMC10350923; doi:10.1021/acs.jmedchem.3c00255)
Supplement: Supplementary file 1 — jm3c00255_si_001.pdf [file jm3c00255_si_001.pdf]

Supporting Information

In vitro and *in vivo* biological activity of dipicolinate oxovanadium(IV) complexes

Katarzyna Choroba[a,¥], Beatriz Filipe[b,c,¥] Anna Świtlicka[a], Mateusz Penkala[a], Barbara Machura[a,*], Alina Bieńko[d], Sandra Cordeiro[b,c], Pedro V. Baptista[b,c], Alexandra R. Fernandes[b,c,*]

[a] University of Silesia, Institute of Chemistry, Szkolna 9, 40-006 Katowice, Poland. Email: barbara.machura@us.edu.pl
[b] Associate Laboratory i4HB - Institute for Health and Bioeconomy, NOVA School of Science and Technology, NOVA University Lisbon, 2819-516 Caparica, Portugal
[c] UCIBIO – Applied Molecular Biosciences Unit, Department of Life Sciences, NOVA School of Science and Technology, NOVA University Lisbon, 2819-516 Caparica, Portugal. Email: ma.fernandes@fct.unl.pt
[d] University of Wroclaw, Faculty of Chemistry, F. Joliot-Curie 14, 50-383 Wroclaw, Poland

*Corresponding authors: barbara.machura@us.edu.pl and ma.fernandes@fct.unl.pt
¥ Co-first authors (these authors contributed equally to this work)

Table of Contents

**Experimental**		
Experimental data for V(IV) complexes prepared according to literature procedures		page S2
Experimental methods		page S2
**X-ray studies**		
Crystal data and structure refinement for **1**–**7**	Table S1	page S8
Selected bond lengths and angles for complexes **1–7**	Table S2–S8	page S9
Short intra- and intermolecular hydrogen bonds in structures **1**–**7**	Table S9	page S12
Short π•••π interactions in structures **1**–**7**	Table S10	page S13
View of the intermolecular interactions and packing in structures **1–7**	Figure S1–S7	page S13
**IR spectroscopy**		
IR spectra of the V(IV) complexes in comparison to dipicolinic acid and free ligands (a–k)	Figure S8	page S17
**EPR spectroscopy**		
Magnetic moments of **1–7**	Table S11	page S22
X-band EPR spectra of **1–9** at 77 K together with simulated spectra	Figure S9	page S23
EPR frozen solution spectra (at 77 K) of **1–9** in DMSO solvent together with simulated spectra	Figure S10	page S24
**Absorption spectroscopy**		
The absorption maxima and molar extinction coefficients for **1**–**10**	Table S12	page S25
Electronic absorption spectra of **1**–**10** in DMSO and MeOH	Figure S11	page S26
Electronic absorption spectra of **1**–**10** in MeOH in comparison to the spectra of the dipicolinic acid, [VO(dipic)(H_2O)$_2$] and corresponding ligand	Figure S12	page S27
UV-Vis stability spectra in DMSO for compounds **1–10**	Figure S13	page S29
**Biological studies**		
Cell viability of A2780, HCT116, HCT116-DoxR and fibroblasts cell lines after exposure to different concentrations of the vanadium complexes **1**–**4**, **7**, **9** and **10**	Figure S14	page S31
Cell viability of HCT116, A2780 and fibroblasts cells after exposure to different concentrations of doxorubicin and cisplatin for 48 hours	Figure S15	page S32
Cell viability of HCT116-DoxR cells after exposure to different concentrations of the vanadium complexes' ligands for 48 hours	Figure S16	page S33
Cell viability of fibroblasts after exposure to different concentrations of the vanadium complexes' ligands for 48 hours	Figure S17	page S34
Stability and solubility of complexes **5**, **6** and **8** by UV-Vis spectroscopy in RPMI	Figure S18	page S35
Data obtained with the ICP-AES experiment after 3 hours of exposure of HCT116-DoxR cells to 10x IC50 of complexes 5, 6 and 8	Table S13	page S36
Western Blot bands used for the quantification of proteins BAX, BCL-2 and cleaved PARP1 in HCT116-DoxR after their exposure to complexes **5**, **6** or DMSO	Figure S19	page S36

Viability of HCT116-DoxR cells after exposure to TBHP for 48 hours	Figure S20	page S37
UV-Vis spectra of complexes 5, **6** and **8** with its concentrations increasing and of DMSO, used as the control, measured and recorded after 24 h of exposure	Figure S21	page S38
Fluorescence spectra of complexes 5, **6** and **8** with its concentrations increasing, measured and recorded after 24 h of exposure	Figure S22	page S39
References		

Experimental

Experimental data for complexes 8–10

[VO(dipic)(phen)] (**8**): Yield 70%. Anal. Calc. for $C_{19}H_{11}N_3O_5V$ (412.25 g/mol): C 55.36 H 2.69, N 10.19 %. Found: C 54.98, H 2.77, N 9.80 %. IR (KBr, cm^{-1}): 1676(vs) [ν_{as}(COO)], 1598(w) and 1518(m) [ν(C=N) and ν(C=C)]; 1328(s) [ν_s(COO)], 968(s) [ν(V=O)] and 439(m) [ν(V–N)]. UV-Vis (DMSO, λ_{max}, nm (ε, $dm^3{\cdot}mol^{-1}{\cdot}cm^{-1}$)): 875 (21); 744 (20); 456 (220); 362 (490); 292 (7500); 272 (26300)

[VO(dipic)(bipy)] (**9**): Yield 65%. Anal. Calc. for $C_{17}H_{11}N_3O_5V$ (388.23 g/mol): C 52.59 H 2.86, N 10.82 %. Found: C 52.24, H 3.23, N 10.43 %. IR (KBr, cm^{-1}): 1672(vs) and 1652(vs) [ν_{as}(COO)], 1600(s) and 1574(m) [ν(C=N) and ν(C=C)]; 1337(s) and 1313 [ν_s(COO)], 975(s) [ν(V=O)] and 444(m) [ν(V–N)]. UV-Vis (DMSO, λ_{max}, nm (ε, $dm^3{\cdot}mol^{-1}{\cdot}cm^{-1}$)): 888 (10); 641(4); 381 (260); 340 (300); 279 (16000); 263 (11500)

[VO(dipic)(im)$_2$] (**10**): Yield 75%. Anal. Calc. for $C_{13}H_{11}N_5O_5V$ (368.20 g/mol): C 42.41 H 3.01, N 19.02 %. Found: C 42.72, H 3.28, N 19.34 %. IR (KBr, cm^{-1}): %. IR (KBr, cm^{-1}): 3142(m), 2954(m) and 2866(m) [ν(N–H)], 1678(vs) and 1640(vs) [ν_{as}(COO)], 1357(s) and 1335(s) [ν_s(COO)], 960(s) [ν(V=O)] and 442(m) [ν(V–N)]. UV-Vis (DMSO, λ_{max}, nm (ε, $dm^3{\cdot}mol^{-1}{\cdot}cm^{-1}$)): 888 (26); 641(9); 383 (200); 325 (330); 269 (5300)

X-ray Crystal Structure Determination

The X-ray diffraction data for complexes **1**–**7** were collected using Oxford Diffraction four-circle diffractometer Gemini A Ultra with Atlas CCD detector using graphite monochromated MoKα radiation (λ = 0.71073 Å) at room temperature. Diffraction data collection, cell refinement and data reduction were performed using the CrysAlis^{Pro} software[1]. The structures were solved by the direct methods using SHELXS and refined by full-matrix least-squares on F^2 using SHELXL-2014[2]. All the non-hydrogen atoms were refined anisotropically, and hydrogen atoms were placed in calculated positions and refined with riding constrains: d(C–H) = 0.93 Å, U_{iso}(H) = 1.2 U_{eq}(C) (for aromatic) and d(C–H) = 0.96 Å, U_{iso}(H) = 1.5 U_{eq}(C) (for methyl). The methyl groups were allowed to rotate about their local threefold axis. For structures **1**, **4** and **7** which show severe disorder of solvent molecules, OLEX2 solvent mask command was employed[3]. Details of the crystallographic data collection, structural determination, and refinement for **1**–**7** are given in Table S1, whereas selected bond lengths and angles for these systems are listed in Tables S2–S8.

Physical Measurements

IR spectra were recorded on a Nicolet iS5 FT–IR spectrophotometer in the spectral range 4000–400 cm^{-1} with the samples in the form of KBr pellets (Figure S8). Elemental analyses (C, H, N) were performed on a Perkin–Elemer CHN–2400 analyzer. The electronic spectra were obtained using Nicolet Evolution 220 in the range 240–1000 nm in DMSO and MeOH (Figures S11-S13 and Table S12).

Magnetic measurement

The magnetic measurements were made on a Quantum Design MPMS SQUID magnetometer in the room temperature and magnetic field of 0.5 T. The susceptibilities of **1**−**7** have been corrected for the diamagnetic contribution by using the Pascal’s constants.

EPR spectroscopy

The X–band electron paramagnetic resonance (EPR) spectra of the dipicolinate oxovanadium(IV) complexes were measured using a Bruker ELEXYS E 500 Spectrometer equipped with NMR teslametr and X–band frequency (9.7 GHz, microwave frequency 6.231 GHz, power of 10 mW and modulation amplitude of 8 G). The solid compound **1**, **2** dissolved in water and a few drops of DMSO were added to the samples to ensure good glass formation at liquid nitrogen temperature. Anisotropic

spectra were recorded on frozen solutions at 77 K using quartz Dewar and glass capillary tubes at room temperature. The experimental spectra were simulated with the DoubletExact (S=1/2) computer program[4].

Lipophilicity

The *n*-octanol/PBS buffer partition coefficients (logP) were determined by shake-flash approach, and the transfer of V(IV) compounds from the aqueous environment to the organic phase was monitored by UV-Vis spectroscopy[5,6]. Solutions of **1–10** of concentrations in the range 10-100 μM were prepared in PBS buffer saturated with n-octanol from the freshly-prepared stock solution in DMSO (5mM or 50 mM), and their UV-Vis spectra were recorded. The solution of **1–10** in aqueous buffer (3 ml) were mixed with *n*-octanol saturated with pH 7.4 aqueous buffer (3 ml), and the obtained mixtures were stirred in a mechanical shaker for 30 min. The mixtures were allowed to stand at room temperature for 24 h, and after this time, the aqueous phases were separated, and the concentrations of **1–10** were determined by measuring their UV-Vis spectra. For each complex, the highest energy absorption band was taken into consideration to determine the sample concentration.

Cell culture and maintenance

The A2780 cancer cell line (ovarian carcinoma) was purchased from Sigma-Aldrich (Madrid, Spain) while HCT116 cancer cell line (colorectal carcinoma) and the normal human primary dermal fibroblasts were obtained from American Type Culture Collection (ATCC®, Manassas, VA, USA). HCT116 Dox-resistant cell line (HCT116-DoxR) was derived from HCT116 parental cells, as described in Pedrosa, P., *et al., (2018)*[7].

All cells were cultured in Dulbecco's modified Eagle medium (DMEM), except the A2780 cell line which was cultured in RPMI (Roswell Park Memorial Institute) medium. Both media were supplemented with 10% (v/v) fetal bovine serum (FBS) and 1% (v/v) of a Pen/Strep (Penicillin/Streptomycin) solution. HCT116-DoxR cell medium was additionally supplemented with 3.6 μM Doxorubicin (Dox) to maintain drug resistance. All media and supplements were purchased from Thermo Fischer Scientific (Waltham, Massachusetts, USA).

Cells were maintained in 25/75 cm^2 T-flasks in a humidified atmosphere at 37ºC and 5% (v/v) CO_2 (SANYO CO_2 Incubator, Electric Biomedical Co., Osaka, Japan).

Stability of vanadium complexes in biological medium

The stability of vanadium complexes in biological medium was evaluated by UV-Vis spectroscopy (between 220-700 nm). Complexes were initially solubilized in 100 % (v/v) DMSO and afterwards each complex was diluted with colourless (without *phenol red*) RPMI medium to a final concentration of 50 μM. A quartz cuvette with 1 cm path length was used and spectra were obtained after 0 h, 24 h and 48 h on the spectrophotometer Evolution 300 UV-Vis (Thermo Fischer Scientific, Waltham, MA, USA).

Cell viability assays

HCT116, HCT116-DoxR and A2780 cancer cell lines and normal human primary fibroblasts were used in cellular viability assays. Cells were seeded in a density of 0.75×10^5 cells/mL into 96 well-plates and incubated at 37ºC and 5% (v/v) CO_2 for 24 h. After the 24 h of incubation, complexes were weight and immediately solubilized in 100% (v/v) DMSO and then, diluted for the final concentration with cell culture medium. The medium in each 96 well-plates will be replaced by fresh medium containing each vanadium complex (concentrations ranging from 0.01 μM to 50 μM). Culture medium with 0.1% (v/v) DMSO and 0.4 μM Dox were used as negative and positive controls, respectively. Following a second incubation period of 48 h in the same conditions, cells' viability was assessed with the CellTiter 96® Aqueous One Solution Cell Proliferation Assay kit (Promega, Madison, USA)[8]. When cells are metabolically active, its mitochondrial dehydrogenases can reduce 3-(4,5-dimethylthiazol-2-yl)-5-(3-carboxymethoxyphenyl)-2-(4-sulfophenyl)-2H-tetrazolium, inner salt (MTS) to formazan, whose absorbance can be measured at 490 nm in a microplate reader, Tecan Infinite M200 (Tecan, Männedorf, Switzerland). Thus, formazan's absorbance is directly proportional to the number of viable cells[9,10].

By analysing the cell viability vs. concentration graphics, obtained using Prism 8 (GraphPad software), it is possible to determine the IC_{50} (complex concentration which induces a 50% reduction in cell viability) of each complex in the respective cell line. The selective index (SI) for every complex was also determined, dividing the IC_{50} of human fibroblasts by the IC_{50} of HCT116, HCT116-DoxR or

A2780 cancer cell lines, respectively.

3D-spheroid formation and cell viability assays

HCT116-DoxR cells were seeded at 5000 cells/well in super low Attachment 96-well plates (Nunclon™ Sphera™ U-Shaped-Bottom Microplate, Thermo Fisher Scientific, Waltham, MA, USA) and incubated at 37°C, in a humidified atmosphere with 5% (v/v) CO_2, to allow spheroids formation and growth.

For MTS assay, HCT116-DoxR spheroids with 6 to 8 days were used. At the 6th day of growth, culture media was replaced by medium with vanadium complexes at desired concentrations. Spheroids were then incubated for 48 h in a humidified atmosphere at 37ºC and 5% (v/v) CO_2 and, after the incubation time, medium was replaced by a mixture containing the MTS reagent and DMEM medium (20:100). Spheroids were incubated for another 4 h period and later transferred into a 96-well plate with flat bottom in order to be analysed in the microplate reader Tecan Infinite M200 (Tecan, Männedorf, Switzerland).

ICP-AES (Inductively coupled plasma - atomic emission spectrometry)

Internalization of vanadium complexes in the HCT116-DoxR cell line was assessed by ICP-AES. To do so, HCT116-DoxR cell line was seeded in 25 cm^2 T-flasks in a density of 5×10^5 cells/T-flask and incubated for 24 h in a humidified atmosphere at 37ºC and 5% (v/v) CO_2. After the 24 h incubation, the culture media was replaced by fresh medium containing different vanadium complexes with 10 times its IC_{50} concentrations or 0.1% (v/v) DMSO, and cells were later incubated for another 3 h period in the same conditions. Then, culture media was recovered, and cells were washed with PBS 1x, also recovered. The recovered culture media and PBS 1x were centrifuged at 800 xg (Sigma 3–16K Sartorius, Germany) for 5 min to separate the supernatant and some cells that might be present on them. Cells were detached from each T-flask with 2 mL of TrypLE Express and were centrifuged at 750 xg for 5 min. Fresh aqua regia (3:1 HCl/HNO_3) was added to supernatants and cellular pellets, and every sample were incubated at RT for 24 h in a hood fume. Samples were later delivered to Laboratório de Análises/LAQV and the levels of vanadium were evaluated by ICP-AES.

Evaluation of apoptosis induction in HCT116-DoxR cell line by flow cytometry

The induction of apoptosis in HCT116-DoxR cell line was evaluated by flow cytometry, using the Alexa Fluor® 488 Annexin V/Dead Cell Apoptosis Kit (Invitrogen, Thermo Fisher Scientific, MA, USA). HCT116-DoxR cell line was seeded at a cell density of 1×10^5 cells/mL in 6-well plates and incubated for a 24 h period. Later, culture media was replaced by medium with different vanadium complexes at its IC_{50} concentrations. Culture medium with 0.1% (v/v) DMSO, 6 µM Dox or 5 µM Cisplatin were used as controls. After 48 h of incubation, cells were washed with PBS 1x, detached from each well with TrypLE Express (Invitrogen) and washed again with PBS 1x. Cells were then resuspended in 5x annexin-binding buffer and incubated at RT (room temperature) for 15 min with Alexa® Fluor 488 annexin V and 100 µg/mL PI.

All samples were analysed by an Attune® Acoustic Focusing Flow Cytometer (Life Technologies, Carlsbad, USA) and results were processed with Attune® Cytometric software.

Western Blot for BAX, Bcl-2 and PARP-1 Quantification

HCT116-DoxR cell line was cultivated in 25 cm^2 T-flasks at a cell density of 2×10^6 cells/T-flask and incubated for 24 h in the conditions previously described. After 24 h, the culture media was replaced by fresh medium with different vanadium complexes at its IC_{50} concentrations or 0.1% (v/v) DMSO. It was additionally prepared a 25 cm^2 T-flask containing untreated cells as a control. Cells were incubated for another 48 h period and were later washed and collected using cold PBS 1x and a cell scraper. The samples were centrifuged for 5 min at 700 xg (Sigma 3–16K Sartorius, Germany) and resuspended in fresh lysis buffer (150 mM NaCl; 5 mM ethylenediaminetetraacetic acid (EDTA); 50 mM Tris-HCl pH 8.0; 2% (v/v) NP-40 (Thermo Fisher Scientific, Waltham, MA, USA); 1x phosphatase inhibitors (PhosStop, Roche); 1x protease inhibitors (complete ULTRA tablets, Mini, EASYpack, Roche, Switzerland); 1 mM phenylmethylsulfonyl fuoride (PMSF, Sigma, St. Louis, USA) and 0.1% 1,4-dithiothreitol (v/v) (DTT, Amresco, USA).

Cells were then submitted to 5 ultrasound cycles on ice (2 min on ultrasounds followed by a 30 s period on ice; Elma D-78224; Singen/Htw, Germany) and later centrifuged at 1000 xg for 5 min. Total protein extract (from supernatant) was quantified with Pierce 660 nm Protein Assay Reagent (Thermo Fisher Scientific, Waltham, MA, USA).

For the SDS-PAGE of BAX and Bcl-2, 20 μg of protein was loaded on 10% polyacrylamide gel and transferred to a 0.45 μm PVDF membrane (GE Healthcare Life Sciences, Germany). Each membrane was incubated 2 h with 5% (w/v) non-fat milk or Bovine Serum Albumin (BSA) solubilized in TBST 1x buffer (50 mM Tris-HCl, pH 7.5, 150 mM NaCl, 0.1% (v/v) Tween-20) and later incubated for 1 h, at RT and constant agitation, with a solution containing the respective primary antibody in 5% non-fat milk in TBST 1x, specifically anti-BAX (1:5000; Abcam, United Kingdom) and anti Bcl-2 (1:1000; Sigma, St. Louis, USA). PARP 1 was first transferred into a 0.45 μm nitrocellulose membrane (GE Healthcare Life Sciences, Germany) and later, the membrane was incubated overnight at 4 ºC under constant agitation, with a solution containing the respective primary antibody in 5% BSA in TBST 1x (1:750; Thermo Fisher Scientific, Waltham, MA, USA).

After that, membranes were washed three times with TBST 1x buffer for 5 min each, under constant agitation. This procedure was repeated in the incubation with the secondary antibody (1:3000, Anti-mouse IgG, horseradish peroxidase (HPR)-linked Antibody or 1:2000, Anti-rabbit IgG, HPR-linked Antibody; Cell Signalling Technology, USA). In order to identify the protein bands, membranes were treated with WesternBright ECL substrate (Advansta, USA) for 5 min and film was exposed to the membrane on a dark room. Membranes were later incubated for two times with Stripping buffer (0.1 M glycine, 20 mM magnesium acetate, 50 mM KCl, pH 2.0) during 10 and 20 min, respectively, under agitation and then, incubated with anti-β actin (1:5000; Sigma, St. Louis, USA) as a control to normalize the results. Proteins' quantification was made by densitometry with Image J software.

Evaluation of mitochondrial membrane potential ($\Delta\Psi_m$) in HCT116-DoxR cell line by flow cytometry

$\Delta\Psi_m$ was evaluated in HCT116-DoxR cell line by using the JC-1 Mitochondrial Membrane Potential Assay Kit (Abnova Corporation, Walnut, CA, USA). Cells were seeded at a density of 1×10^5 cells/mL in 6-well plates and, after 24 h of incubation, culture media was replaced by medium with different vanadium complexes at its IC_{50} concentrations. 0.1% (v/v) DMSO was used as a negative (vehicle) control while 6 μM Dox and 5 μM Cisplatin were used as positive controls. Cells were then incubated for 48 h and after that, cells were washed with PBS 1x, detached with TrypLE Express and washed again with DMEM medium. Cells were later resuspended in DMEM medium without *phenol red* + 5% (v/v) FBS and incubated with JC-1 in DMEM medium without *phenol red* + 5% (v/v) FBS for 20 min at 37ºC. The cells were then resuspended in DMEM medium without *phenol red* + 5% (v/v) FBS and the analysis of mitochondrial membrane potential was performed in the Attune® Acoustic Focusing Flow Cytometer (Life Technologies, Carlsbad, USA).

Caspase-8 activity

The Caspase-8 activity in HCT116-DoxR cells exposed to vanadium complexes was evaluated using the Caspase-8 Assay kit (ab39700) (Abcam, Cambridge, UK). HCT116-DoxR cell line was cultivated in 25 cm^2 T-flasks at a cell density of 2×10^6 cells/T-flask and incubated for 24 h in a CO_2 incubator at 37ºC, 5% (v/v) CO_2 and 99% (v/v) relative humidity. After 24 h, medium was replaced with fresh medium supplemented with 0.1% (v/v) DMSO, 5 μM Cisplatin or the IC_{50} concentration of the desired vanadium complexes.

After 48 h of incubation, cells were detached with a cell scraper, collected into eppendorfs, and centrifuged at 4ºC and 500 xg for 5 min. Cells were then washed with 1 mL of cold PBS and centrifuged at 750 xg and 4ºC for 5 min. Right after, the pellet was resuspended in 50 μL of cold Lysis Buffer and the cells were incubated on ice for 20 min, being then centrifuged at 1000 xg and 4ºC for 10 min. Duplicate wells were prepared in a 96-well plate with 200 μg of protein in Lysis Buffer for a final volume of 50 μL of sample. It was added to each well 50 μL of the freshly prepared Reaction mix as well as 5 μL of IETD-pNA. The plate was then incubated under the same conditions as described above for 2 h and then absorbance was read at 400 nm on a Tecan Infinite M200 microplate reader (Männedorf, Switzerland).

Evaluation of autophagy induction in HCT116-DoxR cell line by flow cytometry

Autophagy induction in HCT116-DoxR cell line was evaluated using the Autophagy Assay Kit (ab139484) (Abcam, Cambridge, United Kingdom) according to the manufacturer's instructions. Briefly, HCT116-DoxR cell line was seeded in a density of 1×10^5 cells/mL in 6-well plates and, after an initial incubation time of 24 h, culture media was replaced by medium with different vanadium complexes at its IC_{50} concentrations. DMSO 0.1% (v/v), 6 μM Dox and 5 μM Cisplatin were also used

as controls. Cells were incubated for another 48 h, and 1,5 μM Rapamycin was added 18 h before finishing the 48 h period. After that, cells were firstly washed with PBS 1x and then detached with TrypLE Express, followed by another wash with DMEM medium without *phenol red* + 5% (v/v) FBS. Cells were then incubated with Green Stain solution in DMEM medium without *phenol red* + 5% (v/v) FBS for 30 min at RT. After the incubation period, cells were washed and later resuspended with Assay Buffer 1x. The evaluation of autophagy induction occurred due to the Attune® Acoustic Focusing Flow Cytometer (Life Technologies, Carlsbad, USA) and the results were analysed via the Attune® Cytometric software.

Evaluation of reactive oxygen species (ROS) production in HCT116-DoxR cell line by flow cytometry

HCT116-DoxR cell line was seeded in 6-well plates in a density of 1×10^5 cells/mL and incubated for 24 h. After that time, the culture media was replaced by medium with different vanadium complexes at its IC_{50} concentrations and DMSO 0.1% (v/v), 6 μM Dox, 5 μM Cisplatin and 42 μM TBHP (Tert-Butyl hydroperoxide) were used as controls. After that, cells were incubated for another 48 h period. Later, cells were washed with PBS 1x, detached from each well with TrypLE Express, washed again with PBS 1x and later incubated with 10 μM of 2′,7′- dichlorodihydrofluorescein diacetate (H_2DCF-DA) (Thermo Fisher Scientific, Waltham, MA, USA) in PBS 1x, for 20 min. Cells were later analysed with the Attune® Acoustic Focusing Flow Cytometer (Life Technologies, Carlsbad, USA) and the obtained data was processed by the respective software (Attune® Cytometric software).

Cell cycle progression

HCT116-DoxR cells were seeded in 6-well plates in a density of 1×10^5 cells/mL, incubated for 8 h in a humidified atmosphere at 37ºC and 5% (v/v) CO_2, and later synchronized in early S-phase with a 2 mM thymidine solution (Sigma, St. Louis, USA) for a 16 h period. Media with thymidine was replaced by fresh media and, after 8 h, another 2 mM thymidine solution was added to the wells (double thymidine block). Cells were incubated for 16 h and then culture media was replaced by medium with the vanadium complexes at its IC_{50} concentrations. 0.1% (v/v) DMSO was used as the vehicle control while 5 μM cisplatin and 6 μM doxorubicin as the positive controls. To normalize the results, untreated cells were collected right after the double thymidine block (0 h). Treated cells were then incubated for 3 h, 9 h, 24 h or 32 h and, after each time point, cells were treated with TrypLE Express and centrifuged at 650 xg for 5 min, at 4ºC. The resultant pellet was resuspended in 1 mL of cold PBS 1x and centrifuged at 3000 xg for 5 min and 4ºC. After this, the pellet was resuspended in 100 μL of PBS 1x, and 1 mL of a solution of 80% (v/v) ethanol was gently added to the cells. Cells were stored at 4°C for at least 12 h and, after incubation, cells were centrifuged for 10 min at 7500 xg at 4°C, treated with 250 μL of 50 μg/mL RNase A and incubated for 30 min at 37°C. After incubation, 100 μL of PI (25 μg/mL) was added to the cells as well as 650 μL of PBS 1x. The DNA content was evaluated on Attune® Acoustic Focusing Flow Cytometer (Life Technologies, Carlsbad, USA) and results were analysed by the respective software (Attune® Cytometric software).

BSA binding assays

All solutions used in the BSA interaction studies were diluted in filtered buffer (10 mM phosphate and 150 mM NaCl) at pH 7.0, with BSA at a fixed final concentration of 20 μM and the concentrations of the complexes varying from 10 μM to 100 μM. Control solutions were also prepared, namely a solution containing only BSA, a solution consisting of BSA + DMSO and a solution consisting of DMSO only. Solutions were analysed after 24 hours of incubation at 37ºC. The UV-Vis spectra were obtained from 245 nm to 500 nm on the spectrophotometer Evolution 300 UV-Vis (Thermo Fischer Scientific, Waltham, MA, USA) while the fluorescence spectra were recorded through an emission range of 290 to 500 nm after an excitation wavelength of 278 nm (excitation and emission slit widths of 5 nm), on a Varian Cary Eclipse fluorescence spectrophotometer (Agilent Technologies, CA, USA).

Cell Migration assay

Fibroblasts were seeded in 24-well plates in a density of 4×10^5 cells/mL and grown in the same conditions as described before until a confluent monolayer was obtained. Cells were later exposed to 0.1% (v/v) DMSO or the respective IC_{50} concentrations of vanadium complexes and, with a sterile 200 μL micropipette tip, a scratch was made on the surface of each well. Fibroblasts were photographed right after exposure (0 h) and 24 h after incubation at 37ºC and 5% (v/v) CO_2 in a humidified atmosphere, using a Ti-U Eclipse inverted microscope (Nikon, Japan). The scratches were analysed

via ImageJ software.

Ex-ovo **Chick Chorioallantoic Membrane (CAM) assay**

The *ex-ovo* chorioallantoic membrane (CAM) assay was performed in order to evaluate the angiogenic potential of vanadium complexes, as already described in literature[11–13]. Firstly, chicken embryos were incubated for a 24 h period, to stabilize the embryos and later, a solution containing the IC_{50} concentration of each complex dissolved in PBS 1x was placed in the centre of O-rings. Beside each complex, DMSO 0.1% (v/v) was used as a control and complex distribution in chicken embryos was always performed in a different order. The embryos were incubated for 48 h at 37ºC and images were captured after 0 h, 24 h and 48 h, using a digital USB Microscope Camera (Opti-Tekscope OT-V1). Newly formed blood vessels were later manually counted, via ImageJ software.

The *ex-ovo* CAM assay fulfils the Directive 2010/63/ EU of the European Parliament for protection of animal models for scientific purposes.

Statistical analysis

All data are presented as Mean ± SEM from at least two independent experiments unless otherwise specified. One-way ANOVA or Student's t-test were used to determine statistical significance ($p < 0.05$) using the GraphPad Prism 8 software (GraphPad Software, San Diego, CA, USA).

HPLC

HPLC measurements were carried out on the apparatus Kanuer liquid chromatograph with UV-VIS detection and Agilent 1100 liquid chromatograph, as well as Waters 600 liquid chromatograph equipped with UV-Vis detector. In reference to limited reports on this subject [14-17], the methanol–water, acetonitrile–water or methanol–acetonitrile–water phases were used. As demonstrated by selected chromatograms below, however, this method was found to be unsuitable for systems of our type, most probably due to their poor affinity for the methanol–water, acetonitrile–water or methanol–acetonitrile–water phases.

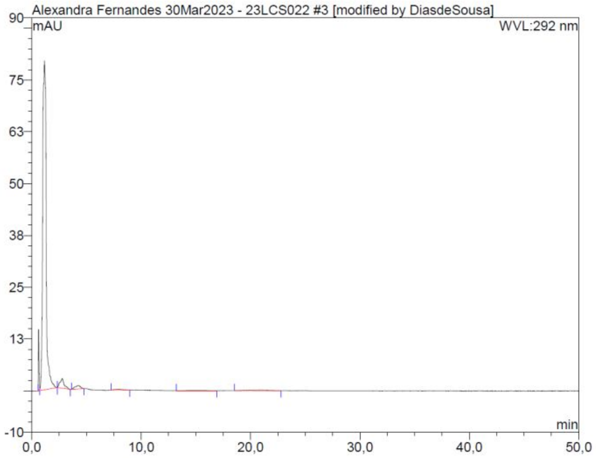

Compound **1**

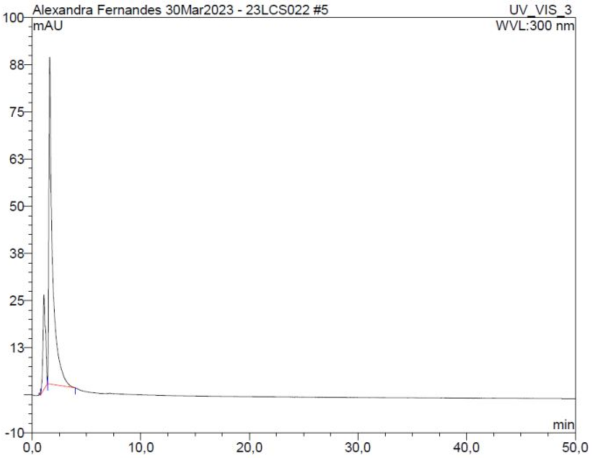

Compound **2**

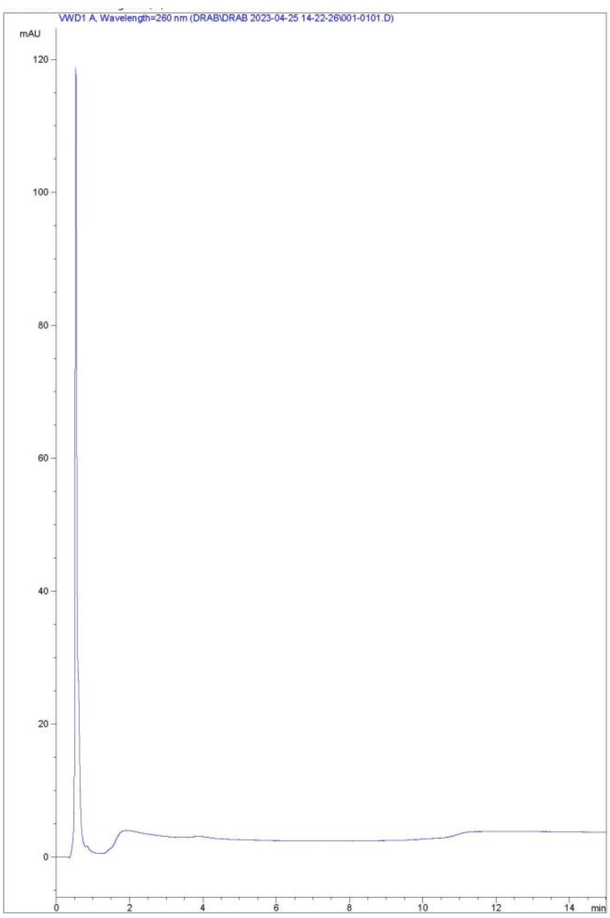

Compound **5**

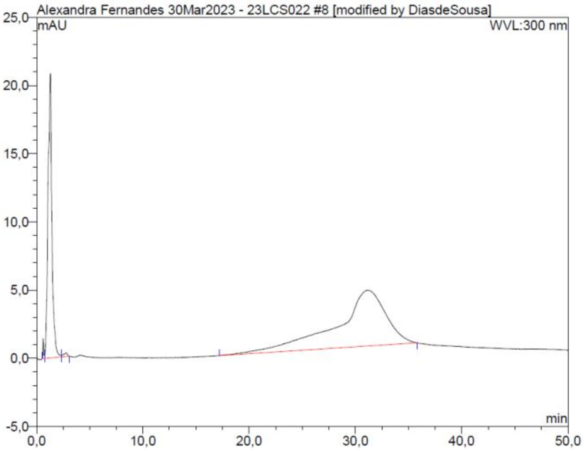

Compound **6**

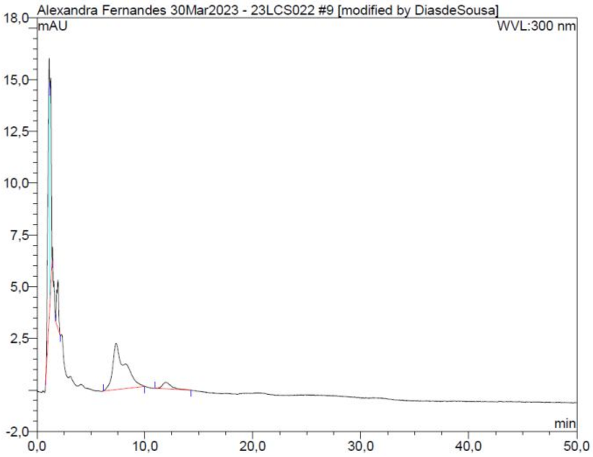

Compound **7**

X-Ray studies

Table S1. Crystal data and structure refinement for **1–7**

	**1**	**2**	**3**	**4**	**5**	**6**	**7**
Empirical formula	$C_{15}H_{10}N_4O_5V$	$C_{20}H_{16}N_4O_6V$	$C_{23}H_{15}N_5O_7V$	$C_{19}H_9Cl_2N_3O_5V$	$C_{23}H_{17}Cl_6N_3O_7V$	$C_{31}H_{19}N_3O_5V$	$C_{43}H_{27}N_5O_6S_2V$
Formula weight	377.21	459.31	524.34	481.13	711.03	564.43	824.75
Temperature [K]	295.0(2)	295.0(2)	295.0(2)	295.0(2)	295.0(2)	295.0(2)	295.0(2)
Wavelength [Å]	0.71073	0.71073	0.71073	0.71073	0.71073	0.71073	0.71073
Crystal system	monoclinic	monoclinic	monoclinic	monoclinic	triclinic	monoclinic	orthorhombic
Space group	$P2_1/n$	$P2_1/n$	$P2_1$	$P2_1/n$	$P\bar{1}$	$P2_1/c$	*Pnma*
Unit cell dimensions [Å, °]	a = 22.6912(10)<br>b = 6.1324(3)<br>c = 24.9591(9)<br>β = 100.058(4)	a =11.9450(5)<br>b = 6.6833(5)<br>c = 24.7879(13)<br>β = 101.595(5)	a = 8.3153(3)<br>b = 11.3332(4)<br>c = 24.8437(9)<br>β = 96.942(3)	a = 15.6702(6)<br>b = 8.1111(3)<br>c = 16.1598(7)<br>β = 102.096(4)	a = 10.7590(7)<br>b = 10.8569(7)<br>c = 13.4386(10)<br>α = 74.153(6)<br>β = 75.342(6)<br>γ = 78.613(6)	a = 11.7552(6)<br>b = 21.8550(13)<br>c = 9.9806(5)<br>β = 95.596(5)	a = 16.9518(9)<br>b = 11.1613(9)<br>c = 23.8345(11)
Volume [Å ^{3}]	3419.7(3)	1938.50(19)	2324.08(15)	2008.35(14)	1446.99(18)	2551.9(2)	4509.6(5)
Z	8	4	4	4	2	4	4
Density (calculated) [Mg/m ^{3}]	1.465	1.574	1.499	1.591	1.632	1.469	1.215
Absorption coefficient [mm $^{-1}$]	0.613	0.560	0.482	0.797	0.944	0.437	0.361
F(000)	1528	940	1068	964	714	1156	1692
Crystal size [mm]	0.21 × 0.12 × 0.09	0.15 × 0.10 × 0.05	0.26 × 0.13 × 0.05	0.12 × 0.11 × 0.06	0.15 × 0.14 × 0.06	0.31 × 0.26 × 0.12	0.23 × 0.19 × 0.17
θ range for data collection [°]	3.50 to 25.05	3.67 to 25.05	3.32 to 25.05	3.67 to 25.05	3.94 to 25.05	3.48 to 25.05	3.37 to 25.05
Index ranges	$-23 \le h \le 27$<br>$-7 \le k \le 7$<br>$-28 \le l \le 29$	$-14 \le h \le 14$<br>$-6 \le k \le 7$<br>$-29 \le l \le 22$	$-9 \le h \le 8$<br>$-13 \le k \le 13$<br>$-29 \le l \le 29$	$-18 \le h \le 18$<br>$-9 \le k \le 9$<br>$-19 \le l \le 18$	$-12 \le h \le 12$<br>$-12 \le k \le 12$<br>$-15 \le l \le 15$	$-14 \le h \le 13$<br>$-26 \le k \le 25$<br>$-11 \le l \le 11$	$-20 \le h \le 20$<br>$-11 \le k \le 13$<br>$-28 \le l \le 20$
Reflections collected	15471	9168	18029	11062	13448	14278	12873
Independent reflections	6042 (R_{int} = 0.0616)	3424 (R_{int} = 0.0372)	8147 (R_{int} = 0.0322)	3546 (R_{int} = 0.0305)	5100 (R_{int} = 0.0618)	4504 (R_{int} = 0.0314)	4203 (R_{int} = 0.0365)
Max. and min. transmission	1.000 and 0.699	1.000 and 0.681	1.000 and 0.804	1.000 and 0.870	1.000 and 0.440	1.000 and 0.442	1.000 and 0.589
Data / restraints / parameters	6042 / 0 / 451	3424 / 0 / 282	8147 / 1 / 673	3546 / 0 / 271	5100 / 0 / 363	4504 / 0 / 361	4203 / 0 / 292
Goodness-of-fit on F^2	1.060	1.055	1.087	1.037	1.096	1.066	1.066
Final R indices [$I > 2\sigma(I)$]	R_1 = 0.0778<br>wR_2 = 0.1921	R_1 = 0.0421<br>wR_2 = 0.0907	R_1 = 0.0485<br>wR_2 = 0.1130	R_1 = 0.0342<br>wR_2 = 0.0837	R_1 = 0.0787<br>wR_2 = 0.2226	R_1 = 0.0372<br>wR_2 = 0.1031	R_1 = 0.0574<br>wR_2 = 0.1534
R indices (all data)	R_1 = 0.1074<br>wR_2 = 0.2047	R_1 = 0.0638<br>wR_2 = 0.0968	R_1 = 0.0558<br>wR_2 = 0.1168	R_1 = 0.0439<br>wR_2 = 0.0880	R_1 = 0.1156<br>wR_2 = 0.2503	R_1 = 0.0524<br>wR_2 = 0.0955	R_1 = 0.846<br>wR_2 = 0.1649
Largest diff. peak and hole[e Å $^{-3}$]	0.746 and -0.450	0.258 and -0.334	0.340 and -0.262	0.255 and -0.340	0.951 and -0.701	0.197 and -0.331	0.248 and -0.297
CCDC	2233583	2233578	2233582	2233579	2233584	2233580	2233581

Table S2. Selected bond lengths (Å) and angles (deg) for complex **1**

Bond lengths		Bond angles	
V(1)–N(1)	2.029(5)	N(1)–V(1)–N(2)	88.54(18)
V(1)–N(2)	2.381(5)	N(1)–V(1)–N(3)	159.7(2)
V(1)–N(3)	2.084(5)	N(2)–V(1)–N(3)	71.91(19)
V(1)–O(1)	1.579(5)	N(1)–V(1)–O(1)	106.6(2)
V(1)–O(2)	2.072(4)	N(1)–V(1)–O(2)	76.59(18)
V(1)–O(4)	2.021(4)	N(1)–V(1)–O(4)	76.98(17)
		N(2)–V(1)–O(1)	163.8(2)
		N(2)–V(1)–O(2)	80.41(18)
		N(2)–V(1)–O(4)	86.85(17)
		N(3)–V(1)–O(1)	93.4(2)
		N(3)–V(1)–O(2)	104.43(18)
		N(3)–V(1)–O(4)	96.21(18)
		O(1)–V(1)–O(2)	97.3(2)
		O(1)–V(1)–O(4)	101.8(2)
		O(2)–V(1)–O(4)	150.86(19)
V(2)–N(5)	2.027(5)	N(5)–V(2)–N(6)	89.73(19)
V(2)–N(6)	2.380(5)	N(5)–V(2)–N(7)	160.8(2)
V(2)–N(7)	2.080(5)	N(6)–V(2)–N(7)	72.06(18)
V(2)–O(6)	1.590(5)	N(5)–V(2)–O(6)	106.4(2)
V(2)–O(7)	2.026(4)	N(6)–V(2)–O(6)	162.9(2)
V(2)–O(9)	2.045(4)	N(7)–V(2)–O(6)	92.4(2)
		N(5)–V(2)–O(7)	76.49(18)
		N(6)–V(2)–O(7)	86.71(17)
		N(7)–V(2)–O(7)	96.00(18)
		N(5)–V(2)–O(9)	76.74(18)
		N(6)–V(2)–O(9)	79.15(17)
		N(7)–V(2)–O(9)	104.78(17)
		O(6)–V(2)–O(7)	102.3(2)
		O(6)–V(2)–O(9)	98.7(2)
		O(7)–V(2)–O(9)	149.67(19)

Table S3. Selected bond lengths (Å) and angles (deg) for complex **2**

Bond lengths		Bond angles	
V(1)–N(1)	2.024(2)	N(1)–V(1)–N(2)	85.05(8)
V(1)–N(2)	2.393(2)	N(1)–V(1)–N(3)	156.60(10)
V(1)–N(3)	2.092(2)	N(2)–V(1)–N(3)	71.95(8)
V(1)–O(1)	1.579(2)	N(1)–V(1)–O(1)	107.02(10)
V(1)–O(2)	2.0655(17)	N(1)–V(1)–O(2)	76.39(8)
V(1)–O(4)	1.9924(19)	N(1)–V(1)–O(4)	77.12(8)
		N(2)–V(1)–O(1)	166.78(9)
		N(2)–V(1)–O(2)	79.93(8)
		N(2)–V(1)–O(4)	84.22(8)
		N(3)–V(1)–O(1)	96.29(9)
		N(3)–V(1)–O(2)	103.01(7)
		N(3)–V(1)–O(4)	95.86(8)
		O(1)–V(1)–O(2)	97.39(10)
		O(1)–V(1)–O(4)	103.42(10)
		O(2)–V(1)–O(4)	150.10(8)

Table S4. Selected bond lengths (Å) and angles (deg) for complex **3**

**Bond lengths**		**Bond angles**	
V(1)–N(1)	2.016(5)	N(1)–V(1)–N(2)	89.10(18)
V(1)–N(2)	2.316(5)	N(1)–V(1)–N(3)	161.63(19)
V(1)–N(3)	2.132(5)	N(2)–V(1)–N(3)	72.91(17)
V(1)–O(1)	1.595(4)	N(1)–V(1)–O(1)	106.4(2)
V(1)–O(2)	2.008(5)	N(1)–V(1)–O(2)	76.81(19)
V(1)–O(4)	2.024(5)	N(1)–V(1)–O(4)	76.9(2)
		N(2)–V(1)–O(1)	164.5(2)
		N(2)–V(1)–O(2)	82.90(18)
		N(2)–V(1)–O(4)	83.84(18)
		N(3)–V(1)–O(1)	91.6(2)
		N(3)–V(1)–O(2)	96.81(18)
		N(3)–V(1)–O(4)	104.13(19)
		O(1)–V(1)–O(2)	100.9(2)
		O(1)–V(1)–O(4)	98.9(2)
		O(2)–V(1)–O(4)	150.64(18)
V(2)–N(4)	2.037(5)	N(4)–V(2)–N(5)	89.72(19)
V(2)–N(5)	2.313(5)	N(4)–V(2)–N(6)	162.1(2)
V(2)–N(6)	2.125(5)	N(5)–V(2)–N(6)	72.41(18)
V(2)–O(8)	1.593(4)	N(4)–V(2)–O(8)	107.0(2)
V(2)–O(9)	2.021(5)	N(4)–V(2)–O(9)	76.87(18)
V(2)–O(11)	2.021(4)	N(4)–V(2)–O(11)	76.84(18)
		N(5)–V(2)–O(8)	163.3(2)
		N(5)–V(2)–O(9)	83.69(18)
		N(5)–V(2)–O(11)	84.10(18)
		N(6)–V(2)–O(8)	90.9(2)
		N(6)–V(2)–O(9)	100.68(18)
		N(6)–V(2)–O(11)	100.54(18)
		O(8)–V(2)–O(9)	99.4(2)
		O(8)–V(2)–O(11)	99.9(2)
		O(9)–V(2)–O(11)	150.97(17)

Table S5. Selected bond lengths (Å) and angles (deg) for complex **4**

**Bond lengths**		**Bond angles**	
V(1)–N(1)	2.0270(19)	N(1)–V(1)–N(2)	89.57(7)
V(1)–N(2)	2.3166(18)	N(1)–V(1)–N(3)	161.43(7)
V(1)–N(3)	2.1277(19)	N(2)–V(1)–N(3)	72.94(7)
V(1)–O(1)	1.5907(16)	N(1)–V(1)–O(1)	105.33(8)
V(1)–O(2)	1.9997(17)	N(1)–V(1)–O(2)	77.22(7)
V(1)–O(4)	2.0354(16)	N(1)–V(1)–O(4)	76.53(7)
		N(2)–V(1)–O(1)	165.02(8)
		N(2)–V(1)–O(2)	83.52(7)
		N(2)–V(1)–O(4)	82.99(7)
		N(3)–V(1)–O(1)	92.39(8)
		N(3)–V(1)–O(2)	94.24(7)
		N(3)–V(1)–O(4)	106.65(7)
		O(1)–V(1)–O(2)	100.98(8)
		O(1)–V(1)–O(4)	98.80(8)
		O(2)–V(1)–O(4)	150.46(7)

Table S6. Selected bond lengths (Å) and angles (deg) for complex **5**

**Bond lengths**		**Bond angles**	
V(1)–N(1)	2.029(3)	N(1)–V(1)–N(2)	86.23(14)
V(1)–N(2)	2.307(4)	N(1)–V(1)–N(3)	157.76(16)
V(1)–N(3)	2.123(4)	N(2)–V(1)–N(3)	73.36(14)
V(1)–O(1)	1.587(4)	N(1)–V(1)–O(1)	107.30(17)
V(1)–O(2)	2.017(3)	N(1)–V(1)–O(2)	76.78(14)
V(1)–O(4)	2.043(3)	N(1)–V(1)–O(4)	76.89(13)
		N(2)–V(1)–O(1)	166.16(16)
		N(2)–V(1)–O(2)	85.42(15)
		N(2)–V(1)–O(4)	82.04(14)
		N(3)–V(1)–O(1)	93.67(16)
		N(3)–V(1)–O(2)	92.56(14)
		N(3)–V(1)–O(4)	108.08(14)
		O(1)–V(1)–O(2)	100.29(18)
		O(1)–V(1)–O(4)	97.87(17)
		O(2)–V(1)–O(4)	151.46(13)

Table S7. Selected bond lengths (Å) and angles (deg) for complex **6**

**Bond lengths**		**Bond angles**	
V(1)–N(1)	2.0259(18)	N(1)–V(1)–N(2)	92.58(6)
V(1)–N(2)	2.2979(16)	N(1)–V(1)–N(3)	164.69(6)
V(1)–N(3)	2.1343(18)	N(2)–V(1)–N(3)	72.38(6)
V(1)–O(1)	1.5924(15)	N(1)–V(1)–O(1)	104.23(7)
V(1)–O(2)	2.0156(15)	N(1)–V(1)–O(2)	76.92(7)
V(1)–O(4)	2.0287(15)	N(1)–V(1)–O(4)	76.43(6)
		N(2)–V(1)–O(1)	162.83(7)
		N(2)–V(1)–O(2)	86.57(6)
		N(2)–V(1)–O(4)	80.41(6)
		N(3)–V(1)–O(1)	90.97(7)
		N(3)–V(1)–O(2)	98.78(6)
		N(3)–V(1)–O(4)	103.11(6)
		O(1)–V(1)–O(2)	100.46(7)
		O(1)–V(1)–O(4)	99.91(7)
		O(2)–V(1)–O(4)	149.66(6)

Table S8. Selected bond lengths (Å) and angles (deg) for complex **7**

**Bond lengths**		**Bond angles**	
V(1)–N(1)	2.024(3)	N(1)–V(1)–N(2)	158.87(13)
V(1)–N(2)	2.126(3)	N(1)–V(1)–N(3)	85.92(12)
V(1)–N(3)	2.305(3)	N(2)–V(1)–N(3)	72.94(11)
V(1)–O(1)	1.592(3)	N(1)–V(1)–O(1)	108.65(15)
V(1)–O(2)	2.006(2)	N(1)–V(1)–O(2)	77.31(6)
V(1)–O(2)[a]	2.006(2)	N(2)–V(1)–O(1)	92.49(13)
		N(2)–V(1)–O(2)	99.44(6)
		N(3)–V(1)–O(1)	165.43(13)
		N(3)–V(1)–O(2)	82.60(7)
		O(1)–V(1)–O(2)	100.24(7)
		O(1)–V(1)–O(2)[a]	100.24(7)
		O(2)–V(1)–O(2)[a]	151.37(13)

[a] O(2) atom is generated by symmetry operation: x,1/2-y,z

Table S9. Short intra- and intermolecular hydrogen bonds in structures **1–7**

D–H•••A	D–H [Å]	H•••A [Å]	D–A [Å]	D–H•••A [°]
**1**				
C(4)–H(4)•••O(8)[a]	0.93	2.46	3.171(8)	134.00
C(17)–H(17)•••O(5)[b]	0.93	2.54	3.289(8)	138.00
**2**				
N(4)–H(4)•••O(6)[c]	0.86	1.93	2.778(3)	169.00
O(6)–H(6)•••O(3)	0.82	1.93	2.737(3)	167.00
C(2)–H(2)•••O(3)[d]	0.93	2.54	3.449(3)	166.00
C(4)–H(4A)•••O(1)[e]	0.93	2.58	3.288(3)	133.00
C(9)–H(9)•••O(1)	0.93	2.56	3.191(3)	125.00
C(16)–H(16)•••O(6)[c]	0.93	2.58	3.458(4)	157.00
C(18)–H(18)•••O(2)[b]	0.93	2.40	3.149(4)	137.00
**3**				
C(3)–H(3)•••O(14)	0.93	2.54	3.210(10) Å	130.00
C(17)–H(17)•••O(1)	0.93	2.43	2.909(9)	112.00
C(17)–H(17)•••O(3)[f]	0.93	2.56	3.358(9)	145.00
C(27)–H(27)•••N(10A)[g]	0.93	2.57	3.34(3)	141.00
C(34)–H(34)•••N(10)[h]	0.93	2.57	3.18(3)	124.00
C(36)–H(36)•••O(8)	0.93	2.43	2.896(9)	111.00
C(40)–H(40A)•••O(12)	0.96	2.32	3.274(12)	172.00
C(42)–H(42A)•••O(8)	0.96	2.50	3.174(10)	127.00
C(44)–H(44A)•••O(6)[i]	0.96	2.60	3.479(10)	153.00
C(46)–H(46E)•••O(7)	0.96	2.36	3.315(14)	176.00
C(46)–H(46F)•••N(9)[j]	0.96	2.57	3.48(2)	158.00
**4**				
C(8)–H(8)•••O(1)	0.93	2.47	2.941(3)	111.00
C(9)–H(9)•••O(1)[k]	0.93	2.45	3.350(3)	162.00
C(12)–H(12)•••Cl(1)	0.93	2.73	3.097(3)	105.00
C(13)–H(13)•••Cl(2)	0.93	2.76	3.119(3)	104.00
C(16)–H(16)•••O(5)[l]	0.93	2.45	3.375(3)	174.00
**5**				
C(2)–H(2)•••O(3)[m]	0.93	2.42	3.222(7)	144.00
C(17)–H(17)•••O(1)	0.93	2.51	2.969(6)	111.00
C(17)–H(17)•••Cl(3)[n]	0.93	2.74	3.584(7)	152.00
C(22)–H(22)•••O(2)	0.98	2.33	3.272(9)	162.00
C(23)–H(23)•••O(5)[o]	0.98	2.12	3.086(10)	168.00
**6**				
C(4)–H(4)•••O(1)[p]	0.93	2.55	3.230(3)	130.00
C(8)–H(8)•••O(1)	0.93	2.46	2.917(3)	110.00
C(9)–H(9)•••O(2)[r]	0.93	2.53	3.343(3)	146.00
C(17)–H(17)•••O(5)[p]	0.93	2.31	3.168(3)	153.00
C(28)–H(28)•••O(2)[s]	0.93	2.59	3.400(3)	146.00
**7**				
O(4)–H(4A)•••O(3)[t]	0.85	2.00	2.846(6)	175.00
O(4)–H(4B)•••O(3)	0.85	2.55	2.846(6)	101.00
C(5)–H(5)•••O(1)	0.93	2.49	2.952(5)	111.00
C(9)–H(9)•••O(1)[u]	0.93	2.44	3.347(5)	165.00

Symmetry codes: (a): x,-1+y,z; (b): x,1+y,z; (c): 1/2-x,1/2+y,1/2-z; (d): -x,2-y,-z; (e): 1-x, 2-y,-z; (f): 2-x,-1/2+y,-z; (g): 1+x,y,1+z; (h): -x,1/2+y,-z; (i): 1+x,y,z; (j): -1+x,y,z; (k): 3/2-x,1/2+y,1/2-z; (l): 5/2-x,-1/2+y,1/2-z; (m): 1-x,1-y,2-z; (n): 1-x,2-y,1-z; (o): x,y,-1+z; (p): x,1/2-y,-1/2+z; (r): 1-x,-y,1-z; (s): -x,-y,-z; (t): x,3/2-y,z; (u): -1/2+x,1/2-y,1/2-z

Table S10. Short π•••π interactions in structures **1–7**

Cg(I)•••Cg(J)	Cg(I)•••Cg(J) [Å]	α [°]	β [°]	γ [°]	Cg(I)-Perp [Å]	Cg(J)-Perp [Å]
**1**						
Cg(1)•••Cg(2)[a]	3.583(4)	1.6(4)	20.81	21.89	3.324(3)	-3.349(3)
Cg(3)•••Cg(4)[a]	3.570(4)	5.8(3)	19.22	20.96	-3.334(3)	3.371(3)
**2**						
Cg(5)•••Cg(6)[a]	3.5916(15)	2.56(12)	24.75	27.23	-3.1936(10)	3.2619(11)
**3**						
no π•••π interactions detected						
**4**						
Cg(12)•••Cg(13)[b]<br>Cg(13)•••Cg(13)[b]	3.8577(14)<br>3.5939(14)	1.43(11)<br>0	26.70<br>17.67	28.02<br>17.67	-3.4057(9)<br>-3.4243(10)	-3.4464(10)<br>-3.4243(10)
**5**						
Cg(8)•••Cg(13)[c]	3.812(3)	2.1(3)	26.93	26.95	3.398(2)	3.399(2)
Cg(13)•••Cg(13)[c]	3.468(3)	0	11.43	11.43	3.399(2)	3.399(2)
**6**						
Cg(13)•••Cg(13)[d]	3.9803(12)	0	8.51	8.51	3.9365(8)	3.9365(8)
**7**						
no π•••π interactions detected						

α = dihedral angle between *Cg*(I) and *Cg*(J); *Cg*(I)-Perp = Perpendicular distance of *Cg*(I) on ring J; *Cg*(J)-Perp = perpendicular distance of *Cg*(J) on ring I; β = angle *Cg*(I)→*Cg*(J) vector and normal to ring I; γ = angle *Cg*(I) →*Cg*(J) vector and normal to plane J

Cg1 is the centroid of atoms N(3)/C(13)/N(4)/C(15)/C(14); Cg2 is the centroid of atoms N(2)/C(8)/C(9)/C(10)/C(11)/C(12); Cg3 is the centroid of atoms N(7)/C(28)/N(8)/C(30)/C(29); Cg4 is the centroid of atoms N(6)/C(23)/C(24)/C(25)/C(26)/C(27); Cg5 is the centroid of atoms N(2)/C(15)/C(16)/C(17)/C(18)/C(19); Cg6 is the centroid of atoms C(8)/C(9)/C(10)/C(11)/C(12)/C(13); Cg7 is the centroid of atoms N(3)/C(14)/C(15)/C(16)/C(17)/C(18); Cg8 is the centroid of atoms N(1)/C(1)/C(2)/C(3)/C(4)/C(5); Cg9 is the centroid of atoms N(7)/C(39)/C(40)/C(41)/C(42)/C(50); Cg10 is the centroid of atoms C(42)/C(43)/C(44)/C(45)/C(49)/C(50); Cg11 is the centroid of atoms N(6)/C(36)/C(35)/C(34)/C(33)/C(37); Cg12 is the centroid of atoms N(2)/C(17)/C(16)/C(15)/C(14)/C(18); Cg13 is the centroid of atoms C(11)/C(12)/C(13)/C(14)/C(18)/C(19)

Symmetry codes: (a): x,1+y,z; (b): 2-x,-y,1-z; (c): 1-x,2-y,2-z; (d): -x,-y,1-z

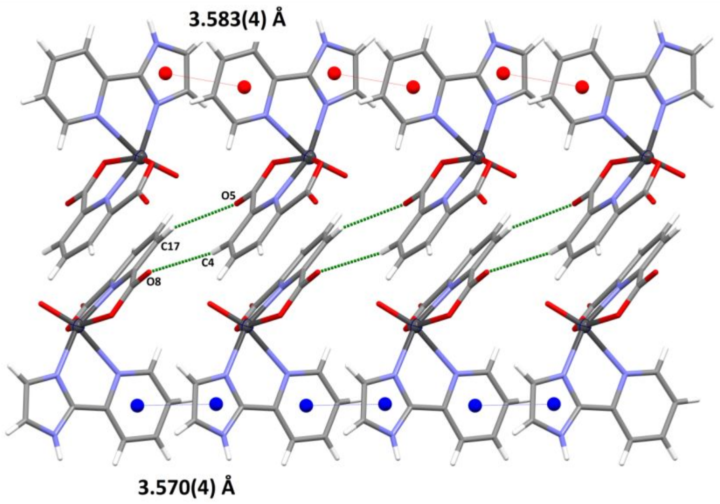

Figure S1. View of the intermolecular interactions (π•••π, and C–H•••O) in **1**

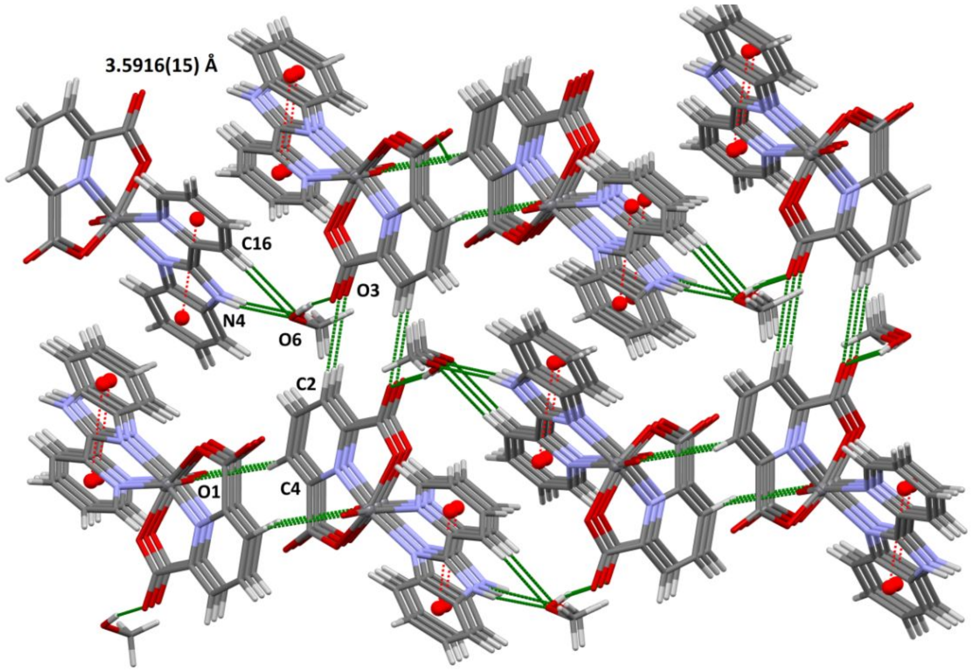

Figure S2. View of the intermolecular interactions (π•••π, N–H•••O, O–H•••O and C–H•••O) in **2**

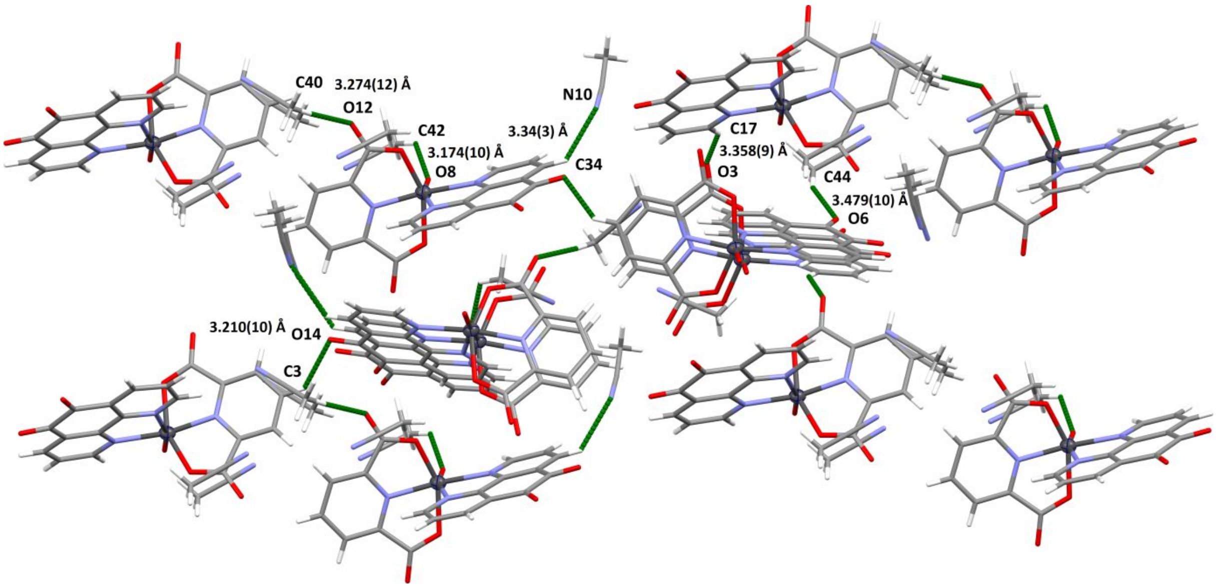

Figure S3. View of the intermolecular interactions (π•••π and C–H•••O) in **3**

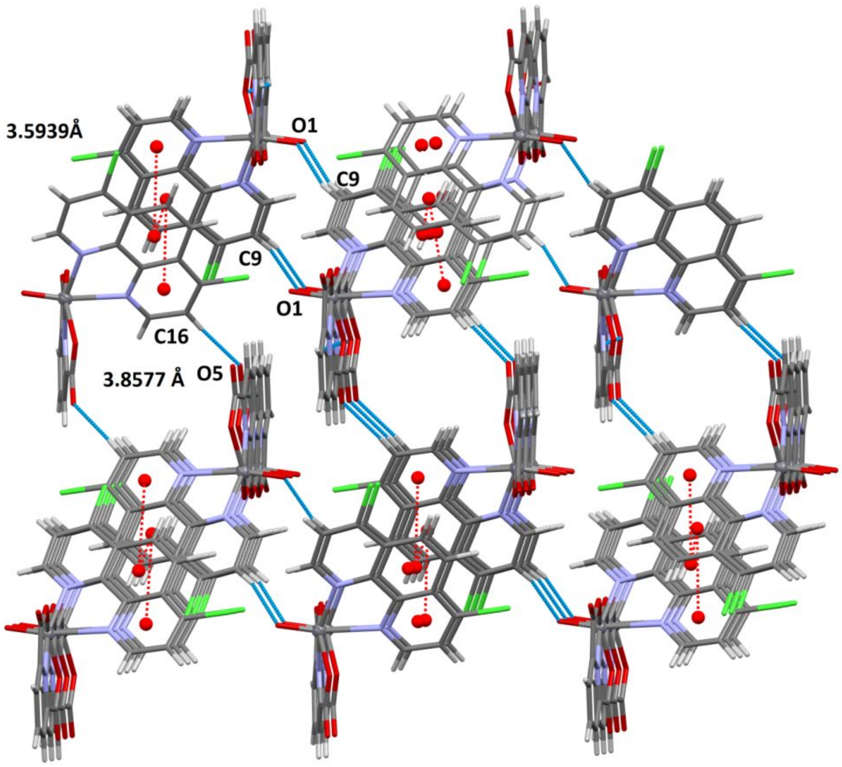

Figure S4. View of the intermolecular interactions (π•••π and C–H•••O) in **4**

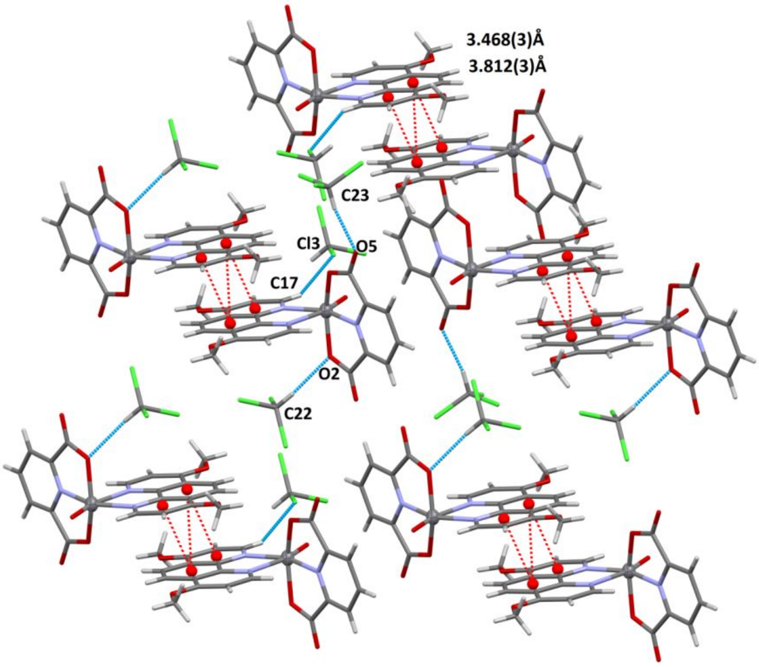

Figure S5. View of the intermolecular interactions (π•••π, C–H•••Cl and C–H•••O) in **5**

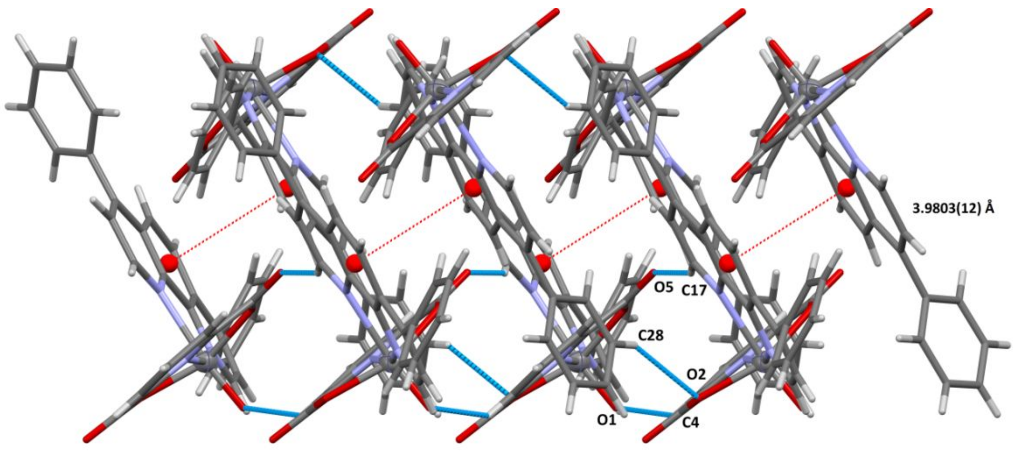

Figure S6. View of the intermolecular interactions (π•••π and C–H•••O) in **6**

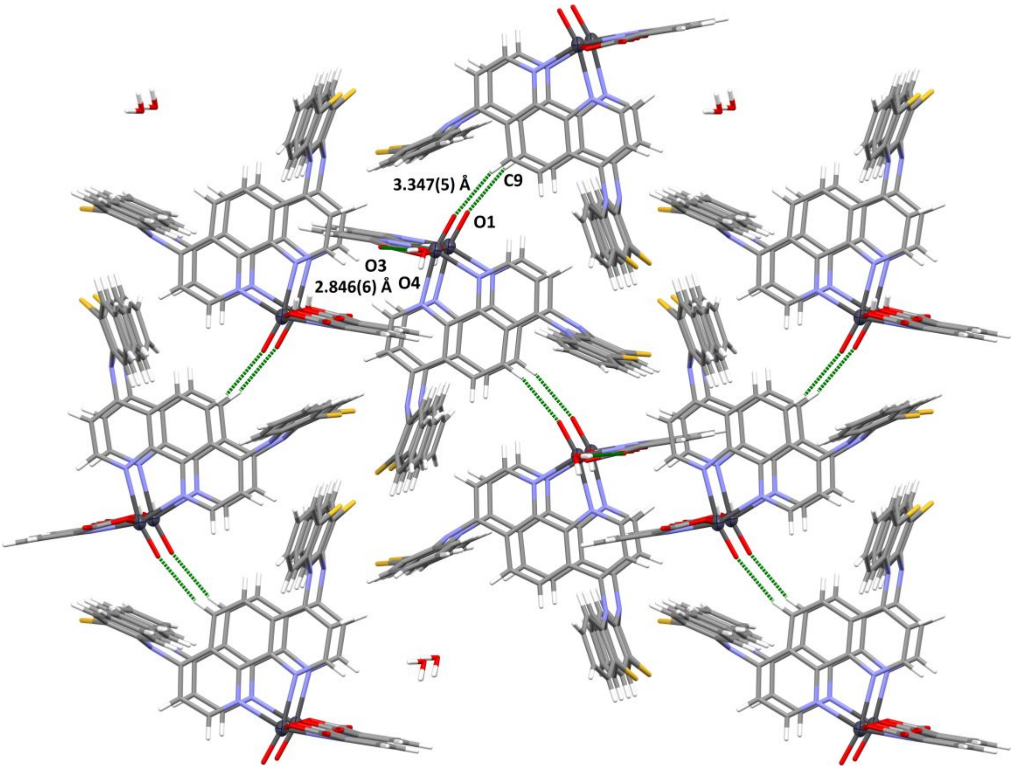

Figure S7. View of the intermolecular interactions (O–H•••O and C–H•••O) in **7**

IR spectroscopy

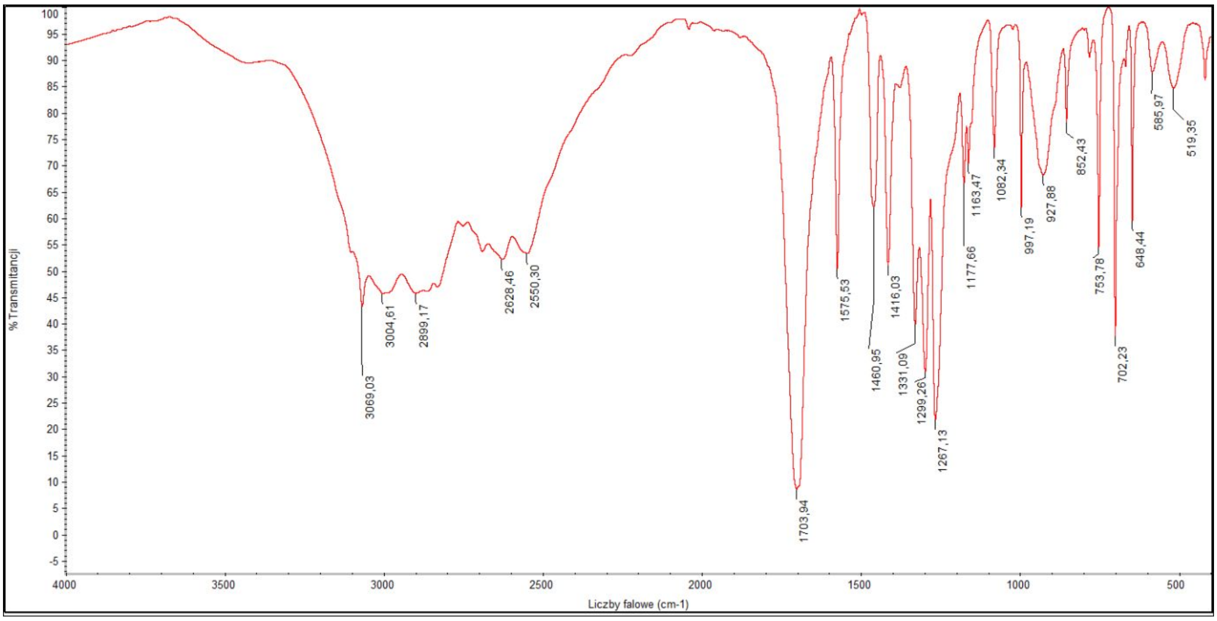

(a) Dipicolinic acid

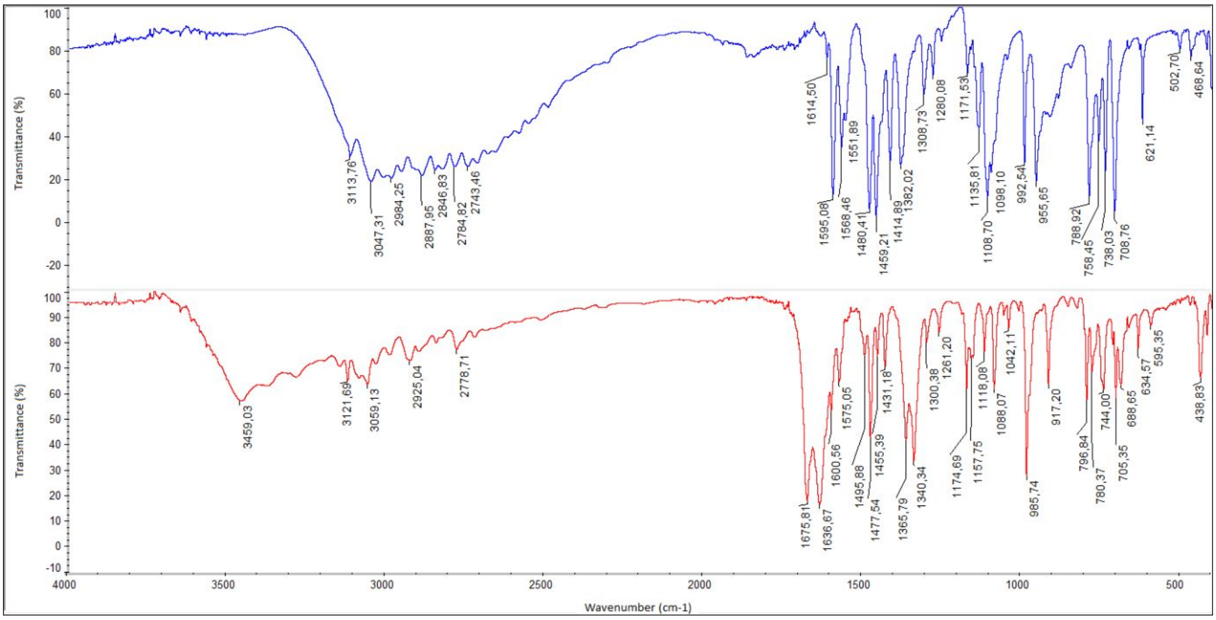

(b) Complex 1 (red) and 2-(1*H*-imidazol-2-yl)pyridine (blue)

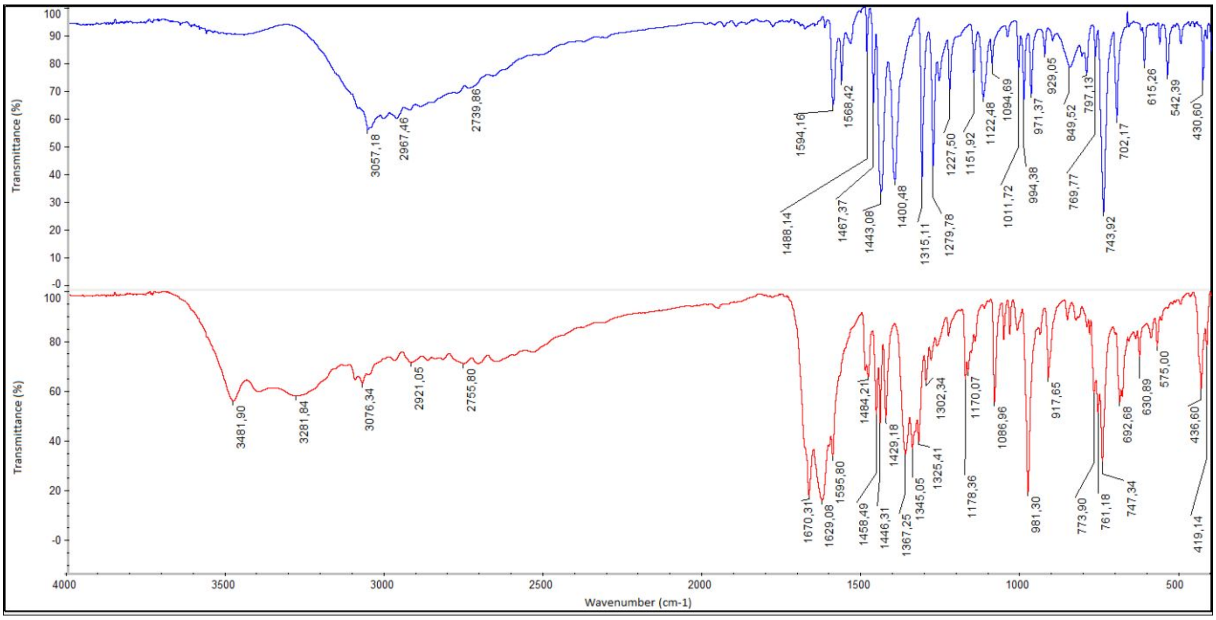

(c) Complex 2 (red) and 2-(2-pyridyl)benzimidazole (blue)

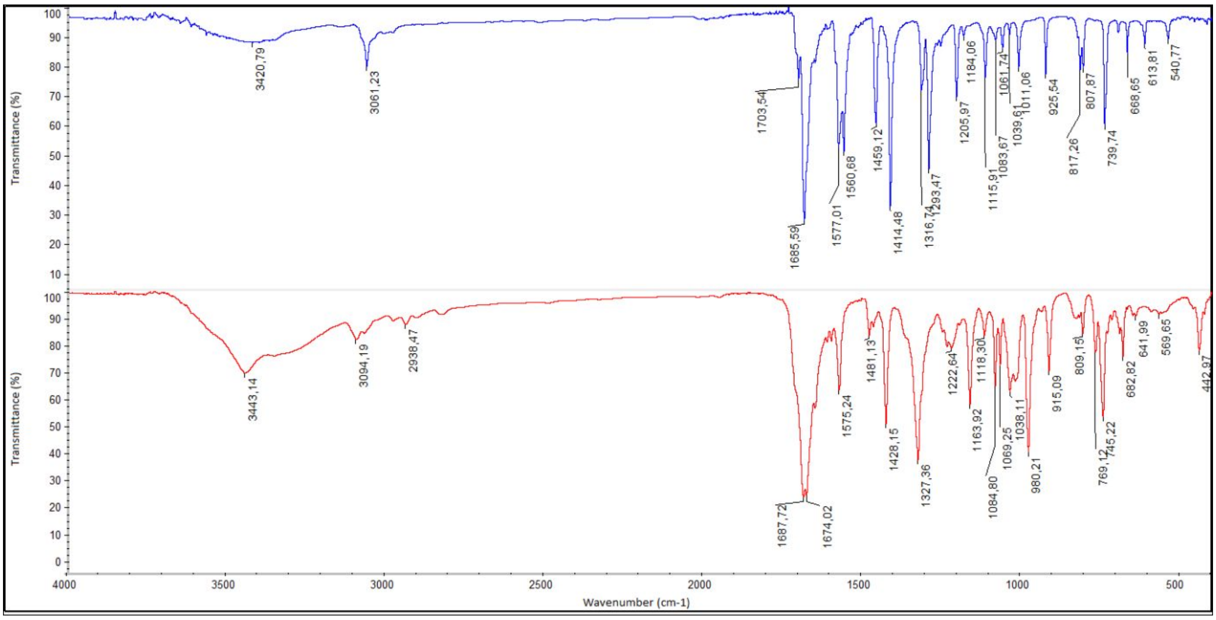

(d) Complex 3 (red) and 1,10-phenanthroline-5,6-dione (blue)

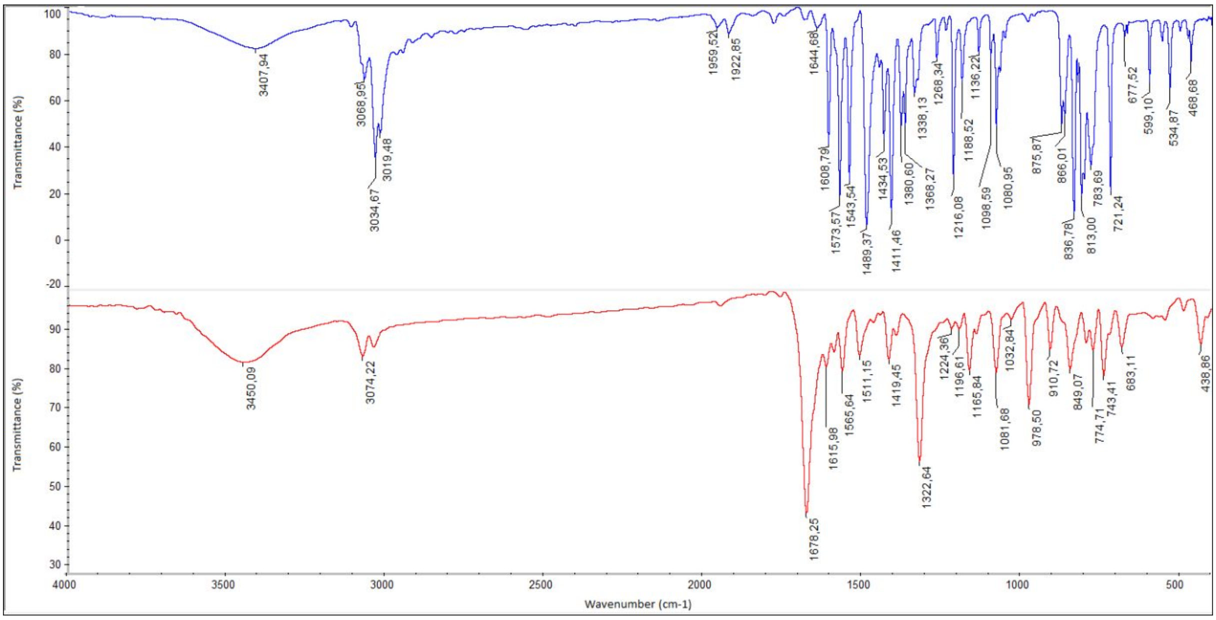

(e) Complex 4 (red) and 4,7-Cl_2-phen (blue)

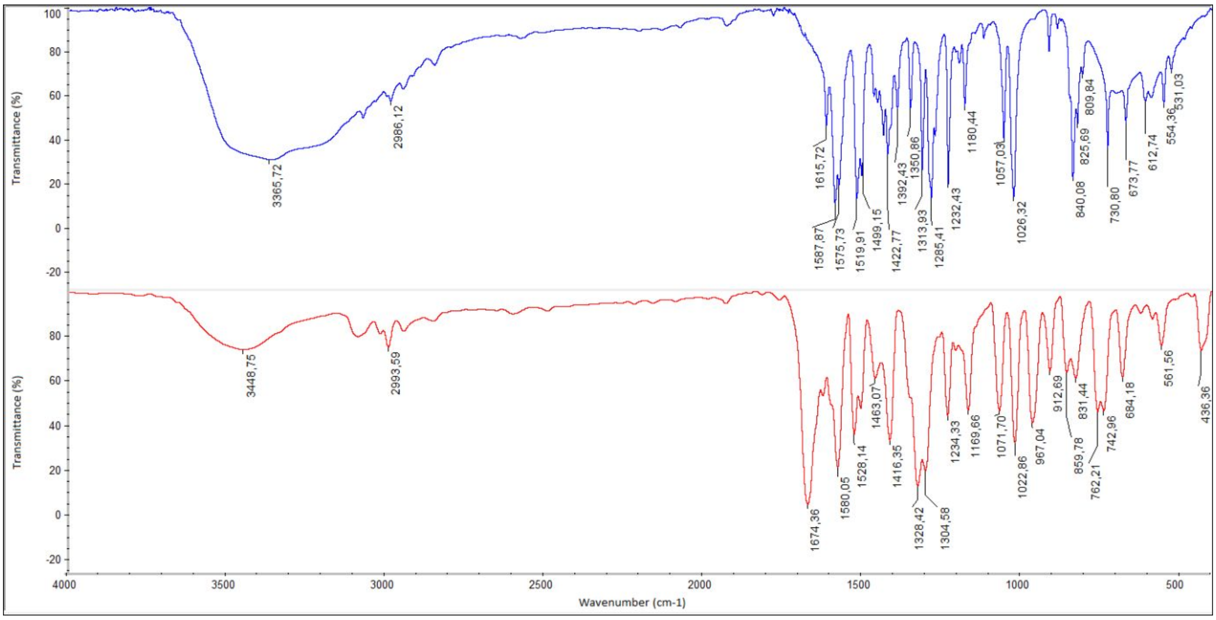

(f) Complex 5 (red) and 4,7-$(CH_3O)_2$-phen (blue)

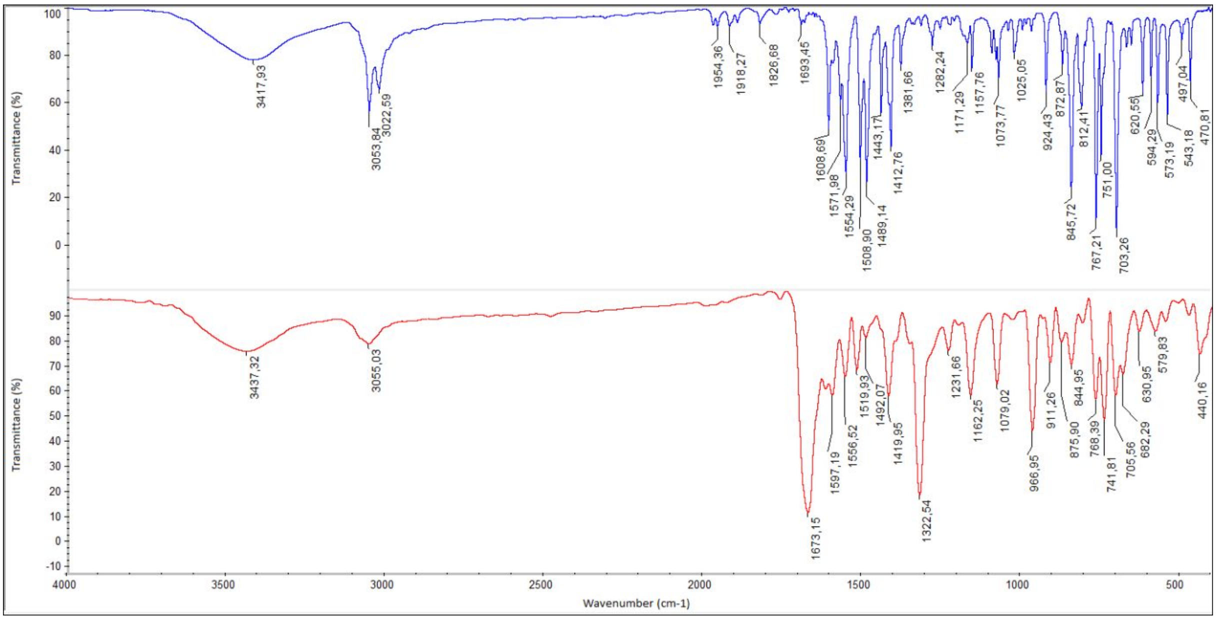

(g) Complex 6 (red) and 4,7-Ph_2-phen (blue)

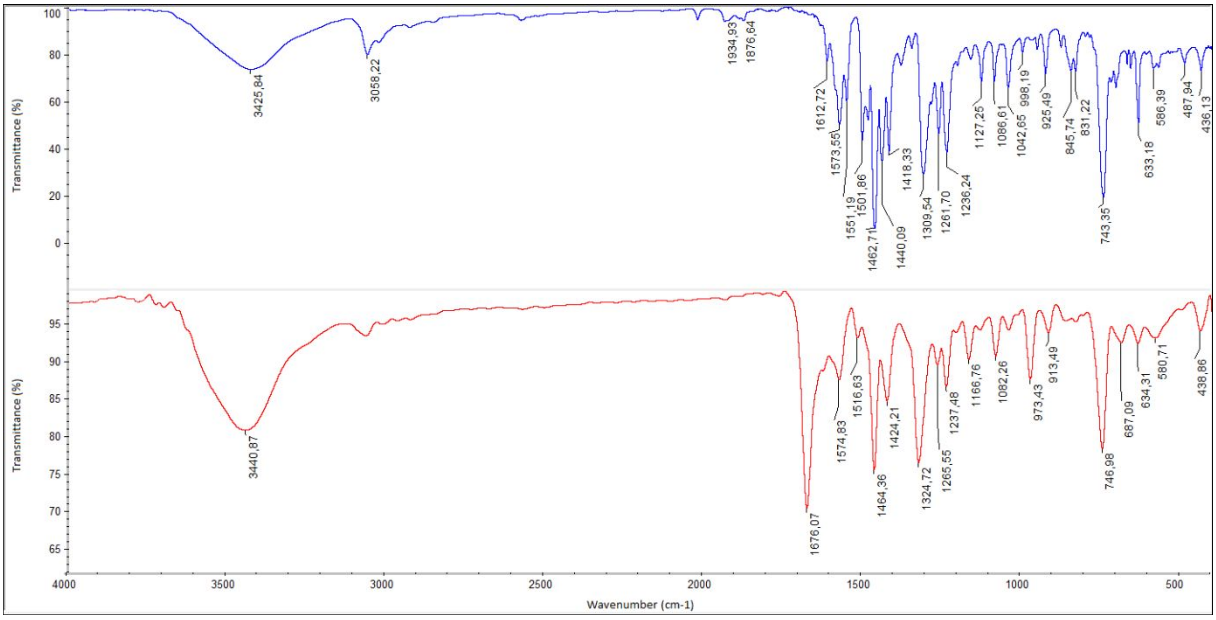

(h) Complex 7 (red) and 4,7-$(phtiaz)_2$-phen (blue)

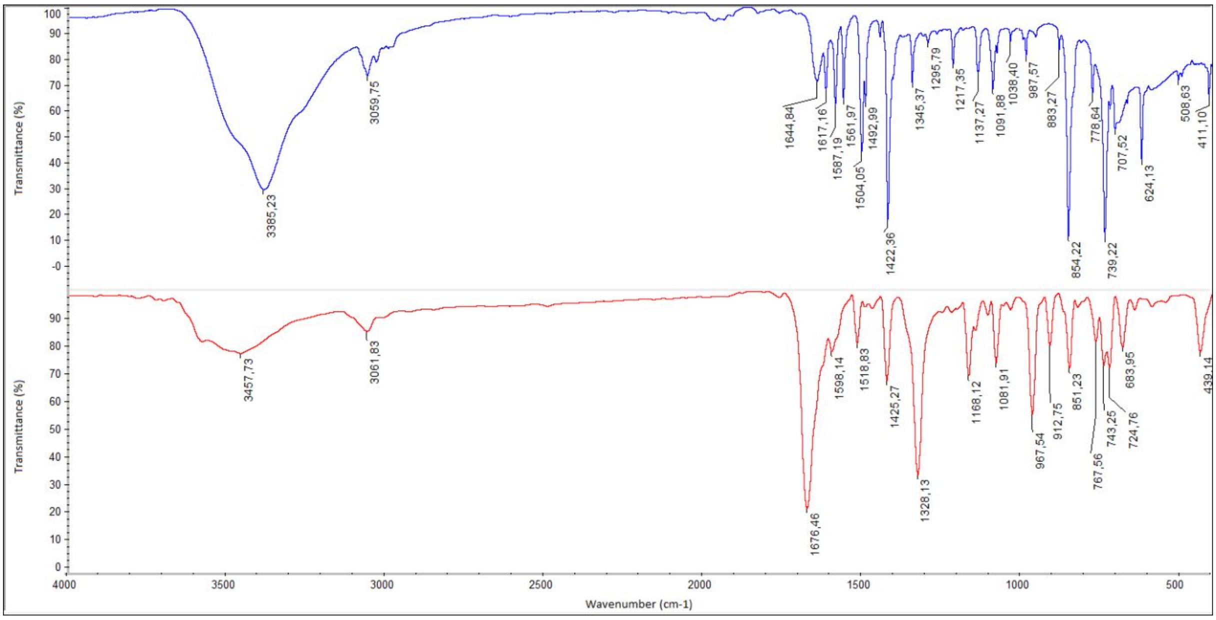

(i) Complex 8 (red) and 1,10-phenanthroline (blue)

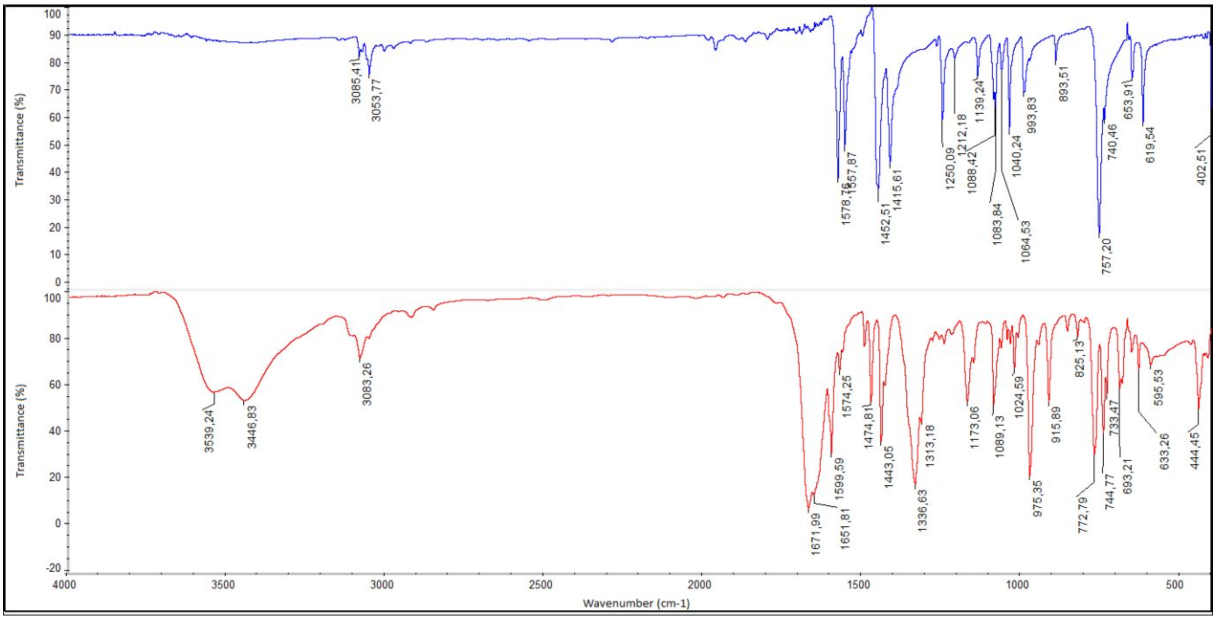

(j) Complex 9 (red) and 2,2′-bipyridine (blue)

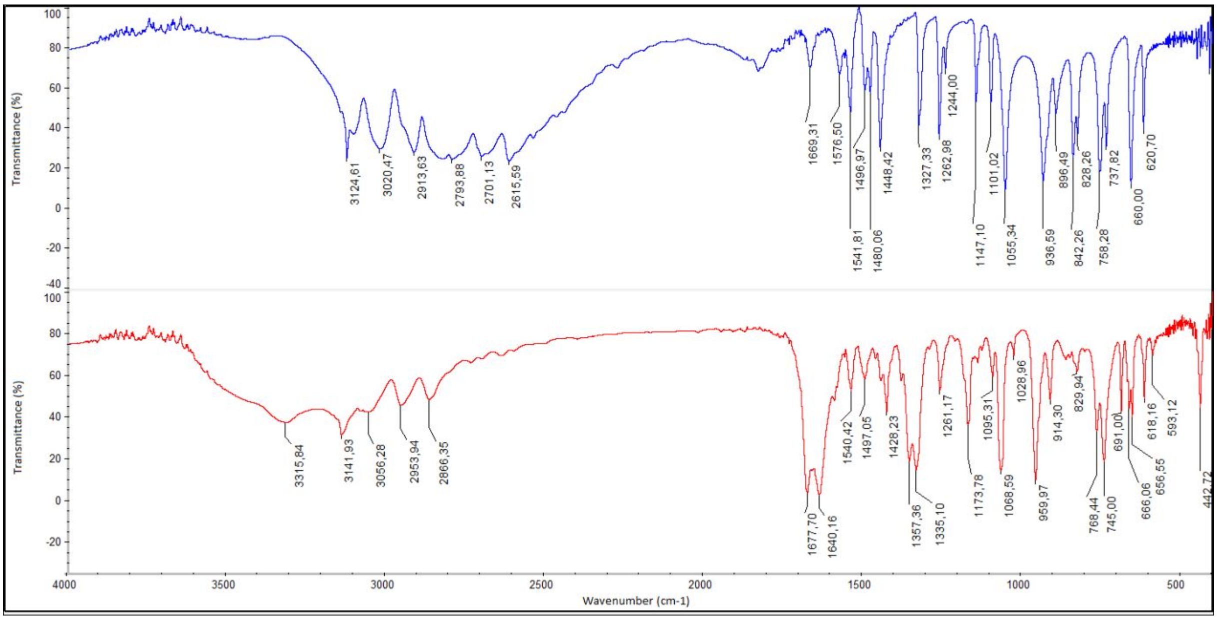

(k) Complex 10 (red) and imidazole (blue)

Figure S8. IR spectra of the dipicolinic acid (a) and complexes **1–10** (red) in comparison to free ligands (blue) (b–k)

EPR spectroscopy

Table S11. Magnetic moments of **1**–**7**.

Compound	μ [μ_B] exp.	*g*	μ_{eff} [μ_B]*
**1**	1.76	1.993	1.73
**2**	1.78	1.979	1.72
**3**	1.77	1.986	1.72
**4**	1.75	1.990	1.73
**5**	1.79	1.983	1.72
**6**	1.80	1.987	1.72
**7**	1.76	1.984	1.72

*The expected high-temperature (300 K) value of magnetic moment $\mu_{eff} = g[S(S + 1)]^{1/2}$ for S = $^1/_2$ spin system and *g* given from EPR spectra .

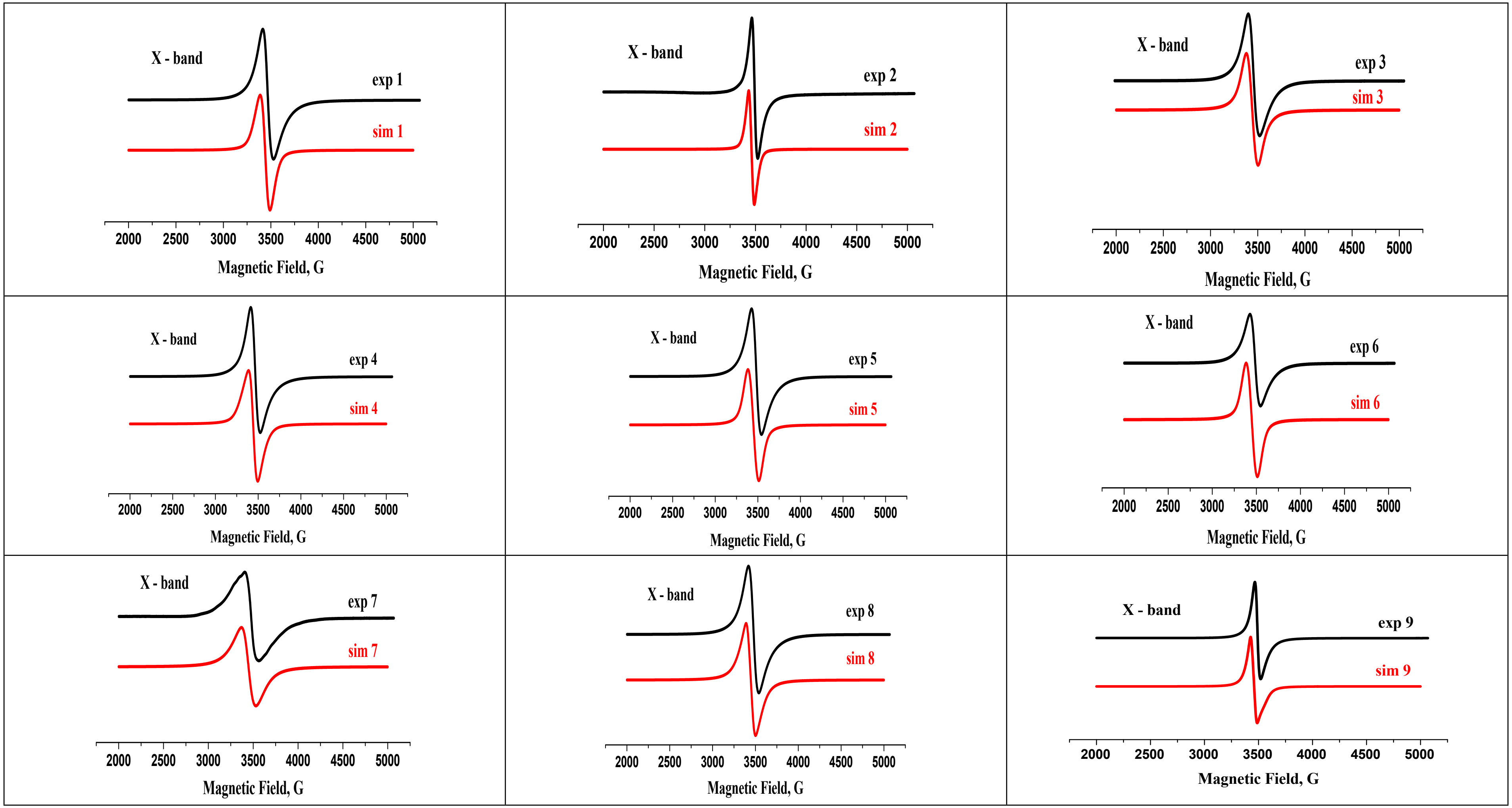
X - band
exp 1
sim 1
2000 2500 3000 3500 4000 4500 5000
Magnetic Field, G
X - band
exp 2
sim 2
2000 2500 3000 3500 4000 4500 5000
Magnetic Field, G
X - band
exp 3
sim 3
2000 2500 3000 3500 4000 4500 5000
Magnetic Field, G
X - band
exp 4
sim 4
2000 2500 3000 3500 4000 4500 5000
Magnetic Field, G
X - band
exp 5
sim 5
2000 2500 3000 3500 4000 4500 5000
Magnetic Field, G
X - band
exp 6
sim 6
2000 2500 3000 3500 4000 4500 5000
Magnetic Field, G
X - band
exp 7
sim 7
2000 2500 3000 3500 4000 4500 5000
Magnetic Field, G
X - band
exp 8
sim 8
2000 2500 3000 3500 4000 4500 5000
Magnetic Field, G
X - band
exp 9
sim 9
2000 2500 3000 3500 4000 4500 5000
Magnetic Field, G

Figure S9. The X-band EPR spectra of dipicolinate oxovanadium(IV) complexes at 77 K together with the spectra calculated by computer simulation of the experimental spectra with average *g* parameters: 1.993 (**1**), 1.979 (**2**), 1.986 (**3**), 1.990 (**4**), 1.983 (**5**), 1.987 (**6**), 1.984 (**7**), 1.979(**8**), 1.981 (**9**).

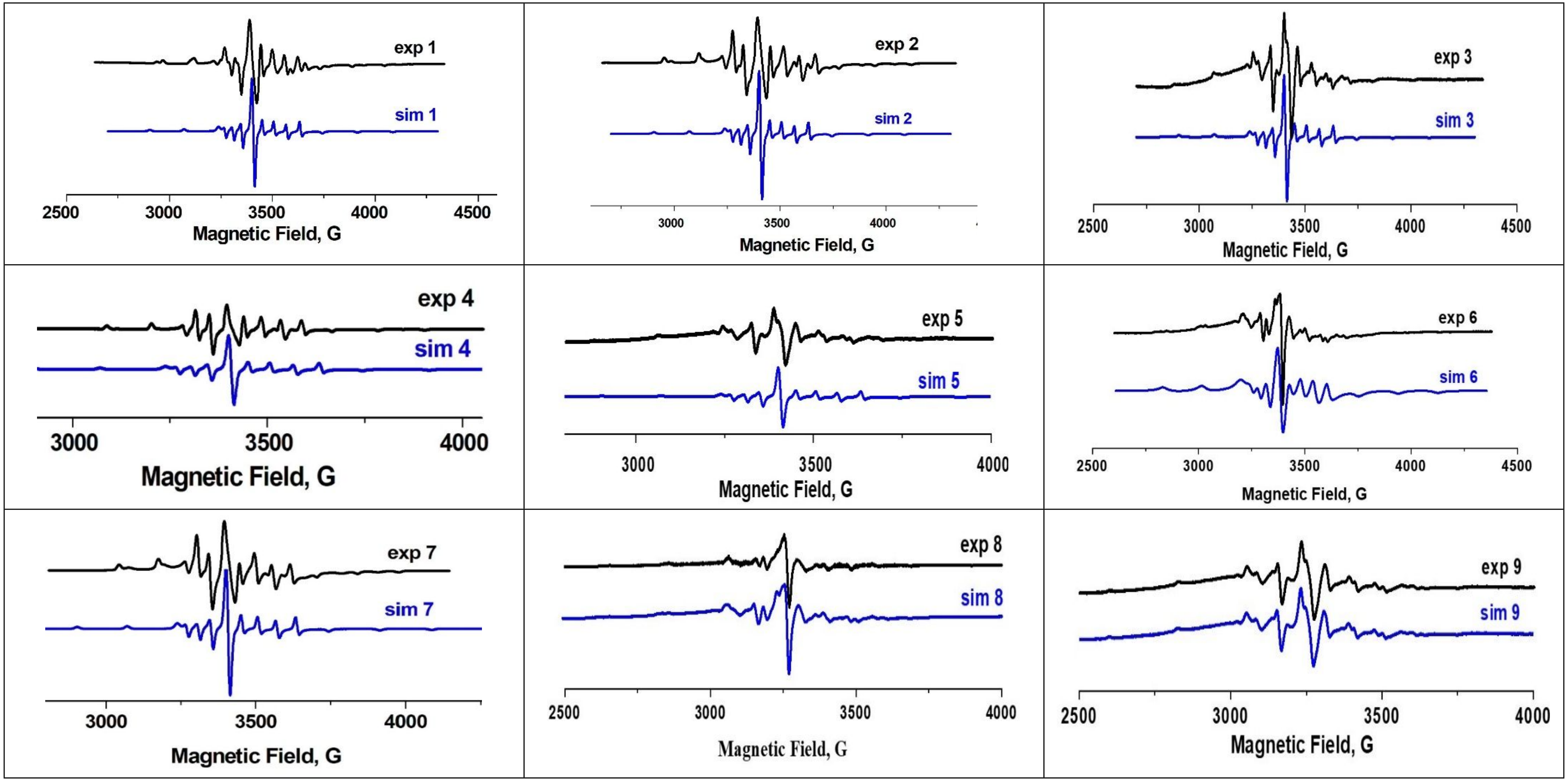

Figure S10. EPR frozen solution spectra (at 77 K) of dipicolinate oxovanadium(IV) complexes in DMSO solvent together with the theoretical spectra calculated using the parameters: $g_x = g_y = 1.997$, $g_z = 1.962$, $A_x = A_y = 65.4$ G, Az = 165 G for **1**, $g_x = g_y = 1.979$, $g_z = 1.958$, $A_x = A_y = 75.3$ G, $A_z = 186$ G for **2,** $g_x = g_y = 1.981$, $g_z = 1.942$, $A_x = A_y = 65.4$ G, $A_z = 180$ G for **3,** $g_x = g_y = 1.984$, $g_z = 1.946$, $A_x = A_y = 68.4$ G, $A_z = 169$ G for **4,** $g_x = g_y = 1.977$, $g_z = 1.958$, $A_x = A_y = 62$ G, $A_z = 1688.5$ G for **5,** $g_x = g_y = 1.984$, $g_z = 1.956$, $A_x = A_y = 71.5$ G, $A_z = 179$ G for **6**, $g_x = g_y = 1.979$, $g_z = 1.950$, $A_x = A_y = 75, 2$ G, $A_z = 165$ G for **7,** $g_x = g_y = 1.978$, $g_z = 1.942$, $A_x = A_y = 65.5$ G, $A_z = 180$ G for **8** and $g_x = g_y = 1.977$, $g_z = 1.958$, $A_x = A_y = 72$ G, $A_z = 169$ G for **9**.

Absorption spectroscopy

Table S12. The absorption maxima and molar extinction coefficients for **1-10** in two solvents and two concentrations

Compound	Medium / concentration	λ (nm) (ε ($M^{-1}cm^{-1}$))
**1**	DMSO 10^{-3} M	873 (27); 634 (15)
	DMSO 10^{-4} M	298 (14200); 274 (13600)
	MeOH 10^{-3} M	771 (26); 603 (17)
	MeOH 10^{-4} M	294 (12800); 270 (13700); 222 (19500)
**2**	DMSO 10^{-3} M	892 (32); 639 (18)
	DMSO 10^{-4} M	323 (13200); 312 (16000); 271 (6400)
	MeOH 10^{-3} M	771 (70); 596 (60)
	MeOH 10^{-4} M	307 (20900); 274 (9400); 237 (14100); 225 (24200)
**3**	DMSO 10^{-3} M	828 (29); 619 (18); 447 (170)
	DMSO 10^{-4} M	338 (1600); 292 (7800); 263 (18300)
	MeOH 10^{-3} M	778 (1000); 598 (470); 394 (850)
	MeOH 10^{-4} M	294 (4100); 269 (8000); 252 (10900); 231 (11300)
**4**	DMSO 10^{-3} M	891 (31); 471 (140)
	DMSO 10^{-4} M	388 (540); 347 (880); 329 (2000); 300 (20700); 281 (30500); 271 (27500)
	MeOH 10^{-3} M	766 (26); 395 (620)
	MeOH 10^{-4} M	364 (360); 331 (700); 297 (7100); 265 (29100); 236 (26000); 208 (35600)
**5**	DMSO 10^{-3} M	779 (33); 580 (23); 443 (590)
	DMSO 10^{-4} M	340 (4480); 311 (13600); 299 (13800); 271 (28800)
	MeOH 10^{-3} M	766 (38); 588 (26); 436 (410)
	MeOH 10^{-4} M	338 (6100); 305 (13500); 260 (48000), 214 (51500)
**6**	DMSO 10^{-3} M	743 (47); 478 (740)
	DMSO 10^{-4} M	382 (1600); 289 (29500); 279 (33000)
	MeOH 10^{-3} M	733 (62); 457 (750)
	MeOH 10^{-4} M	367 (2700); 282 (39400); 212 (48600)
**7**	DMSO 10^{-3} M	880 (22); 737 (19); 467 (450)
	DMSO 10^{-4} M	325 (9100); 302 (18100); 284 (26300); 274 (25000)
	MeOH 10^{-3} M	698 (46); 430 (1800)
	MeOH 10^{-4} M	321 (5300); 299 (11900); 269 (35700); 251 (58500); 233 (47100); 210 (43700)
**8**	DMSO 10^{-3} M	875 (21); 744 (20); 456 (220)
	DMSO 10^{-4} M	362 (490); 292 (7500); 272 (26300)
	MeOH 10^{-3} M	722 (64); 441 (440); 356 (1000)
	MeOH 10^{-4} M	292 (6900); 267 (23000); 226 (36600); 211 (30500)
**9**	DMSO 10^{-3} M	888 (10); 641(4)
	DMSO 10^{-4} M	381 (260); 340 (300); 279 (16000); 263 (11500)
	MeOH 10^{-3} M	750 (43); 581 (29); 390 (800)
	MeOH 10^{-4} M	307 (2700); 276 (11900); 226 (12900)
**10**	DMSO 10^{-3} M	888 (26); 641(9)
	DMSO 10^{-4} M	383 (200); 325 (330); 269 (5300)
	MeOH 10^{-3} M	820 (40); 603 (19)
	MeOH 10^{-4} M	361 (100); 308 (340); 267 (5300); 220 (13800)

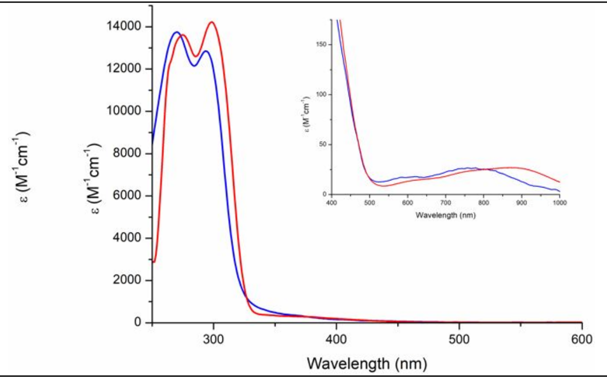

Complex 1

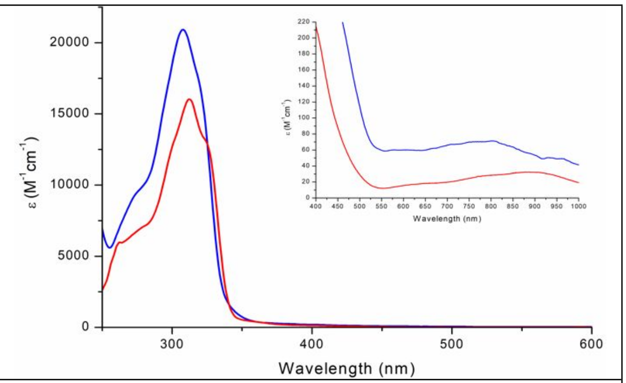

Complex 2

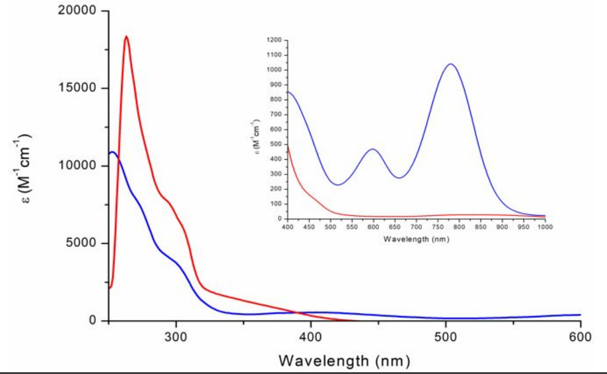

Complex 3

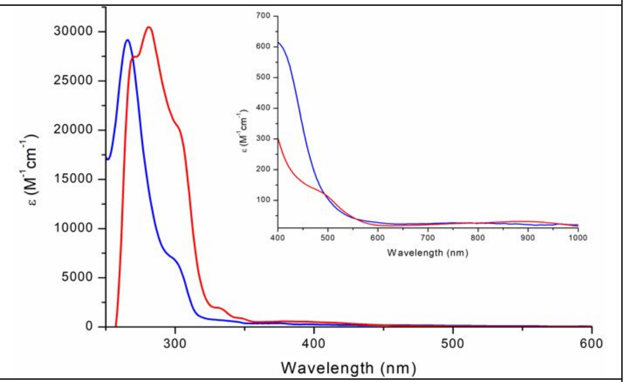

Complex 4

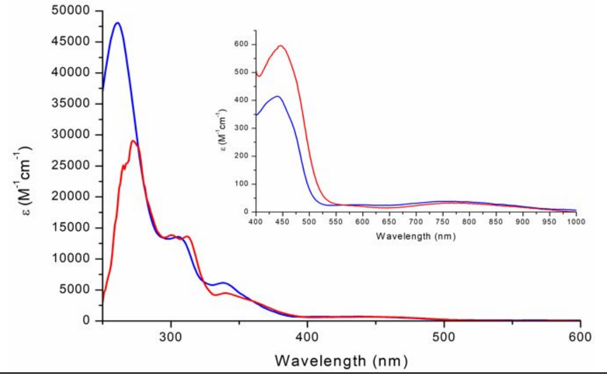

Complex 5

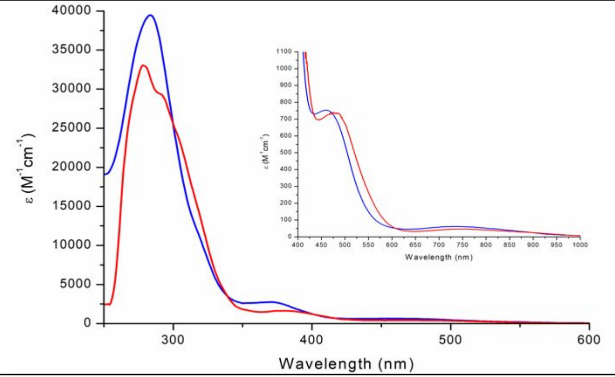

Complex 6

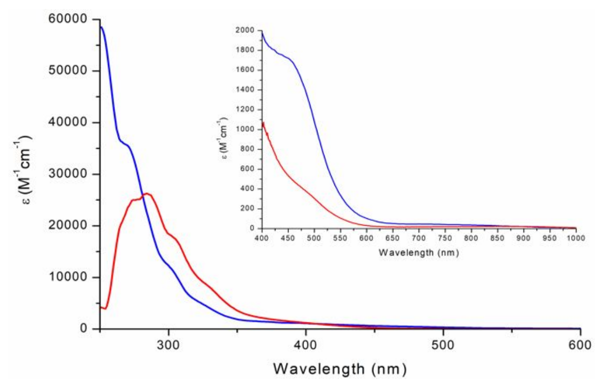

Complex 7

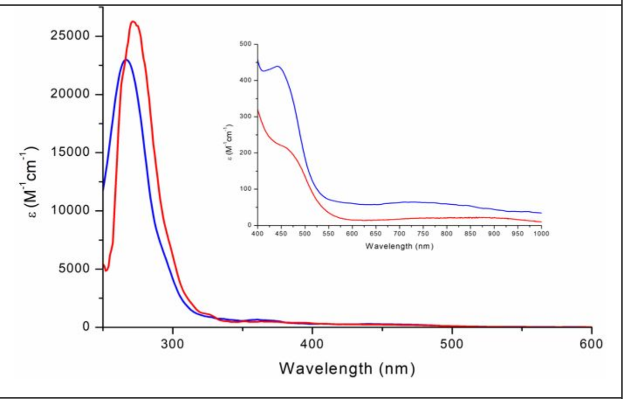

Complex 8

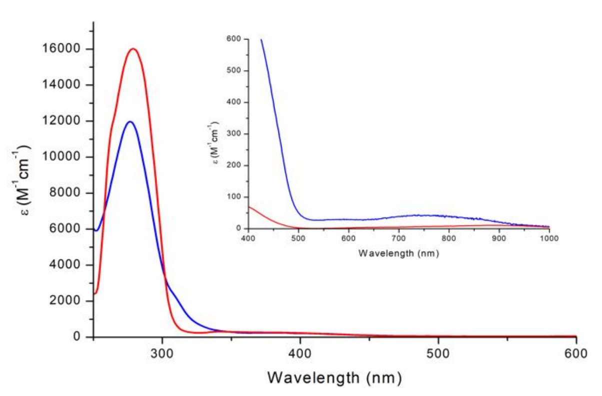

Complex 9

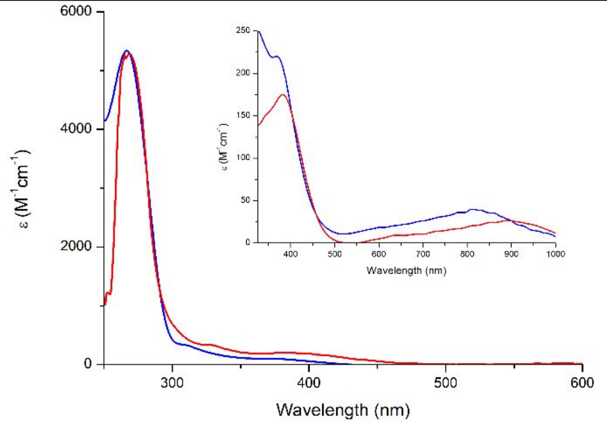

Complex 10

Figure S11. Absorption spectra of **1–10** in DMSO (red) and MeOH (blue), concentration 10^{-4} M. Inset: close-up of the 400-1000 nm region of spectra, concentration 10^{-3} M

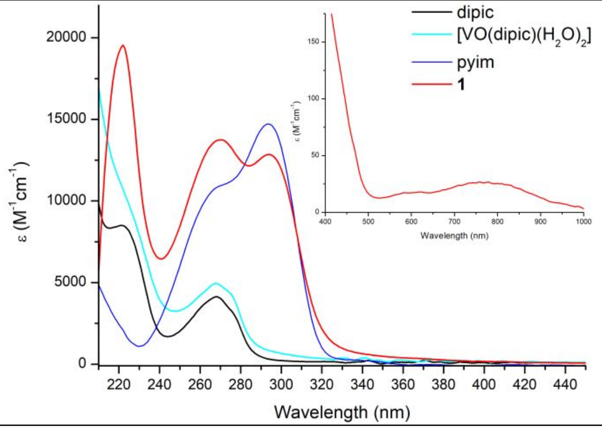

Complex 1

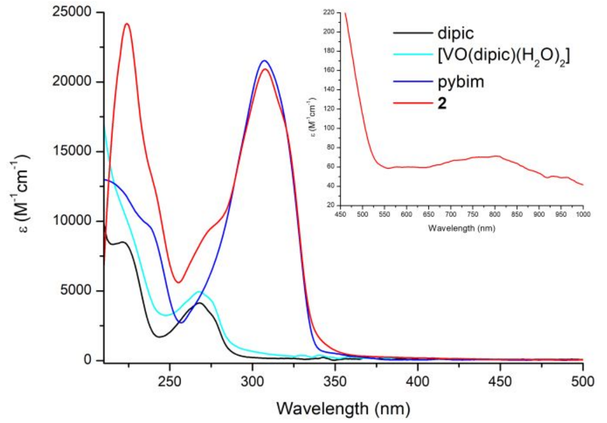

Complex 2

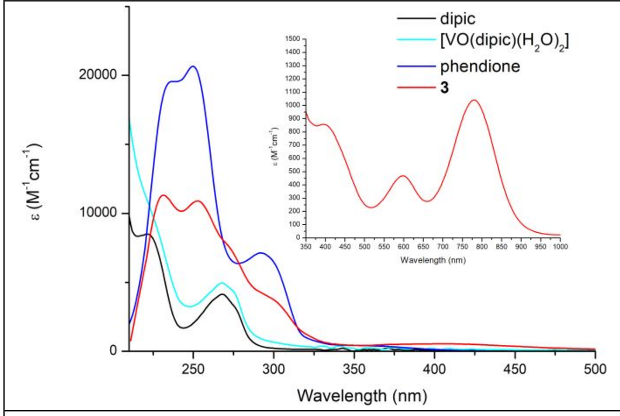

Complex 3

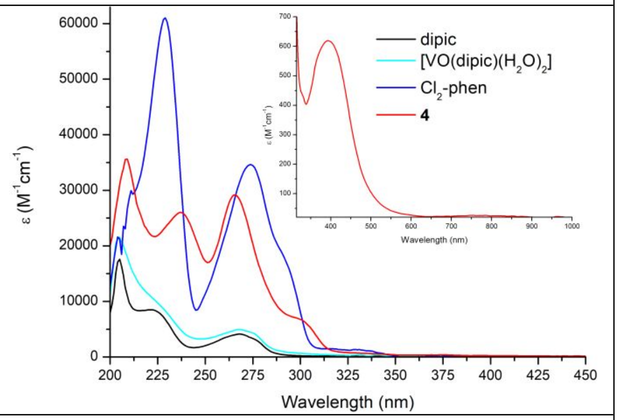

Complex 4

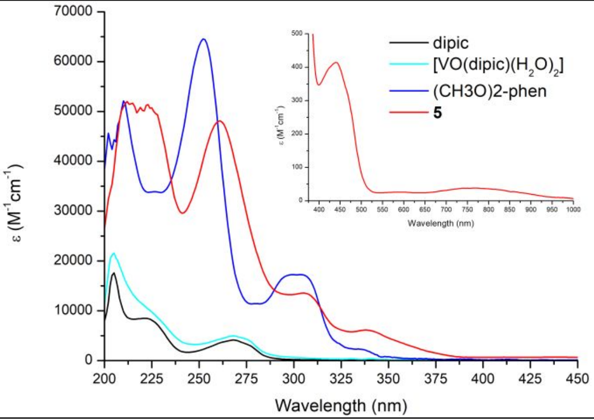

Complex 5

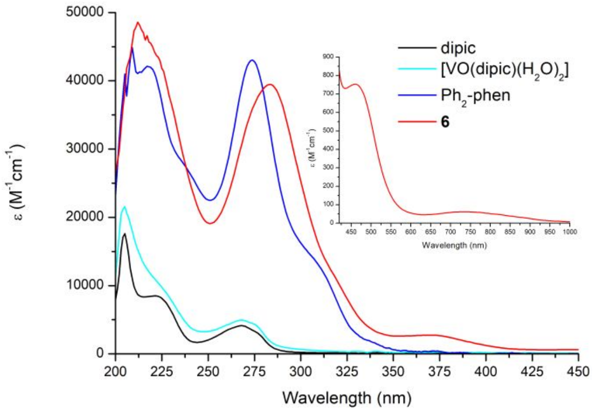

Complex 6

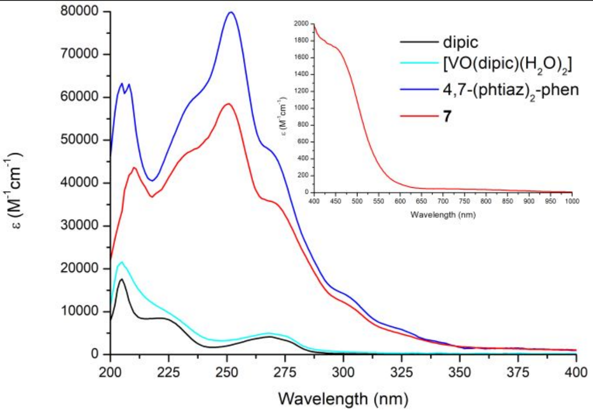

Complex 7

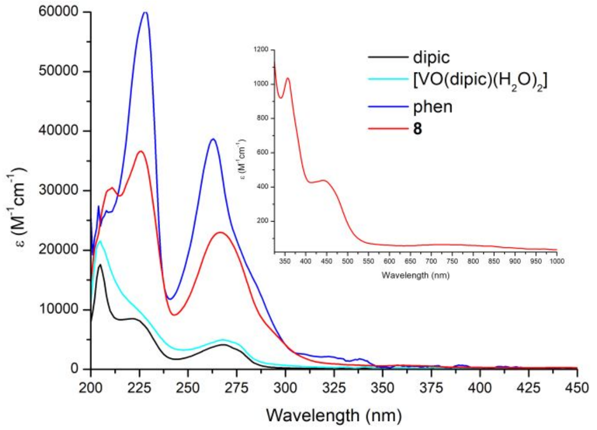

Complex 8

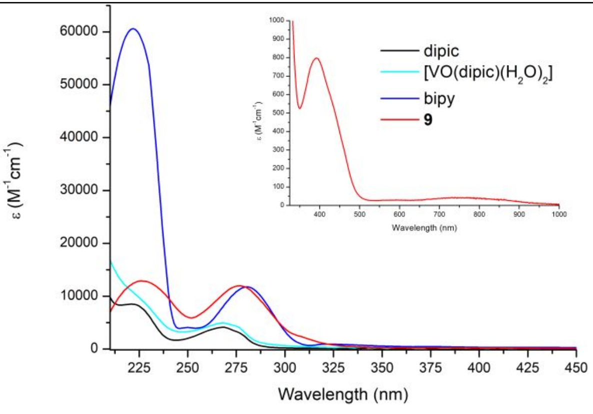

Complex 9

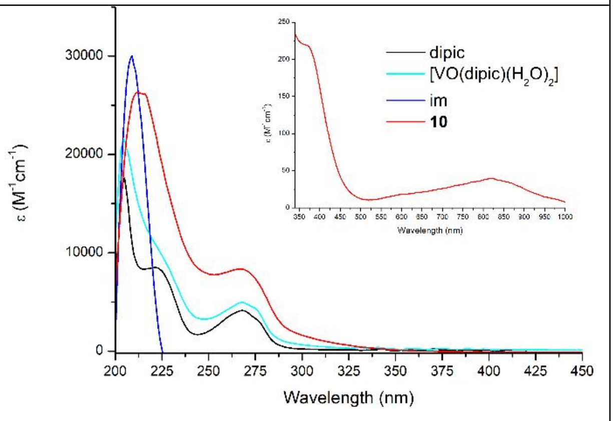

Complex 10

Figure S12. Absorption spectra of **1–10** in methanol (red) in comparison to the spectra of the dipicolinic acid (black), [VO(dipic)(H_2O)$_2$] (light blue) and corresponding phen-based ligand (dark blue). Concentration of dipic and [VO(dipic)(H_2O)$_2$] = 5×10^{-5}M, concentration of ligands = 10^{-5}M, concentration of vanadium(V) complexes: 5×10^{-5}M (main graphs) and 10^{-3}M (insets)

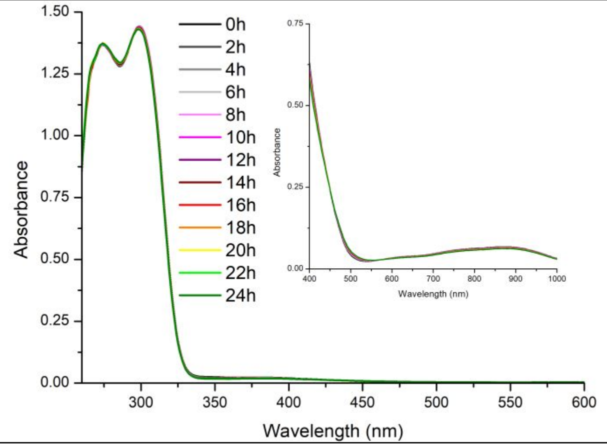

Complex 1

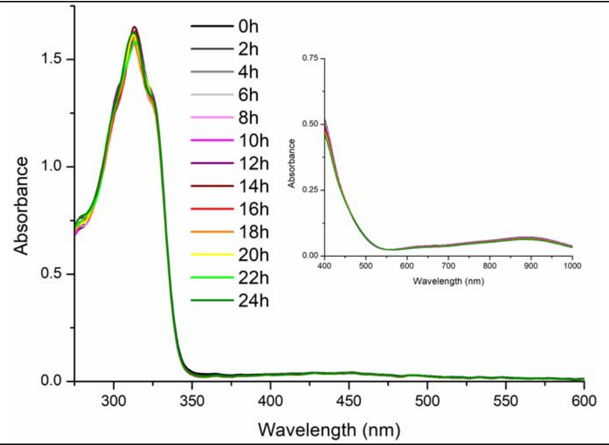

Complex 2

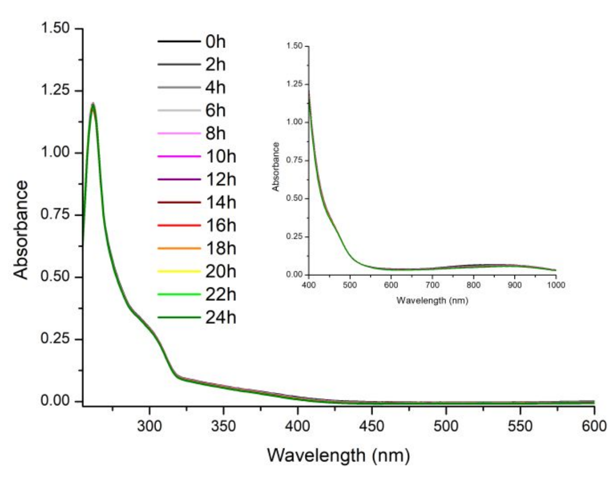

Complex 3

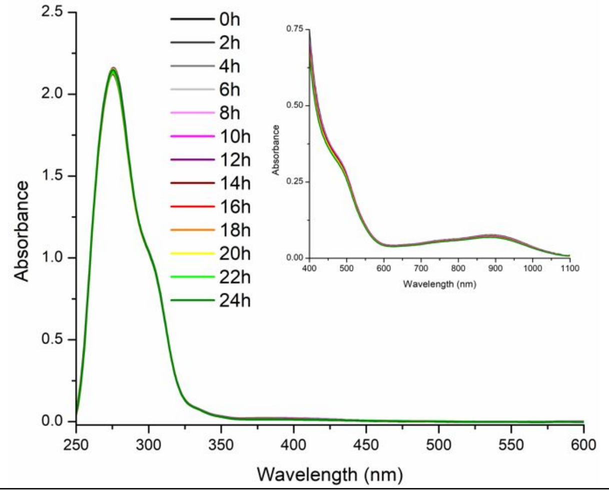

Complex 4

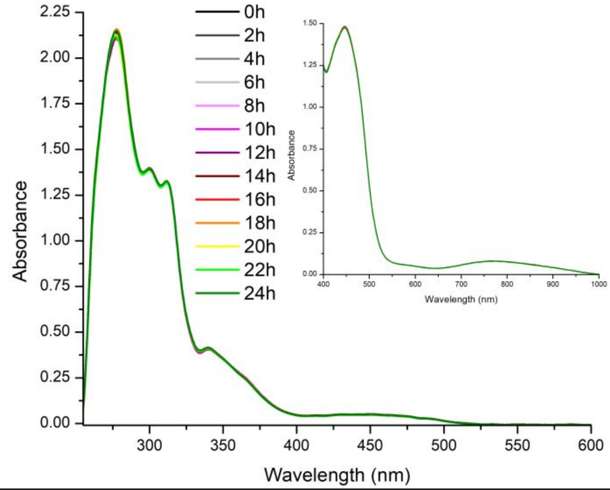

Complex 5

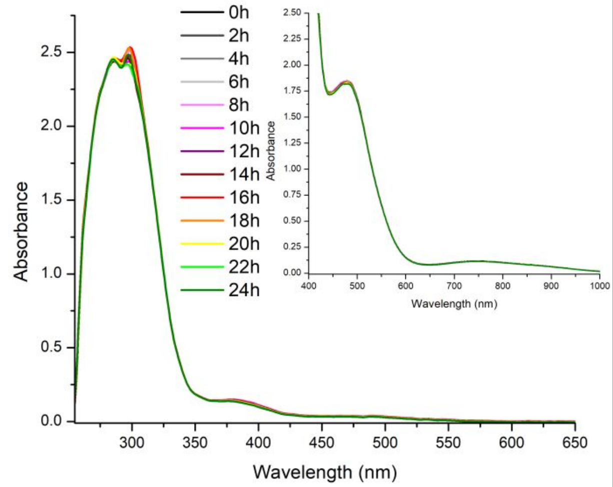

Complex 6

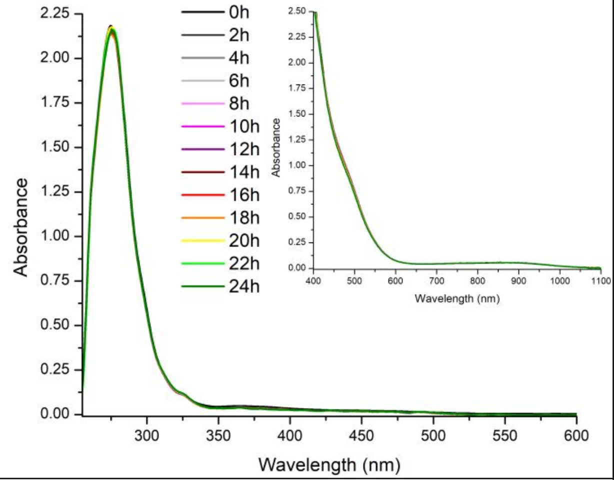

Complex 7

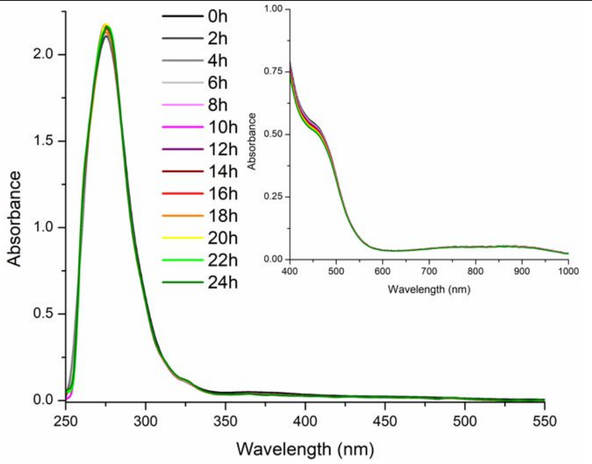

Complex 8

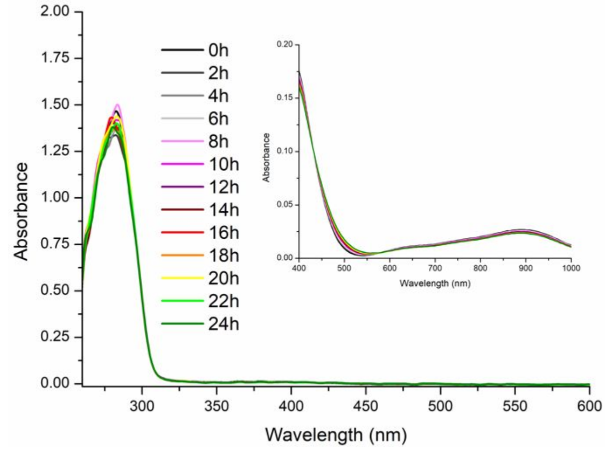

Complex 9

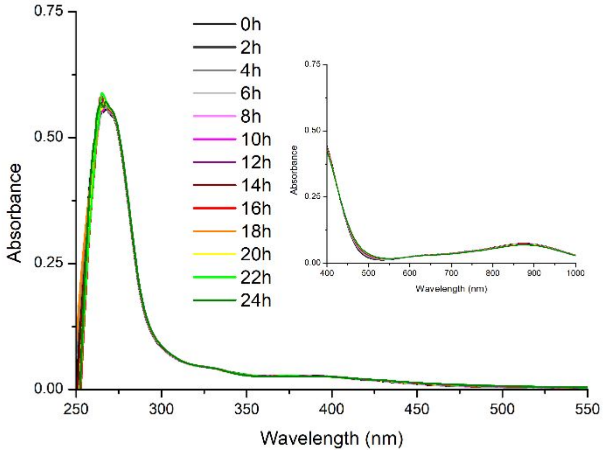

Complex 10

Figure S13. UV-Vis stability spectra in DMSO for compounds **1-10**. Spectra were recorded every 2 hours over 24h. Concentration of vanadium(V) complexes: 10^{-4}M (main graphs) and 2.5×10^{-3}M (insets)

Biological studies

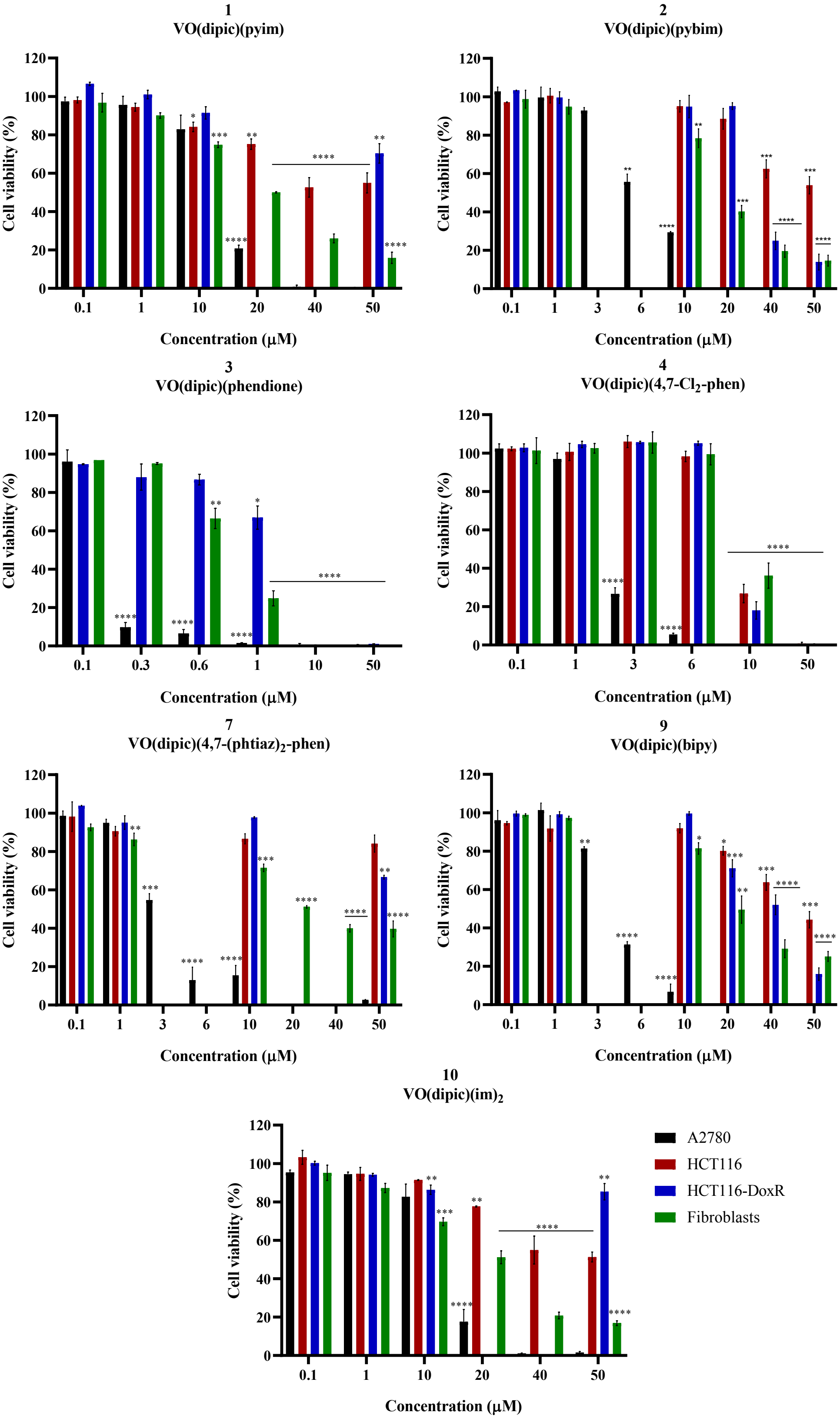

Figure S14. Cell viability of A2780, HCT116, HCT116-DoxR and fibroblasts cell lines after exposure to different concentrations of the vanadium complexes **1–4**, **7**, **9** and **10** for 48 hours. 0.1% (v/v) DMSO was used as the vehicle control. Data are expressed as the mean ± SEM of at least two independent assays (* $p < 0.05$; ** $p < 0.01$; *** $p < 0.001$; **** $p < 0.0001$).

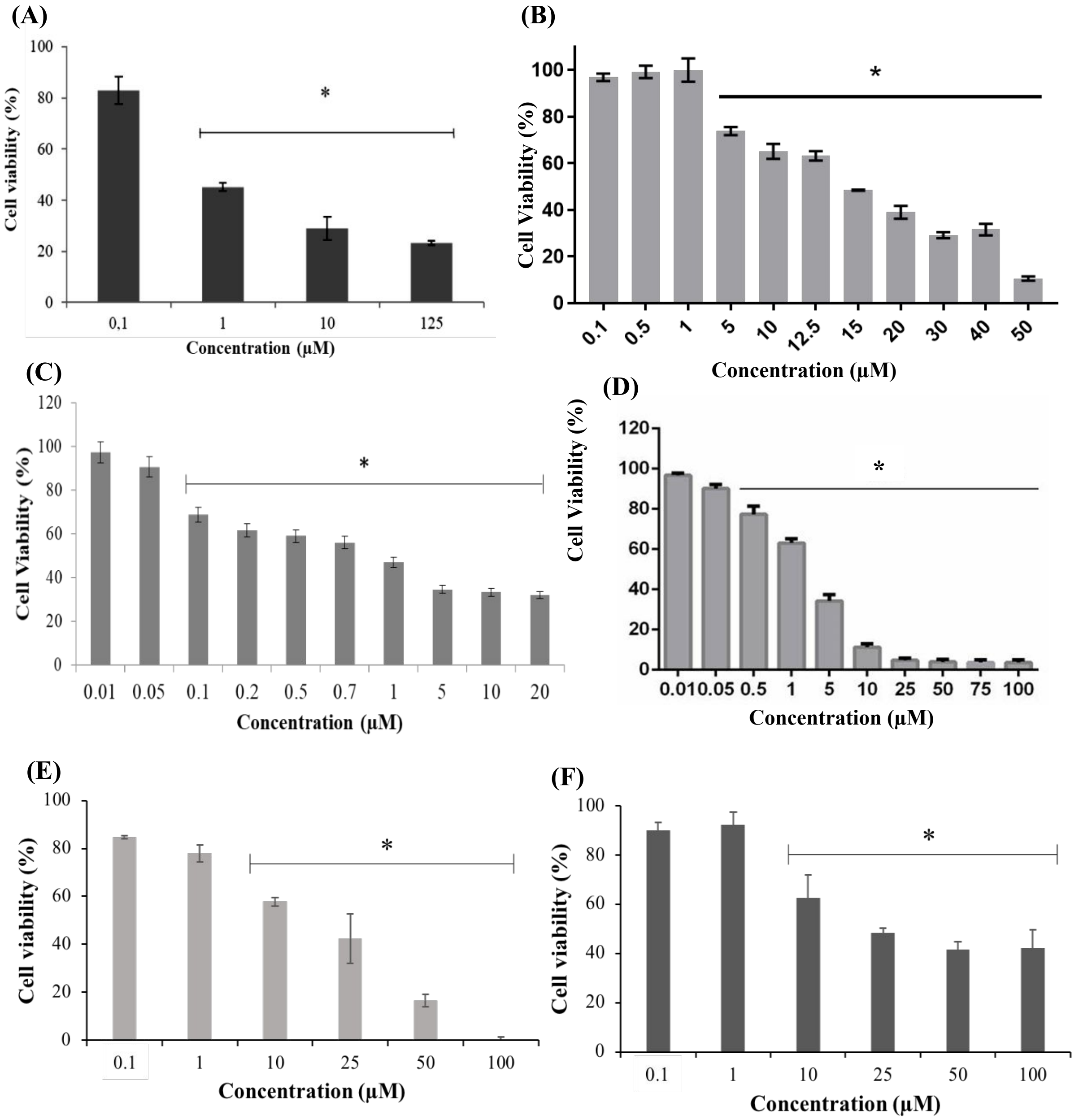

Figure S15. Viability of HCT116 cancer cell line after exposure to different concentrations of doxorubicin **(A)** and cisplatin **(B)** for 48 hours. Viability of A2780 cancer cell line after exposure to different concentrations of doxorubicin **(C)** and cisplatin **(D)** for 48 hours. Viability of Fibroblasts after exposure to different concentrations of doxorubicin **(E)** and cisplatin **(F)**. 0.1% (v/v) DMSO was used as the vehicle control. Data are expressed as the mean ± SEM of three biological assays.

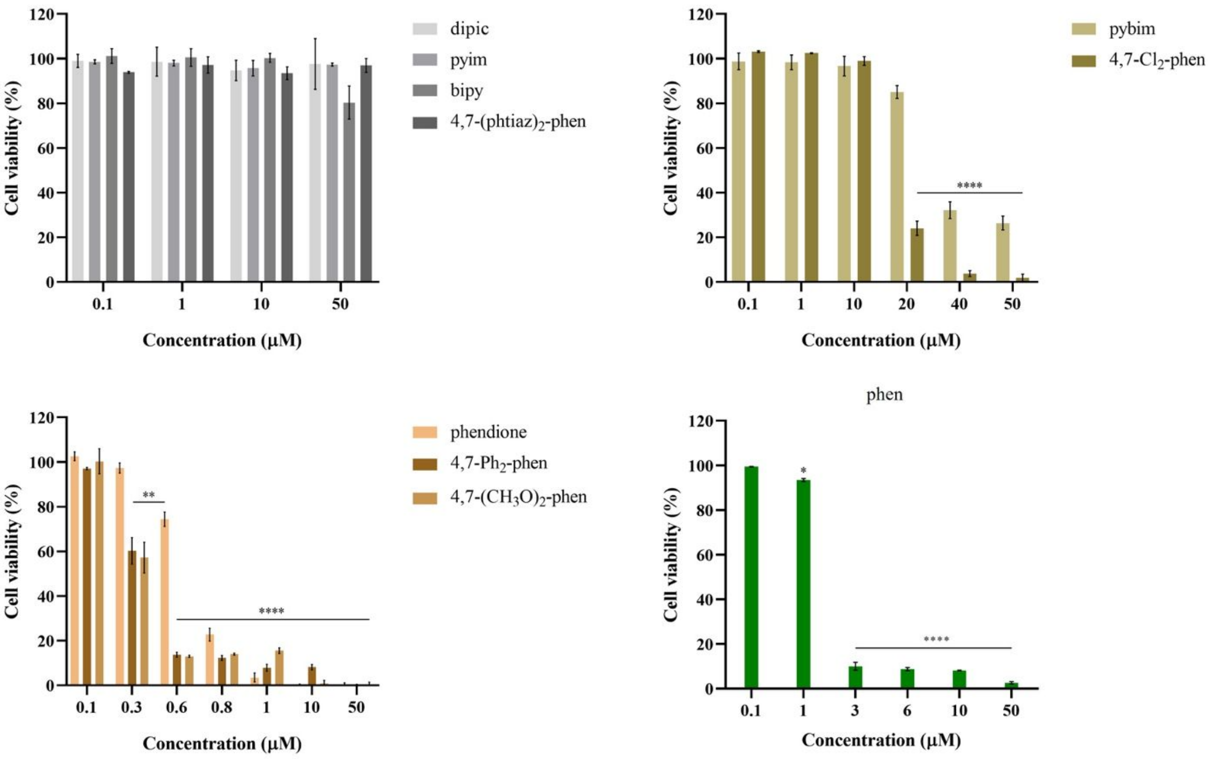

Figure S16. Cell viability of HCT116-DoxR cells after exposure to different concentrations of the vanadium complexes' ligands for 48 hours. 0.1% (v/v) DMSO was used as the vehicle control. Data are expressed as the mean ± SEM of at least two independent assays (* $p < 0.05$; ** $p < 0.01$; **** $p < 0.0001$).

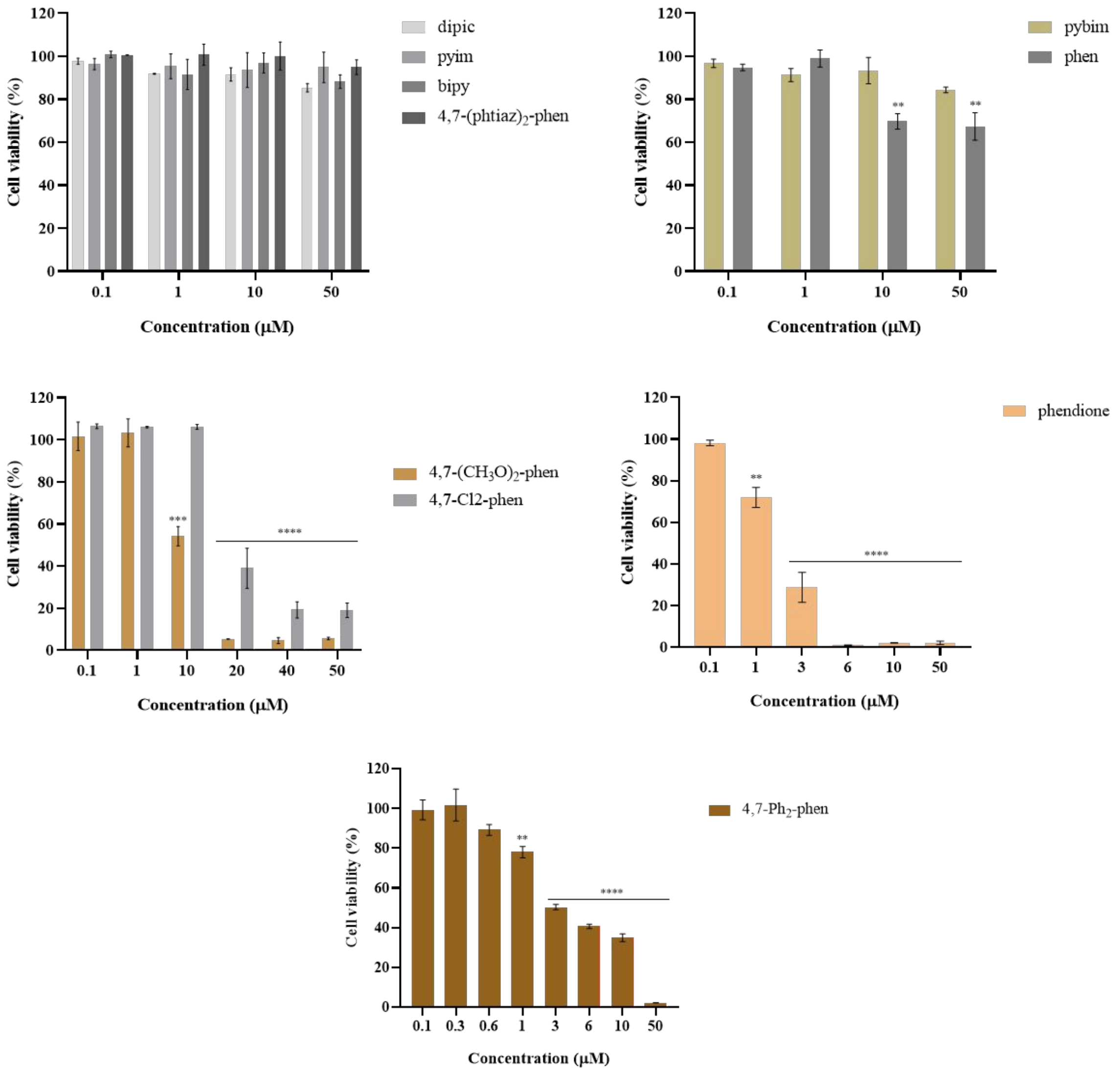

Figure S17. Cell viability of fibroblasts after exposure to different concentrations of the vanadium complexes' ligands for 48 hours. 0.1% (v/v) DMSO was used as the vehicle control. Data are expressed as the mean ± SEM of at least two independent assays (** $p < 0.01$; *** $p < 0.001$; **** $p < 0.0001$).

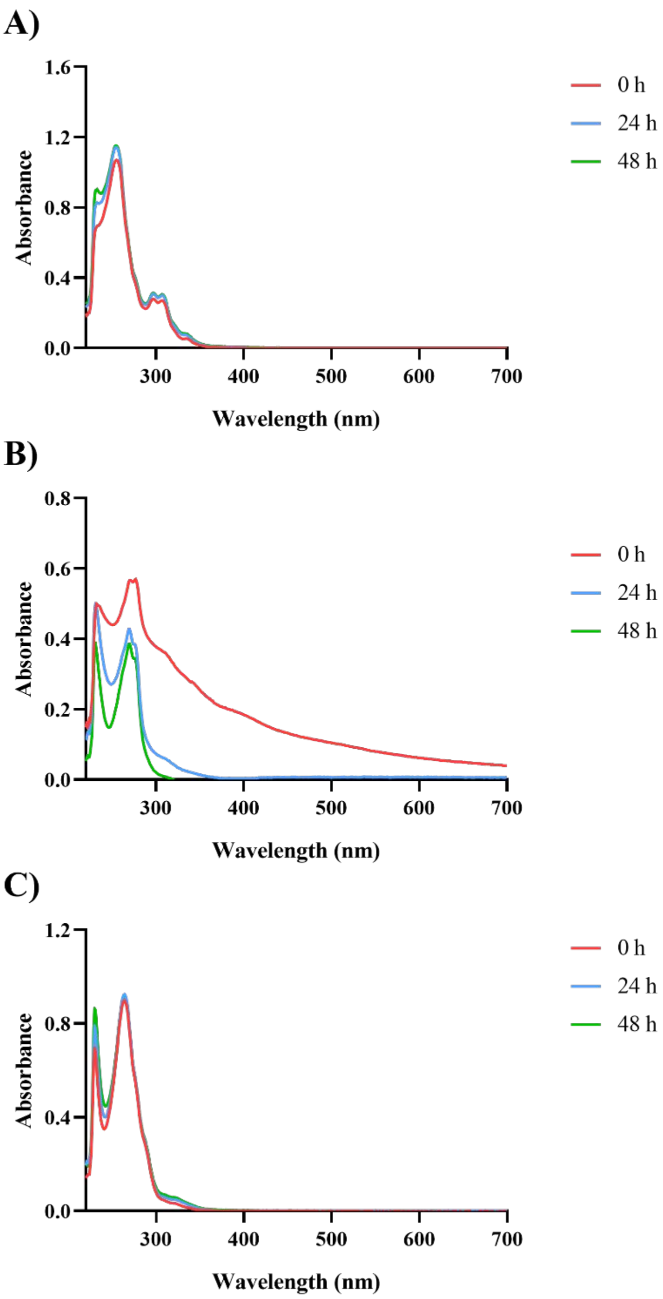

Figure S18. Evaluation of the stability and solubility of complexes **5 (A)**, **6 (B)** and **8 (C)** by UV-Vis spectroscopy over 48 hours. Absorbance spectra of 25 µM of complexes **5** and **8** and 50 µM of complex **6** in RPMI culture medium (previously diluted in DMSO) at different incubation times: 0 hours (red), 24 hours (blue) and 48 hours (green).

Table S13. Data obtained with the ICP-AES experiment after 3 hours of exposure of HCT116-DoxR cells to 10x IC_{50} of complexes **5**, **6** and **8**. Table presents the concentration of complexes administered in cells (10x IC_{50}), the complexes' concentration present in cells after the several washings and the respective complexes internalized percentage.

Complex	10x IC_{50} (µM)	Concentration in cells (µM)	Internalized percentage (%)
**5**	1.613	1.590	98.55
**6**	1.701	1.672	98.32
**8**	12.600	6.705	53.21

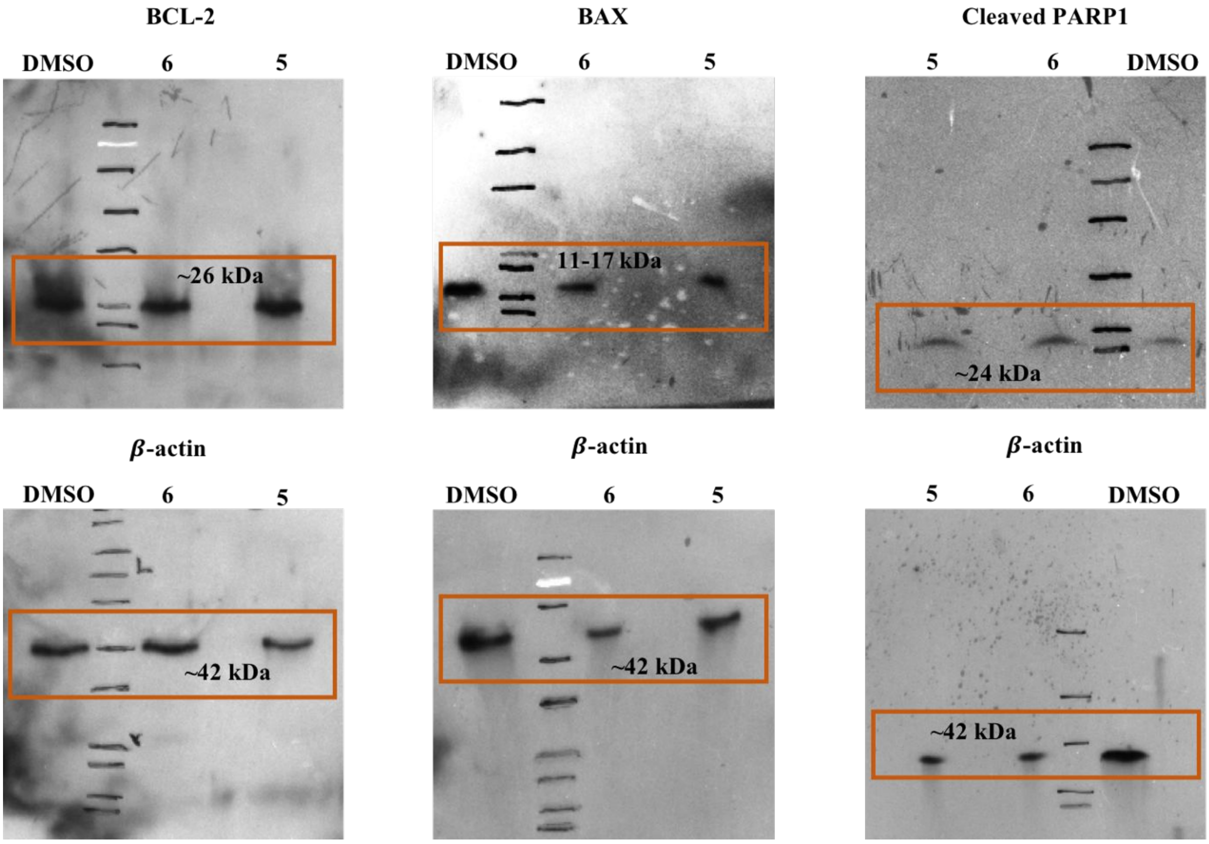

Figure S19. Western Blot bands used for the quantification of proteins BAX, BCL-2 and cleaved PARP1 in HCT116-DoxR cells after their exposure to complexes **5** and **6**, or DMSO.

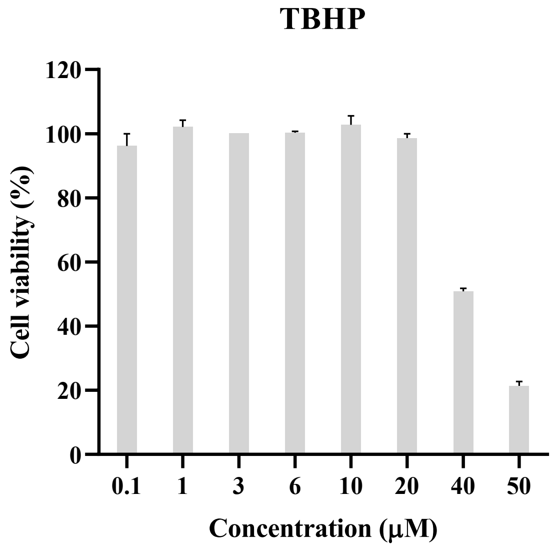

Figure S20. Viability of HCT116-DoxR cells after exposure to different concentrations of TBHP for 48 hours. 0.1% (v/v) DMSO was used as the vehicle control. Data are expressed as the mean ± SEM of one biological assay.

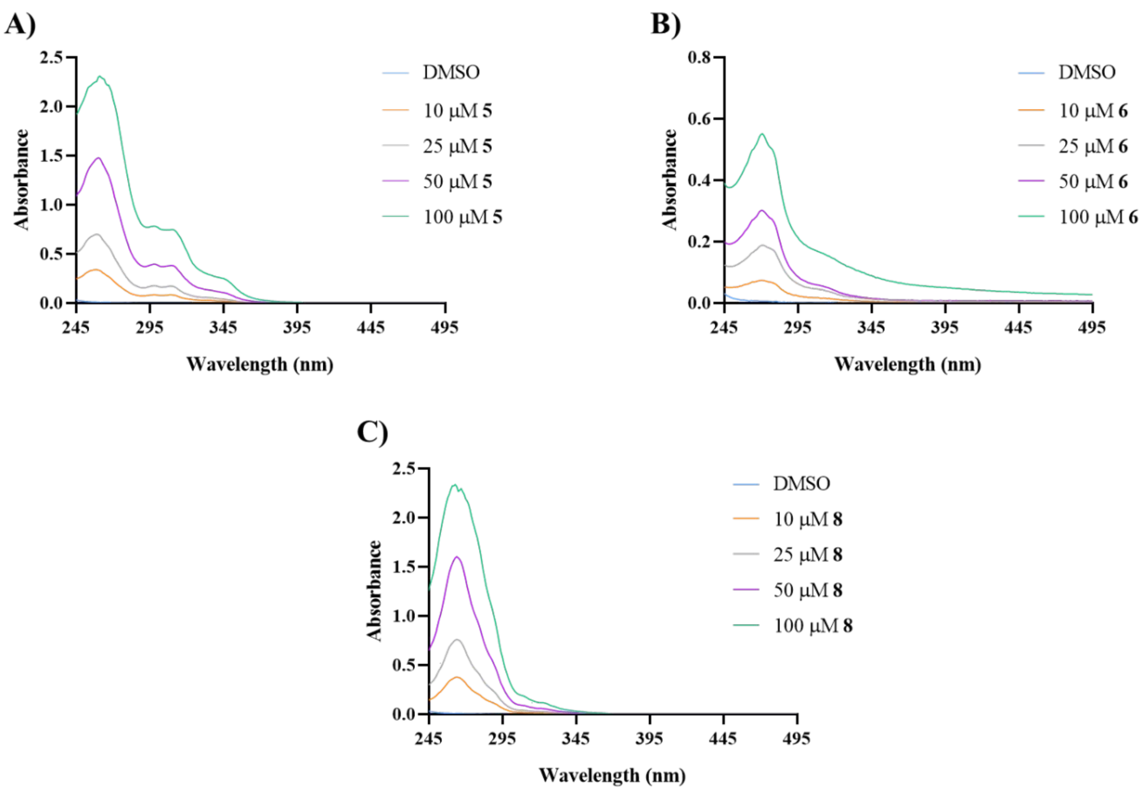

Figure S21. UV-Vis spectra 24 h after exposure to different concentrations of complexes **5 (A)**, **6 (B)** and **8 (C)** or DMSO (used as control).

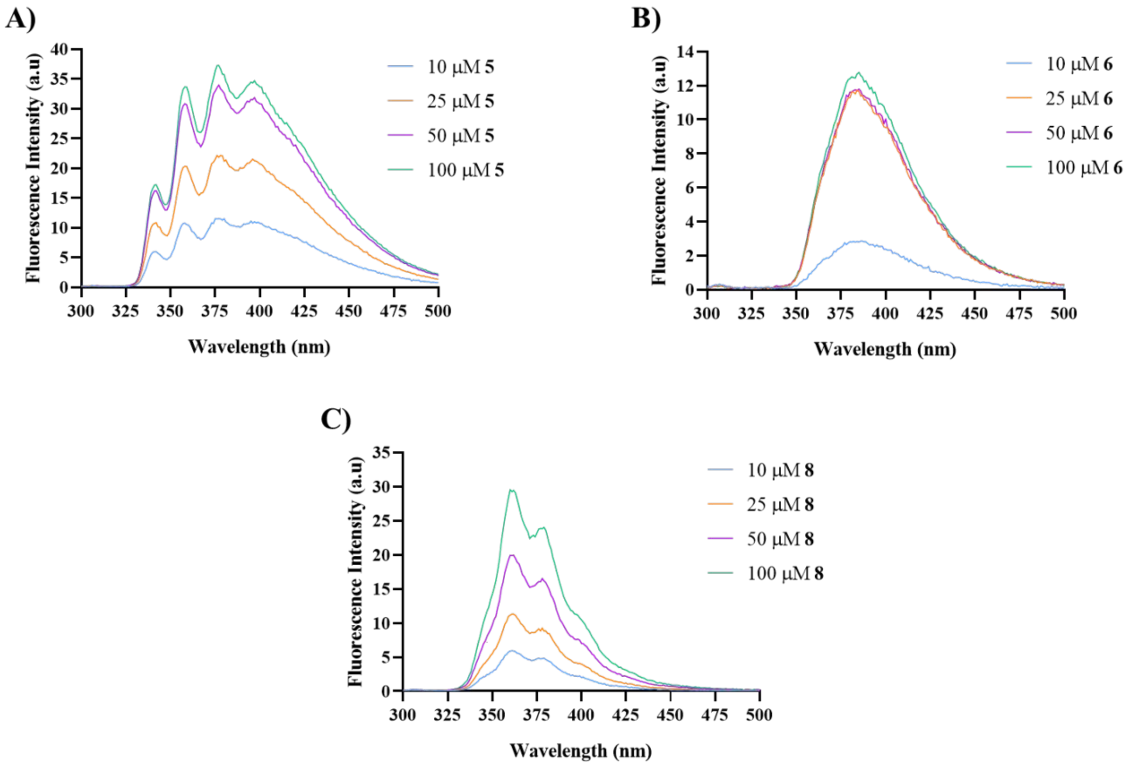

Figure S22. Fluorescence spectra 24h after exposure to different concentrations of complexes **5 (A)**, **6 (B)** and **8 (C)**.

References

(1) CrysAlis PRO, Oxford Diffraction/Agilent Technologies UK Ltd, Yarnton, England, 2014.

(2) Sheldrick, G. M. Crystal Structure Refinement with SHELXL. Acta Cryst C 2015, 71 (1), 3–8. https://doi.org/10.1107/S2053229614024218.

(3) Spek, A. L. PLATON SQUEEZE: A Tool for the Calculation of the Disordered Solvent Contribution to the Calculated Structure Factors. Acta Cryst C 2015, 71 (1), 9–18. https://doi.org/10.1107/S2053229614024929.

(4) Ozarowski, A. DoubletExact program; NHMFL, Florida University: Tallahassee, USA

(5) Andrés, A.; Rosés, M.; Ràfols, C.; Bosch, E.; Espinosa, S.; Segarra, V.; Huerta, J.M. Setup and validation of shake-flask procedures for the determination of partition coefficients (logD) from low drug amounts. Eur. J. Pharm. Sci., 2015, 76, 181–191. https://doi.org/10.1016/j.ejps.2015.05.008.

(6) Czyrski, A. The spectrophotometric determination of lipophilicity and dissociation constants of ciprofloxacin and levofloxacin, Spectrochim. Acta A Mol. Biomol. Spectrosc., 2022, 265, 120343. https://doi.org/10.1016/j.saa.2021.120343.

(7) Pedrosa, P.; Mendes, R.; Cabral, R.; Martins, L. M. D. R. S.; Baptista, P. V.; Fernandes, A. R. Combination of Chemotherapy and Au-Nanoparticle Photothermy in the

Visible Light to Tackle Doxorubicin Resistance in Cancer Cells. Sci Rep 2018, 8 (1), 11429. https://doi.org/10.1038/s41598-018-29870-0.

(8) van Engeland, M.; Nieland, L. J. W.; Ramaekers, F. C. S.; Schutte, B.; Reutelingsperger, C. P. M. Annexin V-Affinity Assay: A Review on an Apoptosis Detection System Based on Phosphatidylserine Exposure. Cytometry 1998, 31 (1), 1–9. https://doi.org/10.1002/(SICI)1097-0320(19980101)31:1<1::AID-CYTO1>3.0.CO;2-R.

(9) Niles, A. L.; Moravec, R. A.; Riss, T. L. Update on in Vitro Cytotoxicity Assays for Drug Development. Expert Opinion on Drug Discovery 2008, 3 (6), 655–669. https://doi.org/10.1517/17460441.3.6.655.

(10) Das, K.; Beyene, B. B.; Datta, A.; Garribba, E.; Roma-Rodrigues, C.; Silva, A.; Fernandes, A. R.; Hung, C.-H. EPR and Electrochemical Interpretation of Bispyrazolylacetate Anchored Ni(II) and Mn(II) Complexes: Cytotoxicity and Anti-Proliferative Activity towards Human Cancer Cell Lines. New J. Chem. 2018, 42 (11), 9126–9139. https://doi.org/10.1039/C8NJ01033A.

(11) Choroba, K.; Machura, B.; Szlapa-Kula, A.; Malecki, J. G.; Raposo, L.; Roma-Rodrigues, C.; Cordeiro, S.; Baptista, P. V.; Fernandes, A. R. Square Planar Au(III), Pt(II) and Cu(II) Complexes with Quinoline-Substituted 2,2′:6′,2″-Terpyridine Ligands: From in Vitro to in Vivo Biological Properties. European Journal of Medicinal Chemistry 2021, 218, 113404. https://doi.org/10.1016/j.ejmech.2021.113404.

(12) Reigosa-Chamorro, F.; Raposo, L. R.; Munín-Cruz, P.; Pereira, M. T.; Roma-Rodrigues, C.; Baptista, P. V.; Fernandes, A. R.; Vila, J. M. In Vitro and In Vivo Effect of Palladacycles: Targeting A2780 Ovarian Carcinoma Cells and Modulation of Angiogenesis. Inorg. Chem. 2021, 60 (6), 3939–3951. https://doi.org/10.1021/acs.inorgchem.0c03763.

(13) Lenis-Rojas, O. A.; Cabral, R.; Carvalho, B.; Friães, S.; Roma-Rodrigues, C.; Fernández, J. A. A.; Vila, S. F.; Sanchez, L.; Gomes, C. S. B.; Fernandes, A. R.; Royo, B. Triazole-Based Half-Sandwich Ruthenium(II) Compounds: From In Vitro Antiproliferative Potential to In Vivo Toxicity Evaluation. Inorg. Chem. 2021, 60 (11), 8011–8026. https://doi.org/10.1021/acs.inorgchem.1c00527.

(14) Uden, P.C.; Zeng, Y. A study of diastereoisomerism of oxovanadium schiff base chelates by gas chromatography-atomic emission spectroscopy, and high performance liquid chromatography. Chromatographia, 1992, 34, 269–275. https://doi.org/10.1007/BF02268357.

(15) Khuhawar, M.Y.; Soomro, A. I. Gas and liquid chromatographic studies of copper(II), nickel(II), palladium(II) and oxovanadium(IV) chelates of some fluorinated ketoamine Schiff bases, J. Chromatogr. A, 1993, 639, 371-374. 10.1016/0021-9673(93)80279-H.

(16) Jiang, Z.; Ou, Z.; Chen N.; Jundong Wang, J.; Huang, J.; Shao, J.; K. M. Synthesis, spectral and electrochemical characterization of non-aggregating α-substituted vanadium(IV)-oxo phthalocyanine, J. Porphyrins Phthalocyanines 2005; 9: 352-360. 10.1142/S1088424605000447.

(17) Xie, M.-j.; Li, L.; Yang, X.-D.; Liu, W.-p.; Yan, S.-p.; Niu, Y.-f.; Meng, Z.-h.; A new insulin-enhancing agent: [N,N′-bis(4-hydroxysalicylidene)-o-phenylene-diamine]oxovanadium(IV) and its permeability and cytotoxicity, Eur. J. Med. Chem., 2010, 45, 2327-2335. 10.1016/j.ejmech.2010.02.010.
